# Enantiomerically Pure *ansa*-*η*^5^-Complexes of Transition Metals as an Effective Tool for Chirality Transfer

**DOI:** 10.3390/molecules30122511

**Published:** 2025-06-08

**Authors:** Pavel V. Kovyazin, Leonard M. Khalilov, Lyudmila V. Parfenova

**Affiliations:** Institute of Petrochemistry and Catalysis, Ufa Federal Research Center, Russian Academy of Sciences, 141 Prospekt Oktyabrya, 450075 Ufa, Russia; kpv38@mail.ru (P.V.K.); khalilovlm@gmail.com (L.M.K.)

**Keywords:** *ansa*-metallocenes, enantiomerically pure complexes, stereoselectivity, asymmetric synthesis, reaction mechanisms

## Abstract

Chiral *ansa*-*η*^5^-complexes of transition metals have shown remarkable efficacy in organometallic synthesis and catalysis. Additionally, enantiomerically pure *ansa*-complexes hold promise for the development of novel chiral materials and pharmaceuticals. The discovery and synthesis of a diverse range of group IVB and IIIB metal complexes represents a significant milestone in the advancement of stereoselective catalytic methods for constructing metal-C, C-C, C-H, and C-heteroatom bonds. The synthesis of enantiomerically pure metallocenes can be accomplished through several strategies: utilizing optically active precursors of *η*^5^-ligands, separation of diastereomers of complexes with enantiomerically pure agents, and synthesis via the stereocontrolled reactions of enantiomerically pure σ-complexes with prochiral anions of *η*^5^-ligands. This review focuses on the analysis of various nuances of the synthesis of enantiomerically pure *ansa*-*η*^5^-complexes of titanium and lanthanum families. Their applicability as effective catalysts in asymmetric carbomagnesiation, carbo- and cycloalumination, oligo- and polymerization, Diels–Alder cycloaddition, reactions of zirconaaziridines, cyclization, hydrosilylation, hydrogenation, hydroamination, and other processes are highlighted as well.

## 1. Introduction

The discovery of metallocene compounds in the mid-20th century stimulated the development of several significant areas within organometallic chemistry [1,2]. Metallocenes have exhibited outstanding catalytic properties in the formation of metal–carbon (M-C), carbon–carbon (C-C), and carbon–hydrogen (C-H) bonds. Among these, single-site alkene polymerization using catalytic systems composed of IV subgroup metal *η*^5^-complexes and organoaluminum or -boron compounds has emerged as a particularly promising avenue [3,4,5,6,7,8,9,10,11]. Furthermore, various catalytic methods of alkene and acetylene transformations, such as di- and oligomerization [12,13,14,15,16,17,18,19], hydrometalation [20,21,22,23], carbometalation [24,25,26,27], and cyclometalation [21,24,28,29,30], are well known. Particular interest is drawn to the application of metallocenes as biosensors, antitumor agents, and other pharmaceuticals in medicine [31,32,33,34,35].

The high activity, chemo-, and stereoselectivity of metallocene complexes can be attributed to their unique structural characteristics. These compounds effectively ensure the stability of the electronic and steric environment of the transition metal center due to the high bond energy of the metal–ligand interactions. Additionally, they allow for variations in electrophilicity and geometry of the catalytically active sites through extensive structural modifications of the *π*-ligands. The introduction of bridging groups has led to the development of *ansa*-complexes with distinct properties. The global literature describes the synthesis of numerous *ansa*-metallocene complexes [36,37,38,39,40], which have found diverse applications in organic and organometallic chemistry. Notably, the binding of *η*^5^-ligands increases electrophilicity and accessibility of the catalytically active center by altering the coordination geometries of the *π*-ligands on the metal, while also fixing the geometry of the complex. This, combined with planar chirality, endows these compounds with significant potential for catalytic stereo- and enantioselective construction of C-C, C-H, C-N, and C-O bonds.

**Scheme 1:**
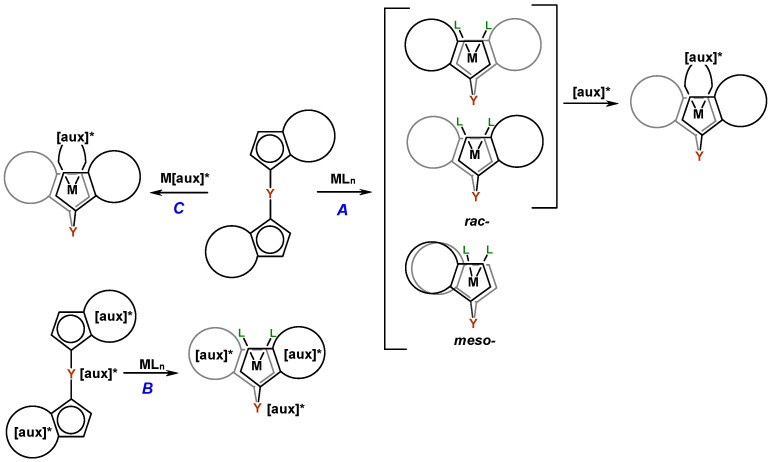
Pathways for the synthesis of enantiomerically pure *ansa*-complexes ([aux]*—chiral auxiliary).

The generation of stereoisomers of *ansa*-complexes is facilitated by the presence of both enantiotopic and diastereotopic faces in the bridging ligands, whose reaction with metal salts or complexes is accompanied by a stereodifferentiating effect [39,41]. Stereoisomers can be synthesized through various pathways. First, when using nonchiral metal salts or complexes, additional separation of the racemic mixture of the resulting *ansa*-derivatives with an enantiomerically pure reagent is necessary (Scheme 1, path A). Second, both diastereoselective and enantioselective synthesis of *ansa*-complexes can be achieved from enantiomerically pure ligands that contain stereogenic centers in both the substituents at the *η*^5^-ligands and the bridging group (Scheme 1, path B). Third, the use of metal complexes with chiral elements in σ-substituents can determine stereoselectivity at the *π*-coordination stage of prochiral ligands (Scheme 1, path C).

Enantiomerically pure *ansa*-complexes of III and IV subgroup metals, characterized by fixed geometries of *η*^5^-ligands, have demonstrated high efficiency in generating stereogenic centers on carbon atoms in prochiral substrates such as alkenes, ketones, imines, etc. Several reviews in the literature have focused on the synthesis of optically active metallocenes and their applications in organic chemistry. Reviews [39,41] detail methods for synthesizing chiral metallocenes, including those with *ansa*-bound *η*^5^-ligands. Review [36] discusses the synthesis of enantiopure planar chiral organometallic complexes via diastereoselective complexation methodologies. Additionally, the mini-review [40] presents various approaches for the synthesis of chiral *ansa*-metallocene complexes of Ti, Zr, and Hf that incorporate carbon bridging groups. Insights into the use of chiral C_2_-symmetric complexes of Zr and Ti as catalysts for the asymmetric construction of C-C and C-heteroatom bonds are provided in review articles [25,42].

This review considers and systematizes information on the approaches to the synthesis of enantiomerically pure *ansa*-*η*^5^-complexes of group IIIB and IVB transition metals, as well as various aspects of their application for asymmetric induction in the construction of C-C, M-C, C-H, and C-heteroatom bonds.

## 2. Synthesis of Enantiomerically Pure *ansa*-*bis*-*η*^5^-Complexes of Group III and IV Transition Metals

### 2.1. Path A: Resolution of Racemic Complexes into Enantiomers via Derivatization

The primary method for achieving enantiomerically enriched *ansa*-metallocenes is the kinetic resolution of *rac*-isomers of complexes through the use of derivatizing reagents (Scheme 1, path A).

**Scheme 2:**
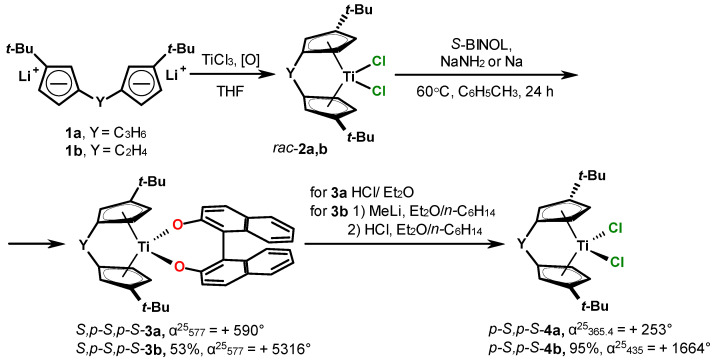
Derivatization of Ti complexes *rac*-**2a**,**b** using *S*-binaphthol.

In 1979, Brintzinger et al. first synthesized the optical isomer *p*-*S*, *p*-*S*-propandienyl-*bis*-(*tert*-butylcyclopentadienyl)titanium binaphtholate (*S*,*p*-*S*,*p*-*S*-**3a**) by the reaction of dichloride *rac*-complex **2a** with an equivalent amount of *S*-binaphthol (*S*-BINOL) in the presence of sodium amide in toluene (Scheme 2). The diastereomerically pure binaphtholate complex **3a** was isolated by column chromatography [43] and subsequently converted to the corresponding dichloride. Later, Collins et al. successfully resolved *rac*-ethylene-*bis*-*tert*-butylcyclopentadienyltitanium dichloride **2b** into its enantiomers using *S*-binaphthol [44].

**Scheme 3:**
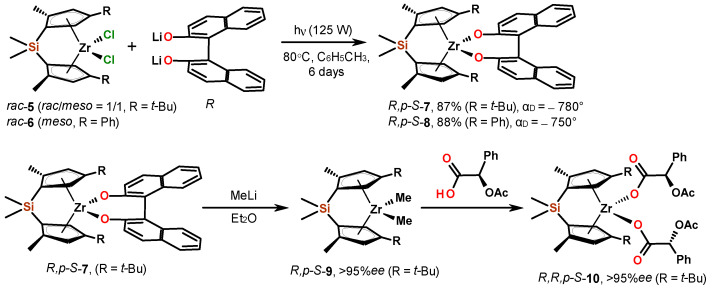
Derivatization of Zr complexes **5** and **6**.

A combination of photoisomerization and treatment with lithium *R*-binaphtholate was employed to obtain enantiomerically pure Zr *ansa*-cyclopentadienyl complexes featuring Si-bound ligands [45] (Scheme 3). This approach yielded compounds **7** and **8** with yields reaching up to 88% and enantiomeric purity exceeding 95%, as determined by ^1^H NMR spectroscopy of the carboxylate complex *R*,*R*,*p*-*S*-**10**.

**Scheme 4:**
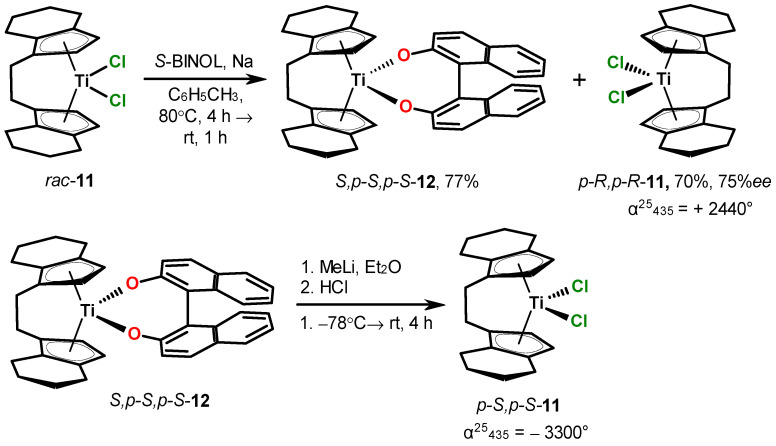
Derivatization of Ti *bis*-tetrahydroindenyl complex with *S*-binaphthol.

The enantiomers of the *bis*-tetrahydroindenyl complex *rac*-**11** were derivatized with *S*-binaphthol [46] (Scheme 4). This reaction produced two enantiomers, *S*,*p*-*S*,*p*-*S*-**12** and *p*-*R*,*p*-*R*-**11**, with yields of 77% and 70%, respectively, while the enantiomeric purity of *p*-*R*,*p*-*R*-**11** was 75%. Following isolation, the binaphtholate complex *S*,*p*-*S*,*p*-*S*-**12** was converted into a dimethyl analog, which was treated with hydrochloric acid to yield a red precipitate of titanocene dichloride *p*-*S*,*p*-*S*-**11**.

**Scheme 5:**
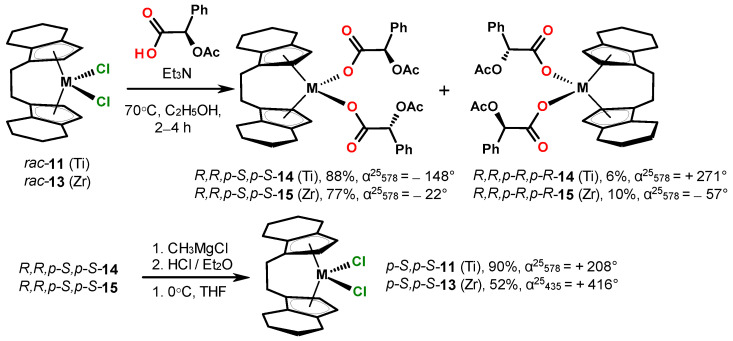
Resolution of *bis*-tetrahydroindenyl Ti and Zr complexes with *R*-acetylmandelic acid.

Optically active acids present an alternative to enantiomerically pure binaphthols for the resolution of chiral *ansa*-metallocenes [47]. The corresponding carboxylate complexes were synthesized through various methods: (i) converting metallocene dichloride complexes into dimethyl analogs followed by their reaction with optically active carboxylic acids; (ii) reacting metallocene dichlorides with lithium or sodium salts of acids; and (iii) treating metallocene dichlorides with acids in the presence of triethylamine. It was demonstrated that metallocenes react almost completely with optically active acids, such as *R*-acetylmandelic acid in the presence of Et_3_N (Scheme 5), yielding diastereomeric carboxylate complexes **14** and **15**. These complexes can be efficiently separated by fractional crystallization, achieving yields of 77–88% and minimizing the loss of the desired diastereomerically pure complexes. The conversion of carboxylates back to dichloride complexes *p*-*S*,*p*-*S*-**11** and *p*-*S*,*p*-*S*-**13** was accomplished via dimethyl derivatives followed by treatment with hydrochloric acid in ether (yield > 50%).

The derivatization method for *ansa*-complexes described above remains the most widely utilized approach. Subsequent research has focused on refining this method to increase the yield and enantiomeric purity of the target metal complexes.

**Scheme 6:**
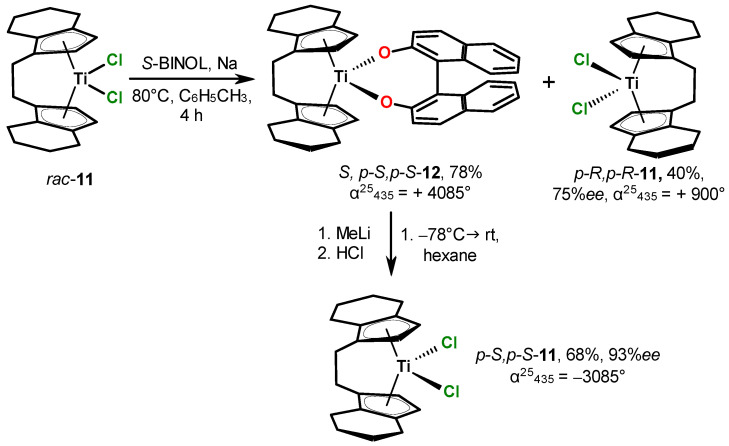
Derivatization of Ti complex *rac*-**11** using *S*-binaphthol.

To minimize the loss of enantiomers of complex **11**, the racemic mixture was subjected to reflux with sodium and *S*-binaphthol (Scheme 6) [48,49]. This process increased the yield of enantiomerically pure complex *S*,*p*-*S*,*p*-*S*-**12** to 78%, while dichloride *p*-*R*,*p*-*R*-**11** was isolated at a yield of 40%. Following treatment of *S*,*p*-*S*,*p*-*S*-**12** with a solution of MeLi in hexane and subsequently HCl in ether, *p*-*S*,*p*-*S*-**11** was obtained with a yield of 68% and an enantiomeric purity of 93%, as confirmed by ^1^H NMR spectroscopy of the carboxylate complex *R*,*R*,*p*-*S*,*p*-*S*-**14**.

**Scheme 7:**
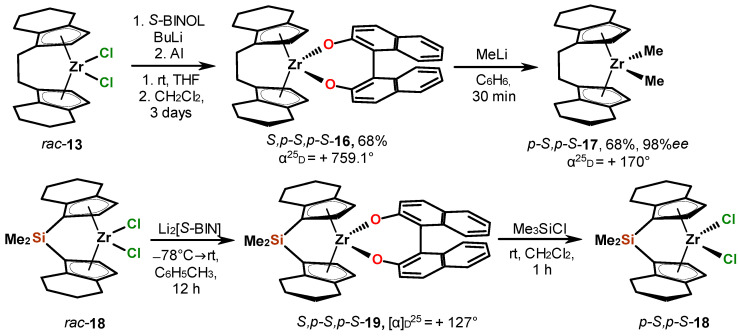
Synthesis of enantiomerically pure Zr complexes *p*-*S*,*p*-*S*-**17** and *p*-*S*,*p*-*S*-**18**.

The enantiomerically pure Zr complex *p*-*S*,*p*-*S*-**17** was synthesized through several stages, beginning with racemic **13**, which underwent a reaction with dilithium binaphtholate, followed by treatment with aluminum powder in dichloromethane (Scheme 7), resulting in the formation of complex *S*,*p*-*S*,*p*-*S*-**16**. Further alkylation of *S*,*p*-*S*,*p*-*S*-**16** with MeLi yielded the dimethyl complex *p*-*S*,*p*-*S*-**17** with a yield of 63% and an enantiomeric purity of 98%*ee* [50]. This approach also led to the synthesis of *p*-*S*,*p*-*S*-**18** with a yield of 14% [51].

**Scheme 8:**
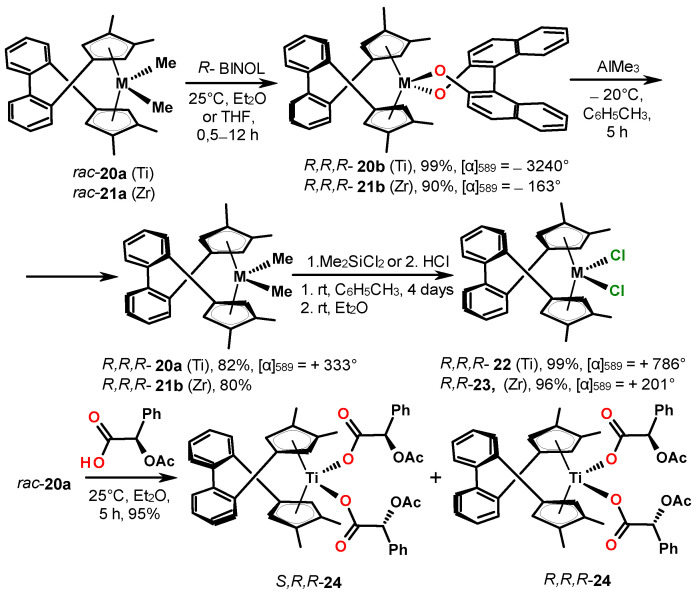
Resolution of racemic Ti (**20a**) and Zr (**21a**) complexes with *R*-binaphtholate and acetylmandelic acid.

The resolution of racemic *ansa*-cyclopentadienyl Ti (**20a**) and Zr (**21a**) complexes containing biphenyl bridging groups was successfully achieved using *R*-binaphtholate, while acetylmandelic acid was successfully applied only to *ansa*-L_2_TiCl_2_ (Scheme 8) [52,53]. This process resulted in the acquisition of enantiomerically pure complexes *R*,*R*,*R*-**20b** and *R*,*R*,*R*-**21b**. The reaction of titanocene **20a** with *R*-acetylmandelic acid produced a mixture of diastereomers **24** (the yield was determined by ^1^H NMR of the reaction mixture), which could not be separated via column chromatography.

**Scheme 9:**
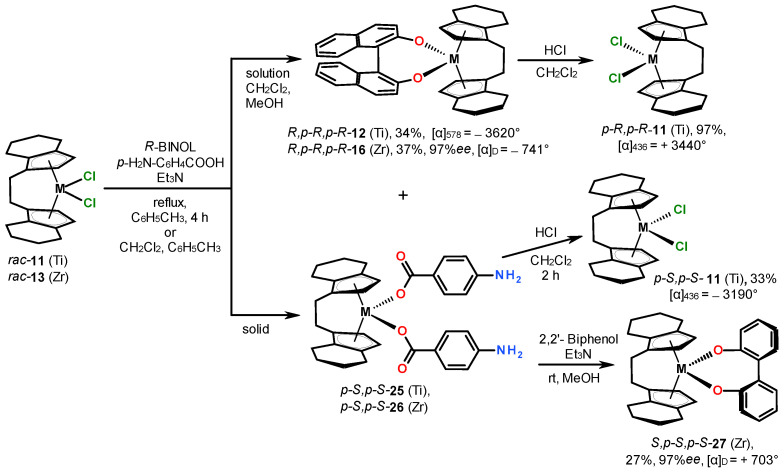
Derivatization of complexes *rac*-**11** (Ti) and *rac-***13** (Zr) with a mixture of *R*-binaphthol and *p*-aminobenzoic acid.

Complexes *rac*-**11** (Ti) and *rac*-**13** (Zr) were derivatized using a mixture of *R*-binaphthol and *p*-aminobenzoic acid in toluene under reflux with an excess of triethylamine (Scheme 9) [54,55]. This reaction yielded binaphtholate complexes *R*,*p*-*R*,*p*-*R*-**12** (34%) and *R*,*p*-*R*,*p*-*R*-**16** (37%), along with aminobenzoic acid derivatives *p*-*S*,*p*-*S*-**25** and *p*-*S*,*p*-*S*-**26**, which were separated based on their differing solubilities in non-polar solvents. The titanocene binaphtholates were converted into enantiomerically pure dichloride complexes *p*-*R*,*p*-*R*-**11** (97%) and *p*-*S*,*p*-*S*-**11** (33%) through treatment with hydrochloric acid in dichloromethane, while the Zr *p*-aminobenzoate was transformed into biphenolate *S*,*p*-*S*,*p*-*S*-**27** (27%, 97%*ee*) via reaction with 2,2′-biphenol.

**Scheme 10:**
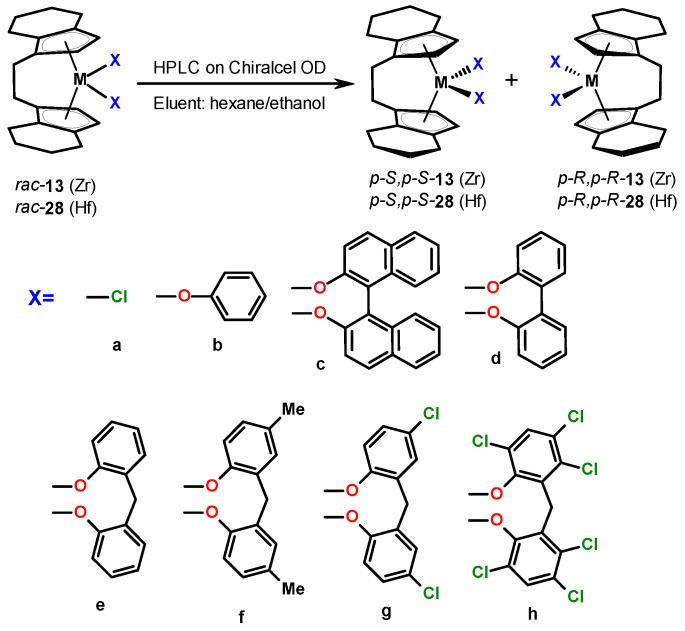
Separation of metallocenes *rac*-**13** (Zr) and *rac*-**28** (Hf) using chiral HPLC.

Bidentate phenolates of metallocenes *rac*-**13** (Zr) and *rac*-**28** (Hf) (Scheme 10) were quantitatively resolved into enantiomers using HPLC on cellulose (3,5-dimethylphenylcarbamate) as the chiral stationary phase, with a hexane/ethanol (9:1 *v*/*v*) mixture serving as the eluent [56]. The specific rotation exhibited a positive value for the first peak (*S*) and a negative value for the second (*R*). However, the dichlorides **13a** and **13b** were not separated into enantiomers under these conditions. The method was further extended by employing new commercially available bidentate ligands (c–h) for the derivatization of *rac*-**13** [57]. In all cases, the (+)-isomers were eluted first, with the highest separation factor observed for complex *rac*-**13d**. Optically active biphenolate hafnocenes *R*,*p*-*R*,*p*-*R*-**28d** (α^25^_435_ = −1100°) and *S*,*p*-*S*,*p*-*S*-**28d** (α^25^_435_ = +1100°) were also isolated.

**Scheme 11:**
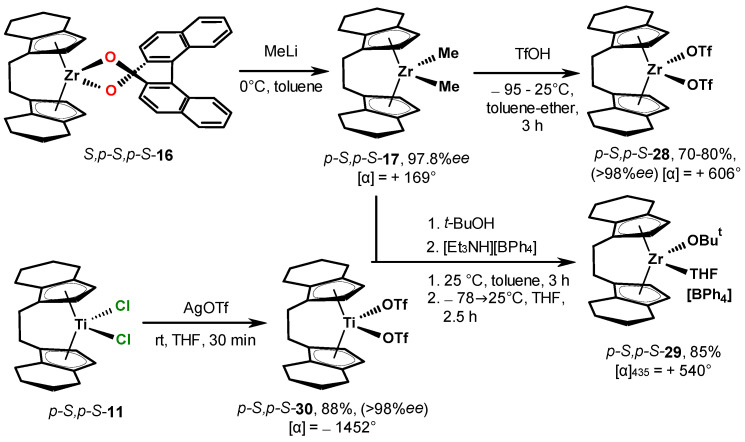
Synthesis of enantiomerically pure triflates of *ansa*-metallocenes Zr (**29**) and Ti (**30**).

Enantiomerically pure triflate derivatives of *ansa*-metallocenes Ti (**28**) and Zr (**30**) were synthesized starting from titanocene *p*-*S*,*p*-*S*-**11** and zirconocene binaphtholate *S*,*p*-*S*,*p*-*S*-**16**, respectively, as depicted in Scheme 11 [58,59,60]. Additionally, the cationic Zr compound *p*-*S*,*p*-*S*-**29** was obtained.

### 2.2. Path B: Stereoselective Synthesis of ansa-Metallocenes via Metalation of Chiral η^5^-Ligand Precursors

Optically active C_2_- and C_1_-symmetric *ansa*-complexes can be synthesized from enantiomerically pure *η*^5^-ligand precursors (path B). This method typically results in high diastereo- and enantioselectivity in the synthesis of metallocenes.

**Scheme 12:**
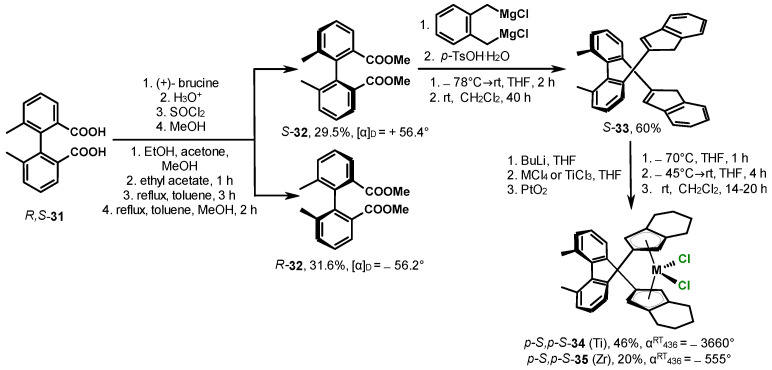
Synthesis of Ti (*p*-*S*,*p*-*S*-**34**) and Zr (*p*-*S*,*p*-*S*-**35**) complexes starting from enantiomerically pure ligands.

For example, optically active C_2_-symmetric titanium (*p*-*S*,*p*-*S*-**34**) and zirconium (*p*-*S*,*p*-*S*-**35**) complexes were synthesized via the metalation of ligand *S*-**33**, which features a biphenyl bridge, achieving yields of 46% and 20%, respectively (Scheme 12) [61]. The ligand was obtained by separating the dicarboxylic acid *R*,*S*-**31** into enantiomers *R*-**32** and *S*-**32** using (+)-brucine. The reaction of enantiomer *S*-**32** with a dimagnesium Grignard reagent, followed by protonolysis, yielded enantiomerically pure ligand *S*-**33** with a 60% yield.

**Scheme 13:**
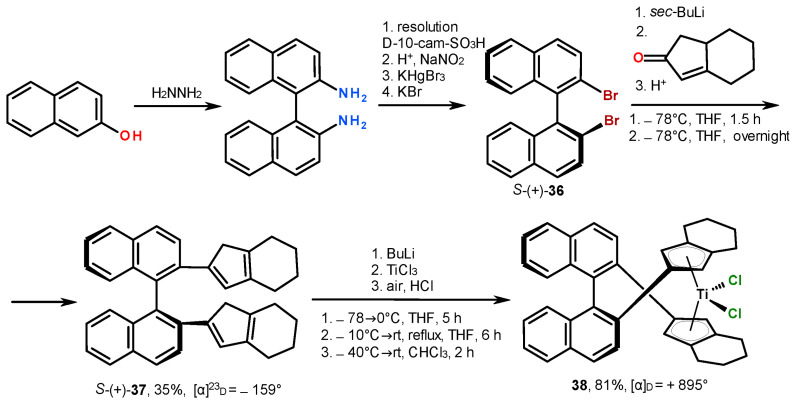
Synthesis of optically active titanocene **38**.

A similar approach was employed for the synthesis of a C_2_-symmetric optically active titanocene **38**, which contains a homotopic *bis*-tetrahydroindenyl ligand. This complex was synthesized from enantiomerically pure Br-substituted binaphthalene *S*-(+)-**36**, yielding only one stereoisomer of complex **38** with a yield of 68% (Scheme 13) [62]. The configuration of the binaphthyl fragment controls the structure of the complex, thereby excluding the formation of diastereomeric pairs during transmetalation. 

**Scheme 14:**
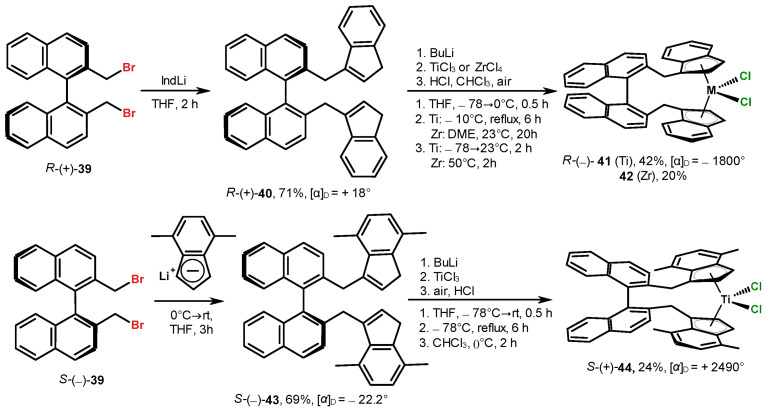
Synthesis of enantiomerically enriched Ti and Zr complexes from optically active Br-substituted binaphthyl precursors.

Halterman and co-workers proposed a method for the synthesis of enantiomerically enriched complexes **41**, **42**, and **44** (Scheme 14) based on optically active ligands obtained from Br-substituted binaphthyl structures **39** [62,63]. The ligand *R*-(+)-**39** was converted into (*R*)-(+)-2,2′-*bis*(1-indenylmethyl)-1,1′-binaphthyl (**40**), which upon coordination with titanium (**41**) and zirconium (**42**) formed only one C_2_-symmetric stereoisomer (Scheme 14). This method was later adapted for substituted indene [64]. For example, the reaction of 4,7-dimethylindenyllithium with *S*-(−)-**39** produced the ligand *S*-(−)-**43** with a 69% yield. The subsequent reaction of the anion of ligand **43** with TiCl_3_ produced complex **44** with a 24% yield. The developed method provided hard-to-reach enantiomerically pure *bis*-indenyl complexes; in this process, the substitution of Ti with Zr or introducing methyl groups into the indenyl ligand significantly reduced the yield of the target metallocenes.

**Scheme 15:**
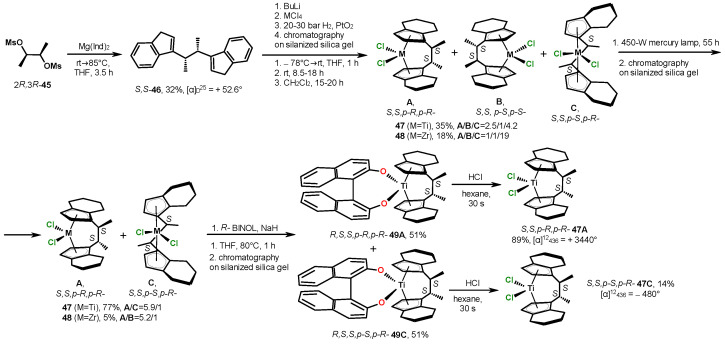
Synthesis and purification of metallocenes **47**–**49**.

Enantiomerically pure *bis*-indenyl ligand *S*,*S*-**46** was synthesized from the mesylate of (*R*,*R*)-2,3-butanediol **45** (Scheme 15) [65]. Deprotonation of *S*,*S*-**46** followed by transmetalation to a transition metal and hydrogenation resulted in a mixture of diastereomeric complexes, with the content of isomer **C** increasing when Ti was replaced with Zr. The optically active Ti complexes **47** were subjected to photolysis, after which the mixture reacted with *R*-(+)-binaphthol and was separated on silanized silica gel. This process yielded enantiomerically pure titanocenes *p*-*R*,*p*-*R*-**47A** (45%) and *p*-*R*,*p*-*S*-**47C** (14%). For zirconium, the yield of the target metallocenes was significantly lower, at 9%, for the isomers *p*-*R*,*p*-*R*-**48A** and *p*-*S*,*p*-*S*-**48C**, with a ratio of 5.9:1.

**Scheme 16:**
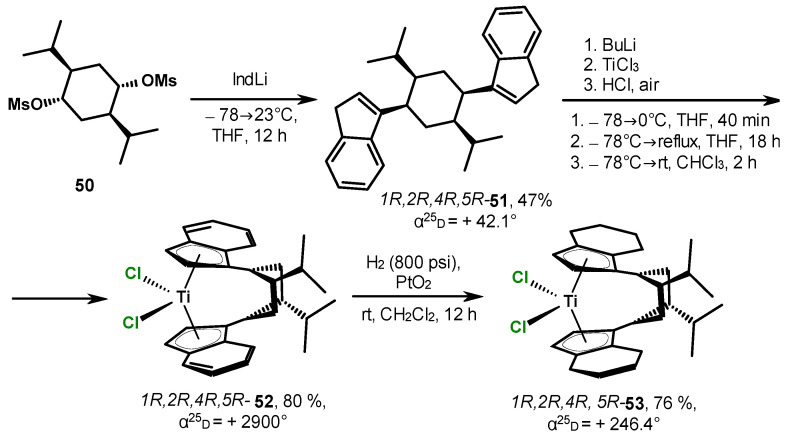
Synthesis of enantiomerically pure titanocene **52**.

The reaction of the *bis*(methanesulfonate) ester of 2,4-diisopropyl-1,4-cyclohexanediol (**50**) with IndLi in THF produced ligand (+)-(*1R*,*2R*,*4R*,*5R*)-**51**, which upon metalation with BuLi and TiCl_3_, followed by oxidative workup (HCl, air, chloroform), selectively yielded enantiomerically pure titanocene **52** with a high yield (Scheme 16) [66]. The corresponding *bis*(indenyl)zirconium dichloride was not obtained from this route. Complex **52** was hydrogenated over PtO_2_ to give *1R*,*2R*,*4R*,*5R*-**53** in good yield.

**Scheme 17:**
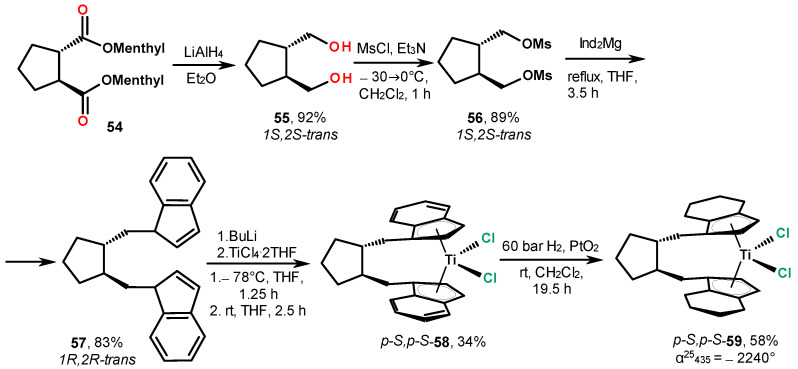
Stereoselective synthesis of titanocene complexes *p*-*S*,*p*-*S*-**58** and *p*-*S*,*p*-*S*-**59**.

High stereoselectivity in the synthesis of titanocene complex *p*-*S*,*p*-*S*-**58** was achieved when the optically active ligand (*1R*,*2R*)-*trans*-1,2-*bis*-(1-indenylmethyl)cyclopentane **55**, obtained from *di*-(*1R*,*2S*,*5R*)-menthyl-(*1S*,*2S*)-*trans*-1,2-cyclopentanedicarboxyl **54**, was reacted with BuLi and TiCl_4_·2THF (Scheme 17) [67]. Subsequent hydrogenation of *p*-*S*,*p*-*S*-**58** yielded *p*-*S*,*p*-*S*-**59**.

**Scheme 18:**
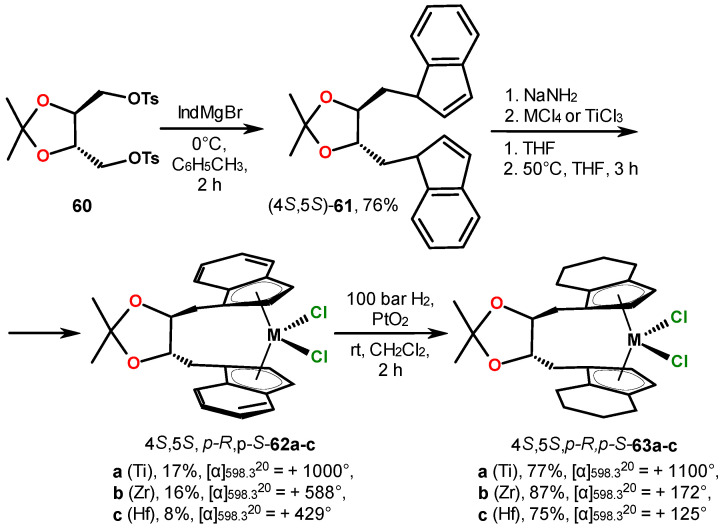
Synthesis of optically active *meso*-isomers of complexes **62a**–**c** and **63a**–**c**.

Using the less conformationally rigid C_4_-bridged (*4S*,*5S*)-*trans*-4,5-*bis*(1H-inden-3-ylmethyl)-2,2-dimethyl-1,3-dioxolane ligand **60**, obtained from enantiomerically pure tosylate **59** as a chiral substrate, resulted in a loss of racemo-selectivity and the formation of optically active *meso*-isomers of complexes **62a**–**c** and **63a**–**c** (Scheme 18) [68].

**Scheme 19:**
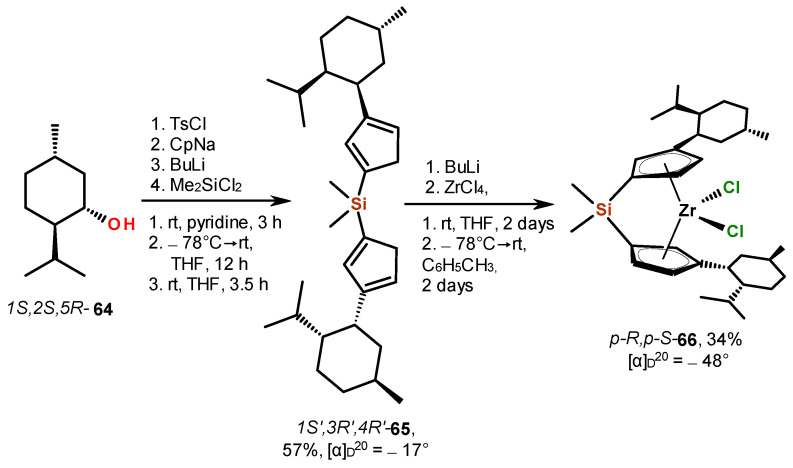
Synthesis of optically active Zr complex *meso*-**66**.

**Scheme 20:**
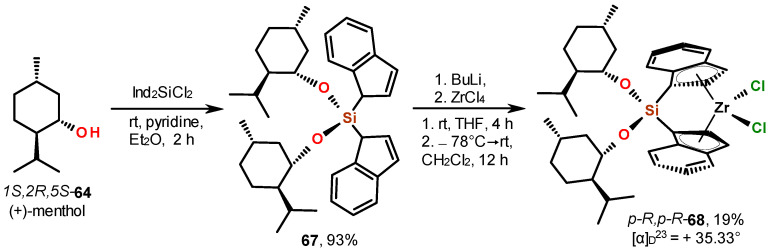
Stereoselective synthesis of Zr complex *p*-*R*,*p*-*R*-**68**.

The formation of the *meso*-isomer (**66**) was also observed in the case of (−)-dimethyl-*bis*-[3-(neomenthyl)cyclopentadienyl]silane **65**, derived from neomenthol *1S*,*2S*,*5R*-**64**. In the ^1^H NMR spectra of the reaction mixture, signals for three possible isomers were observed with a ratio of 5:2:3. However, after recrystallization from pentane, only one optically active metallocene *meso*-**66** was isolated with a yield of up to 34% (Scheme 19) [69].

(+)-(*1S*,*2R*,*5S*)-Menthol (*1S*,*2R*,*5S*-**64**) was introduced as a chiral element in the bridging group of the *bis*-indenyl ligand, leading to the synthesis of enantiomerically pure di[(*1*′*S*,*2*′*R*,*5*′*S*)-menthoxy]silylene-*bis*[*η*^5^-1-(*R*,*R*)-(+)-indenyl] zirconium dichloride (*p*-*R*,*p*-*R*-**68**) with a yield of up to 19% (Scheme 20) [70].

**Scheme 21:**
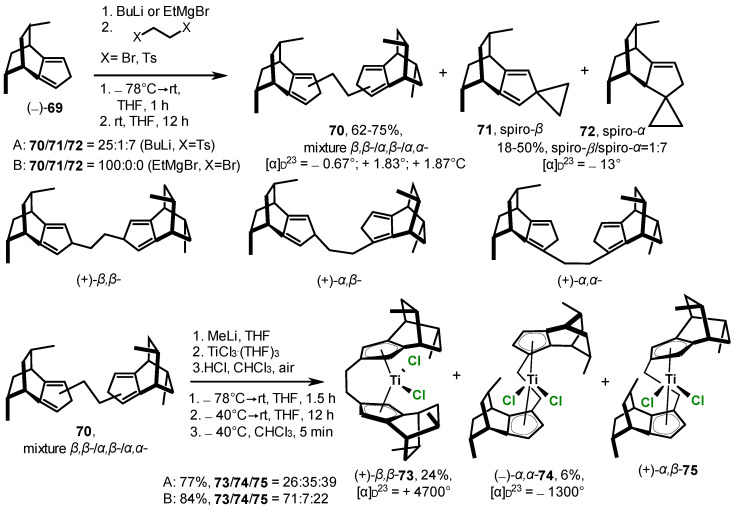
Polycyclic compounds as precursors in the synthesis of chiral titanocenes **73**–**75**.

Various isomers of ethylene-bridged cyclopentadienyl titanium complexes **73**–**75** were synthesized from the sterically hindered enantiomerically pure (−)-8,10-dimethyltricyclo[5.2.2.0^2^,^6^]-2,5-undecadiene **69** (Scheme 21) [71]. It was observed that similar complexes with silicon or methylene bridges could not be synthesized. The metalation stage of ligand **70** occurred with varying selectivity, as this precursor represented a difficult-to-separate mixture of isomers differing by the position of the bridge. Consequently, complexes **73**–**75** were obtained with yields of up to 84%, with a ratio of **73**/**74**/**75** = 71:7:22.

**Scheme 22:**
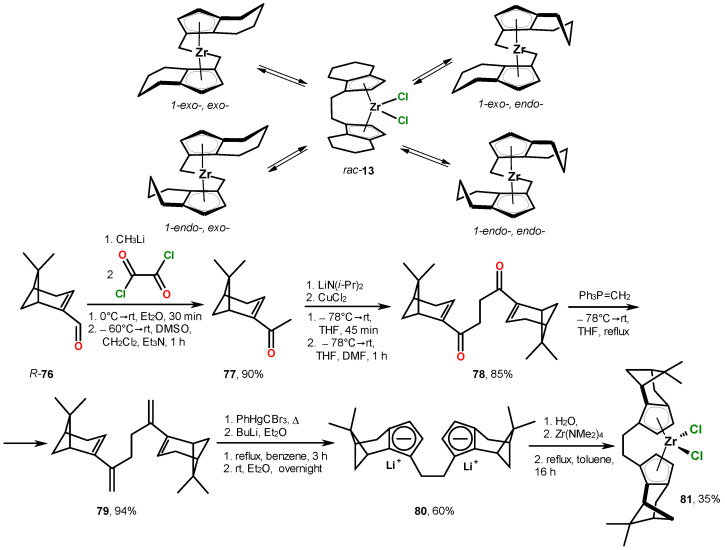
Stabilization of ligand conformation as a significant factor in the stereoselective synthesis of chiral complexes.

To restrict the conformational mobility of the cyclohexane fragment of the tetrahydroindenyl ligand and to stabilize the *exo*-, *exo*-configuration (Scheme 22), ligand **80** based on (*R*)-myrtenal **76** was synthesized. Transmetalation of the ligand with Zr(NMe_2_)_4_ produced a single C_2_-symmetric stereoisomer of complex **81** with an *exo*-, *exo*-configuration of the cyclohexyl rings, achieving a yield of 35% [72].

**Scheme 23:**
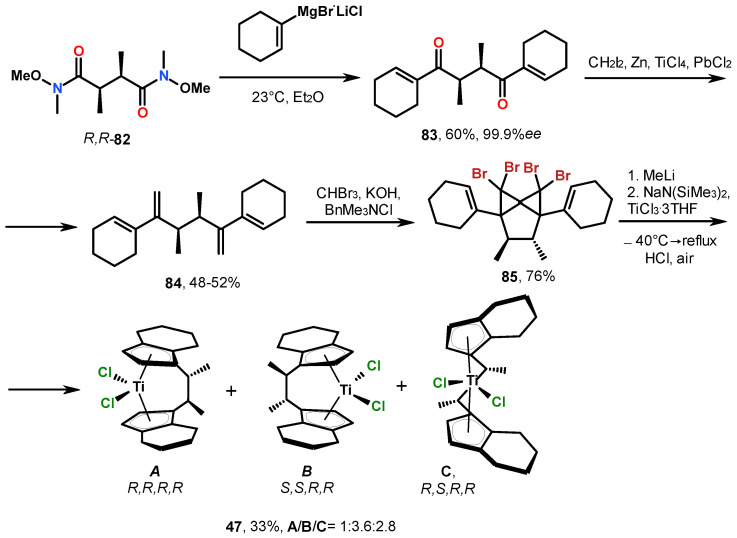
*Bis*-amide *R*,*R*-**82** in the synthesis of Ti complexes **47A**–**C**.

The reaction of optically active *bis*-amide *R*,*R*-**82** with cyclohexenylmagnesium bromide lithium chloride yielded the corresponding *bis*-enone **83** without loss of enantiomeric purity (>99.9%*ee*) (Scheme 23) [73]. Through the stages of methylenation of **83** and *bis*-dibromocyclopropanation of compound **84**, the ligand precursor **85** was obtained. The rearrangement into a chiral C_2_-symmetric ligand was performed using MeLi, followed by deprotonation with sodium *bis*(trimethylsilyl)amide and reaction with TiCl_3_·3THF without isolation. Three isomers—(*S*,*S*,*R*,*R*)-**47B**, the pseudoenantiomeric complex (*R*,*R*,*R*,*R*)-**47A**, and the pseudomeso-complex (*R*,*S*,*R*,*R*)-**47C**—were isolated in a ratio of **B**:**A**:**C** = 3.6:1:2.8. This method allows for the rapid synthesis of *bis*-tetrahydroindenyl complexes with good *rac*-selectivity from alkenyl Grignard reagents and *bis*-amides.

Another strategy for obtaining optically active *ansa*-metallocenes via Path B involves the synthesis of complexes with nonequivalent ligands (C_1_-symmetric complexes). In this scenario, the chirality of the complex is introduced by incorporating a substituent with stereogenic centers into one of the *η*^5^-ligands.

**Scheme 24:**
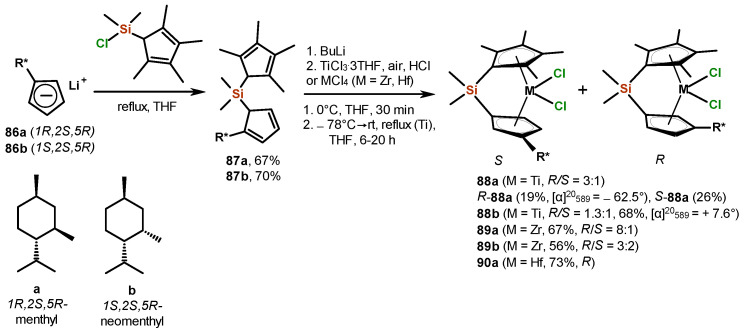
Effect of menthyl and neomenthyl substituent in Cp-ring on stereoselectivity of metal complex synthesis.

For example, a method for synthesizing optically active *ansa*-metallocenes **88**–**90a**,**b** from Si-bridged *bis*-cyclopentadienyl ligands **86a** and **86b**, where one of the Cp fragments contains an enantiomerically pure menthyl or neomenthyl substituent, was developed (Scheme 24) [74,75]. The reaction with TiCl_3_ yielded a mixture of diastereomers *R*- and *S*-**88a** with a ratio of 3:1. The titanium complex *R*-**88a** was crystallized from hot hexane, while the *S*-enantiomer **88a** was obtained after UV irradiation of a mixture of complexes in toluene, followed by passing the solution through silylated silica gel and crystallization from hot pentane. Zr (**89a**,**b**) and Hf (**90b**) complexes were synthesized similarly. The content of the *R*-enantiomers of *ansa*-metallocenes increased with the growing atomic radius of the transition metal when using the menthyl-substituted ligand **86a**. In all cases, replacing the menthyl group with a neomenthyl group led to a loss of diastereoselectivity.

**Scheme 25:**
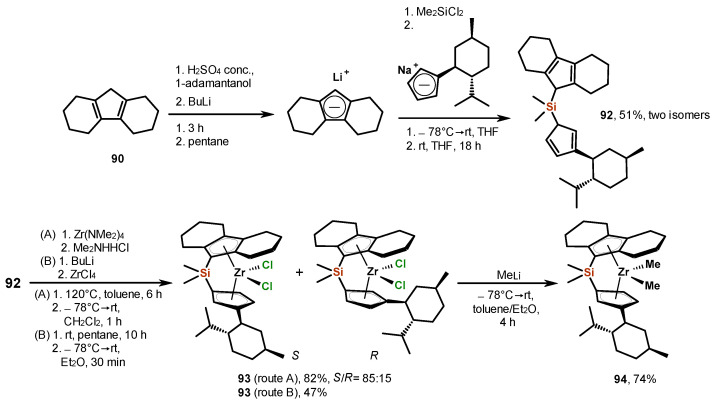
Synthesis of mixed menthyl-substituted cyclopentadienyl-octahydrofluorenyl *ansa*-complexes **93**, **94**.

Following a similar approach to *bis*-cyclopentadienyl complexes, optically active mixed menthyl-substituted cyclopentadienyl-octahydrofluorenyl *ansa*-complexes **93** and **94** were synthesized (Scheme 25) [76]. For this purpose, ligand **92** was metalated via reaction with Zr(NMe_2_)_4_ (route A, Scheme 25) in toluene at 120 °C. Subsequent treatment with Me_2_NH·HCl produced a mixture of diastereomers of complex **93** with a yield of 82% and a ratio *S*/*R* = 85:15. Deprotonation of **92** with butyllithium (route B, Scheme 25) and transmetalation with ZrCl_4_ yielded stereoisomers of complex **93** with a low yield.

**Scheme 26:**
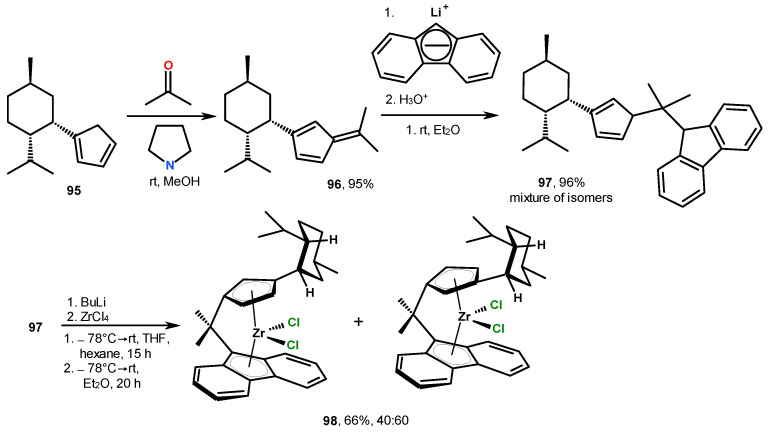
Synthesis of diastereomeric complexes **98**.

Neomenthyl-substituted *ansa*-complexes with an isopropylidene bridge (**98**) were synthesized (Scheme 26) [77]. In the initial stages of synthesis, the menthyl-substituted fulvene **96** reacted with lithium fluorenide to yield precursor **97**. The metalation of ligand **97** resulted in a mixture of diastereomeric complexes **98** in a ratio of 60:40. Thus, introducing the neomenthyl group and isopropylidene bridge into the ligand decreased the stereoselectivity of complex synthesis.

**Scheme 27:**
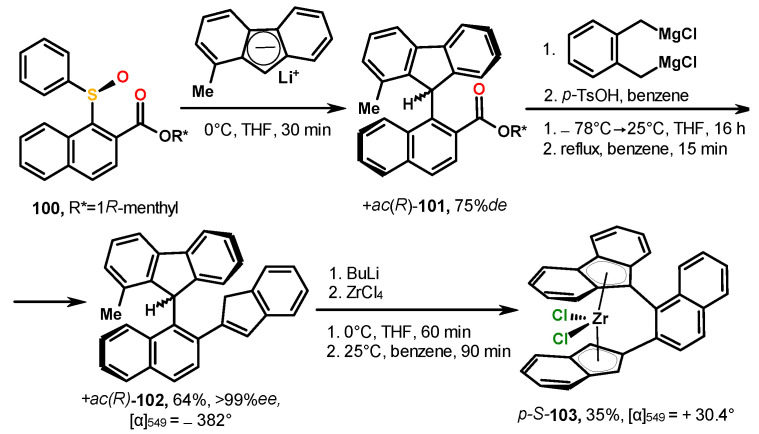
Synthesis of indenyl-fluorenyl *ansa*-zirconocene with a naphthyl bridge *p*-*S*-**103**.

High diastereoselectivity was achieved in the synthesis of the enantiomerically pure (>99%*ee*) indenyl-fluorenyl *ansa*-zirconocene with a naphthyl bridge *p*-*S*-**103** (Scheme 27) [78]. The ligand **102** used in this synthesis was obtained by the reaction of (*1R*)-menthyl-(*S*)-1-(*p*-tolylsulfinyl)naphthalene-2-carboxylate (**100**) with 1-methylfluorenyllithium at 0 °C. The resulting ester reacted with a Grignard dimagnesium reagent to afford the *R*-enantiomer of the *ansa*-ligand **102**, which upon metalation selectively produced complex *p*-*S*-**103**.

**Scheme 28:**
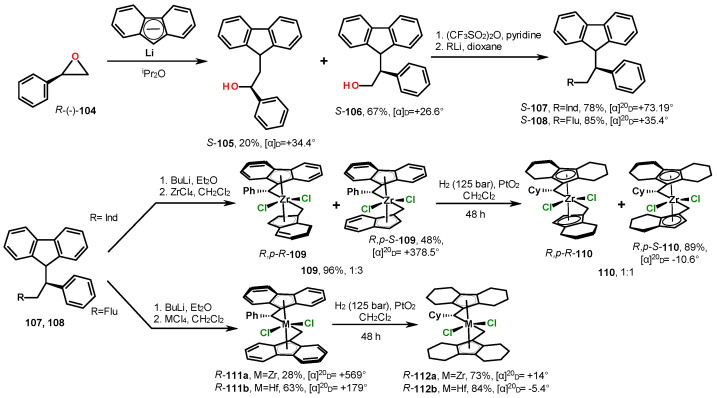
Synthesis of complexes **109**–**112**.

The reaction of *R*-(−)-epoxystyrene **104** with fluorenyllithium and subsequent transformations through various stages provided enantiomerically pure *ansa*-ligands *S*-**107** and *S*-**108** with a stereogenic center in the bridge (Scheme 28) [79,80]. Deprotonation of *S*-**107** with ZrCl_4_ yielded diastereomers *R*,*p*-*R*-**109** and *R*,*p*-*S*-**109** in a 1:3 ratio. Hydrogenation of **109** with H_2_/PtO_2_ produced corresponding *R*,*p*-*R*-**110** and *R*,*p*-*S*-**110** in a 1:1 ratio. The metalation of *bis*-fluorenyl ligand **108** afforded Zr and Hf complexes *R*-**111a**,**b** with yields of 28% and 63%, respectively. Octahydrofluorenyl complexes *R*-**112a**,**b** were obtained following the hydrogenation of *R*-**111a**,**b**.

**Scheme 29:**
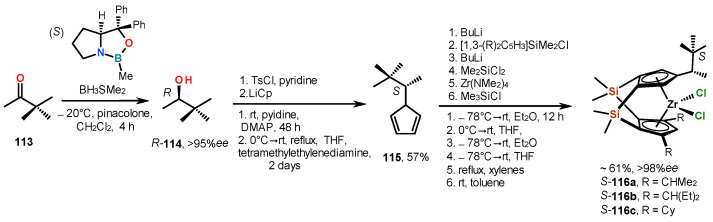
Synthesis of enantiomerically pure C_1_-symmetric complexes *S*-**116a**–**c**.

Enantiomerically pure C_1_-symmetric *ansa*-complexes *S*-**116a**–**c** were synthesized from optically active cyclopentadiene with a branched hydrocarbon substituent (**115**) (Scheme 29) [81]. To enhance the rigidity of the complex molecule, the ligands were linked by two Me_2_Si-bridges.

**Scheme 30:**
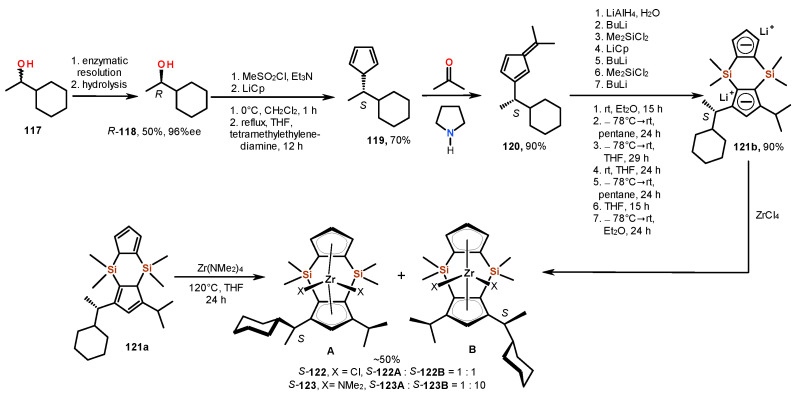
Effect of σ-ligand on the diastereoselectivity in the synthesis of complexes **122** and **123**.

The presence of two different substituents in one of the cyclopentadienyl fragments of ligand **121** led to the formation of two isomers of complexes *S*-**122** in a 1:1 ratio when using ZrCl_4_ (Scheme 30) [81]. Utilizing Zr(NMe_2_)_4_ as a metalation agent significantly increased the content of one of the possible diastereomers of complex *S*-**123**.

**Scheme 31:**
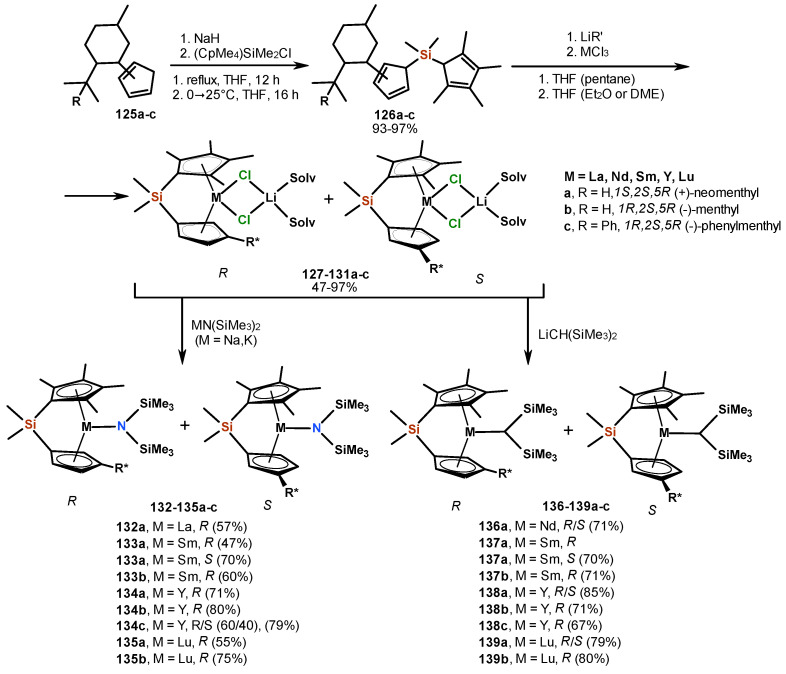
Preparation of enantiomerically pure *ansa*-complexes of group IIIB metals **127**–**139**.

The literature also provides examples of preparing enantiomerically pure complexes of group IIIB metals [82]. The synthetic approaches to chiral organolanthanide *ansa*-*η*^5^-complexes have been discussed, with most work focusing on the synthesis of C_1_-symmetric compounds with stereogenic substituents in one of the cyclopentadienyl rings (Table 1) [83,84]. A series of enantiomerically pure La, Nd, Sm, Y, and Lu complexes **127**–**131a**–**c** were obtained via metalation of optically active cyclopentadienyl ligands with neomenthyl, menthyl, and phenylmenthyl substituents **124a**–**c** (Scheme 31). The study also demonstrated the potential for the epimerization of chloro lanthanide complexes to enhance the yield and isolation of their diastereomeric antipodes. It was shown that the degree of epimerization depends on the nature of the donor solvent, the lanthanoid ion, the structure of the substituent R*, and the temperature (Table 1). The epimerization of chloride complexes **127**–**131a**–**c** can be completely inhibited by adding ligands like TMEDA or 12-crown-4. To obtain stable lanthanide complexes effective in asymmetric catalysis, which do not undergo epimerization in solution, complexes **127**–**131a**–**c** were converted into amides **132**–**135a**–**c** and alkyls **136**–**139a**–**c** (Scheme 31).

**Scheme 32:**
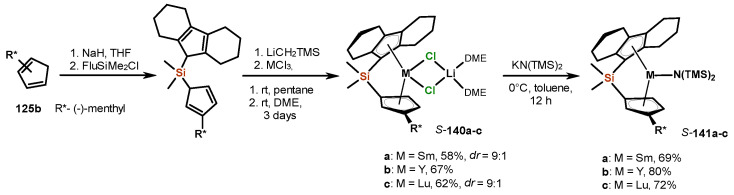
Synthesis of chiral C_1_-symmetric organolanthanide complexes *S*-**140a**–**c** and *S*-**141a**–**c**.

Chiral C_1_-symmetric organolanthanide complexes *S*-**140a**–**c** (M = Sm, Y, Lu), with *ansa*-bridged *η*^5^-octahydrofluorenyl and cyclopentadienyl ligands substituted with a menthyl group, were synthesized analogously to complexes **127**–**131a**–**c** (Scheme 32) [85]. The highest diastereoselectivity, reaching a ratio of 9:1, was attained when the reaction was performed in dimethoxyethane (DME). The reaction of the diastereomerically enriched complexes *S*-**140a**–**c** with KN(TMS)_2_ proceeded to furnish *S*-**141a**–**c** in quantitative yield. Remarkably, at ambient temperature, the process preserved the diastereomeric ratio almost entirely, mirroring that of the initial chloro complexes. The resulting amido complexes exhibited notable thermal stability and were isolated via extraction with boiling hexane. A single recrystallization from hot hexane afforded exclusively the diastereomerically pure amido complexes *S*-**141a**–**c**.

**Scheme 33:**
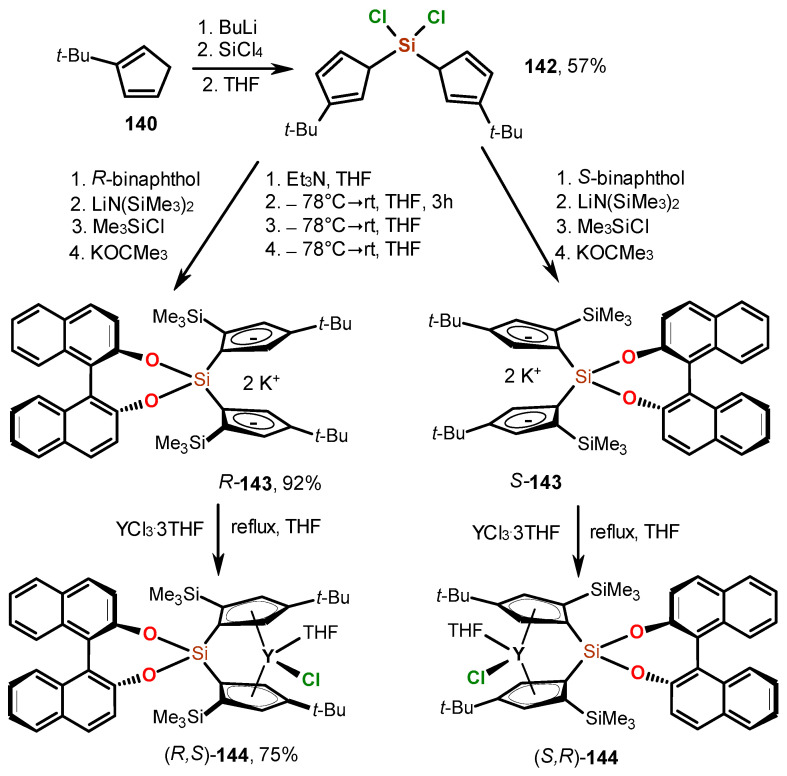
Application of enantiomerically pure binaphthol-bridged ligands in the synthesis of *ansa*-yttrocenes.

Bercaw and colleagues [86] synthesized enantiomerically pure C_2_-symmetric yttrocenes (*R*,*S*)-**144**, characterized by stereogenic centers within the bridging moiety. This was accomplished through the preliminary preparation of enantiomerically pure *ansa*-ligands **143** via the reaction of *R*- or *S*-binaphthol with *tert*-butyl-substituted *bis*(cyclopentadienyl)chlorosilane (**142**) (Scheme 33). Consequently, coordination to yttrium proceeded with complete diastereoselectivity: the ligand derived from *R*-binaphthol yielded (*R*,*S*)-**144**, whereas the ligand obtained from *S*-binaphthol directed the reaction exclusively toward the formation of (*S*,*R*)-**144**.

### 2.3. Path C: Stereoselective Synthesis of ansa-Metallocenes via Enantiomerically Pure σ-Complexes

**Scheme 34:**
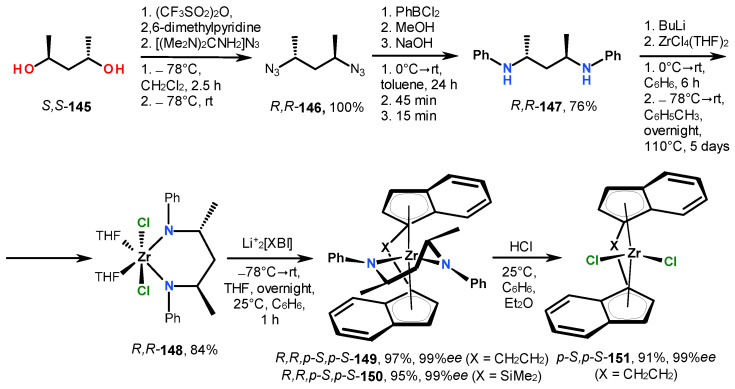
*R*,*R*-enantiomer of Zr amide as a precursor in the synthesis of enantiomerically pure *ansa*-complex *p*-*S*,*p*-*S*-**151**.

Enantiomerically pure σ-complexes can serve as effective stereodifferentiating precursors. For example, the synthesis of the *R*,*R*-enantiomer of amide complex **148**, which achieved a yield of 84%, was carried out starting from *R*,*R*-diphenylpropanediamine (**147**) (Scheme 34) [87]. Subsequent reactions of amide **148** with lithium salts of *ansa*-ligands containing ethanediyl or Me_2_Si bridges provided enantiomerically pure amide complexes **149** and **150** with yields of 97% and 95%, respectively. Compound **149** was transformed into the *bis*-indenyl complex *p*-*S*,*p*-*S*-**151**, attaining a yield of 91% and enantioselectivity exceeding 99%*ee*.

**Scheme 35:**
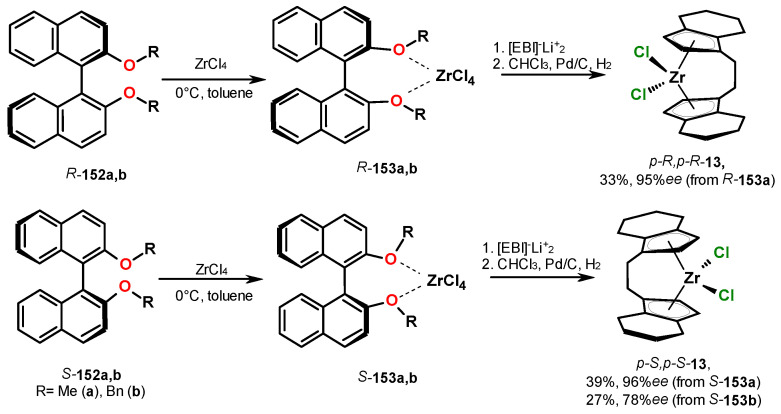
Enantioselective synthesis of Zr complex **13** via the association of ZrCl_4_ with enantiomerically pure *S*- or *R*-binaphthol ethers.

The diastereo- and enantioselective synthesis of the ethylene-*bis*-tetrahydroindenyl zirconium complex **13** was proposed via the association of ZrCl_4_ (*R*- or *S*-**153**) with enantiomerically pure sterically hindered *S*- or *R*-binaphthol ethers (Scheme 35) [88]. A higher enantioselectivity (95–96%*ee*) was observed in the reaction of binaphthol dimethyl ether complexes with the *ansa*-ligand dianion.

The growing interest in enantiomerically pure metallocenes is primarily motivated by their potential application as catalysts in various asymmetric syntheses. Another significant aspect is the investigation of stereoinduction and chiral recognition mechanisms, which facilitate the understanding of the structural features and dynamics of catalytically active centers, as well as the processes of substrate coordination and transformation by metal centers. In this regard, the subsequent chapters of this review will explore the applications of enantiomerically pure group IVB and IIIB transition metal *ansa*-*η*^5^-complexes, along with some mechanistic aspects of their asymmetric action across various types of reactions.

## 3. Application of Enantiomerically Pure *ansa*-*η*^5^-Complexes in Asymmetric Catalysis

### 3.1. Carbomagnesation of Unsaturated Compounds

The complexes of group IVB transition metals are widely acknowledged as effective catalysts for the formation of metal–carbon, carbon–carbon, and carbon–hydrogen bonds. The synthesis of Brintzinger’s *ansa*-titanocene and zirconocene complexes subsequently facilitated the development of highly active and stereoselective catalytic systems for alkene polymerization [3,4,5,6,7,8,9,10,11,89,90,91,92,93,94,95,96,97,98,99,100,101,102]. These complexes are also well known for their capability to catalyze di- and oligomerization reactions, as well as carbometalation and cyclometalation of alkenes and acetylenes [12,13,14,15,16,17,18,19,20,21,24,25,26,27,28,29,30,42,103,104,105,106,107,108]. A pivotal step in these reactions is the carbometalation of the unsaturated substrate, which generates a highly reactive metal–carbon bond. Consequently, the introduction of enantioselective catalysts into these reactions has enabled the development of efficient methodologies for the functionalization of alkenes, dienes, and acetylenes, yielding a wide range of optically active synthons that are in demand in organic synthesis [24,25,26,27,42,107,108,109,110,111,112].

**Scheme 36:**
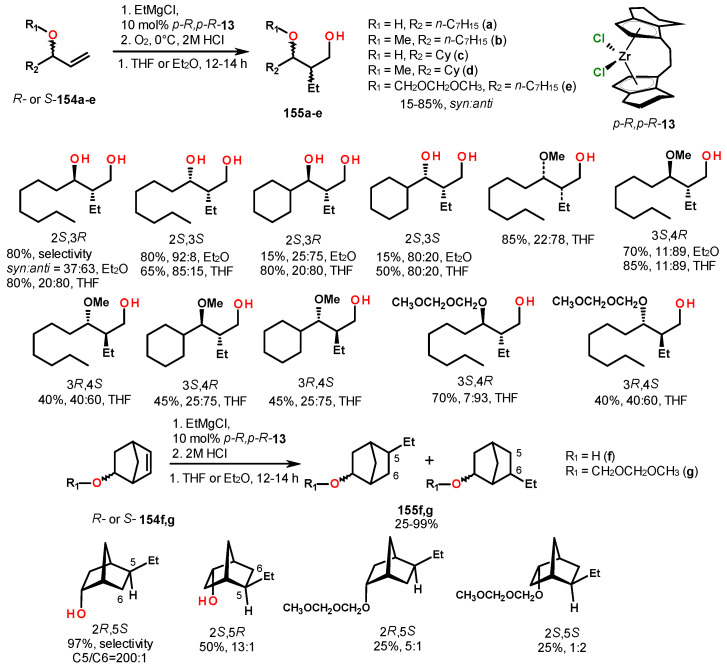
Complex *p*-*R*,*p*-*R*-**13** as a stereoselective catalyst for alkene carbomagnesiation.

The first report detailing the application of the enantiomerically pure zirconocene complex *p*-*R*,*p*-*R*-**13** as a stereoselective catalyst for the carbomagnesiation of alkenes was presented in 1993 [113]. Ethylmagnesiation of the allylic *R*- and *S*-enantiomers of alcohols **154a**,**c** and esters **154b**,**d**,**e** (Scheme 36) exhibited varying diastereoselectivity contingent upon the type of starting enantiomer. It was demonstrated that high diastereoselectivity could be attained when employing *S*-enantiomers of allylic alcohols (*syn*:*anti* = 92:8) and *R*-enantiomers of esters (*syn*:*anti* = 7:93). The reaction with *R*-norbornene alcohol **154f** proceeded with high regioselectivity, favoring the formation of the *2R*,*5S*-enantiomer of the ethylmagnesiation product **155f**.

**Scheme 37:**
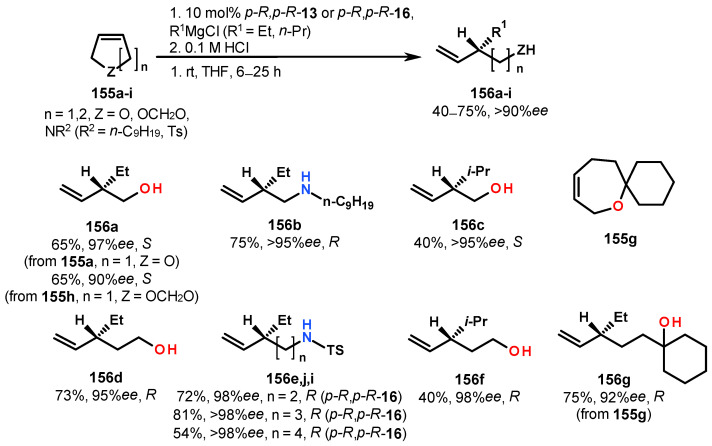
Carbomagnesation of heterocyclic alkenes catalyzed by enantiomerically pure complexes *p*-*R*,*p*-*R*-**13** and *R*,*p*-*R*,*p*-*R*-**16**.

In further developments of the method, the reaction of alkyl magnesium halides with readily available heterocyclic alkenes, catalyzed by the enantiomerically pure complexes *p*-*R*,*p*-*R*-**13** and *R*,*p*-*R*,*p*-*R*-**16**, was investigated (Scheme 37) [114,115]. Specifically, the reaction of cyclic olefins **156a**–**d** with EtMgCl or *n*-PrMgCl in the presence of *p*-*R*,*p*-*R*-**13** yielded carbomagnesation products **157a**–**g** with good yields (40–77%) and excellent enantioselectivity (over 90%*ee*). In the carbomagnesation reaction of cyclic amines **156b**,**e**,**j**,**i**, the carbometalation proceeded with product yields of 54–81% and enantioselectivity exceeding 95%*ee*.

**Scheme 38:**
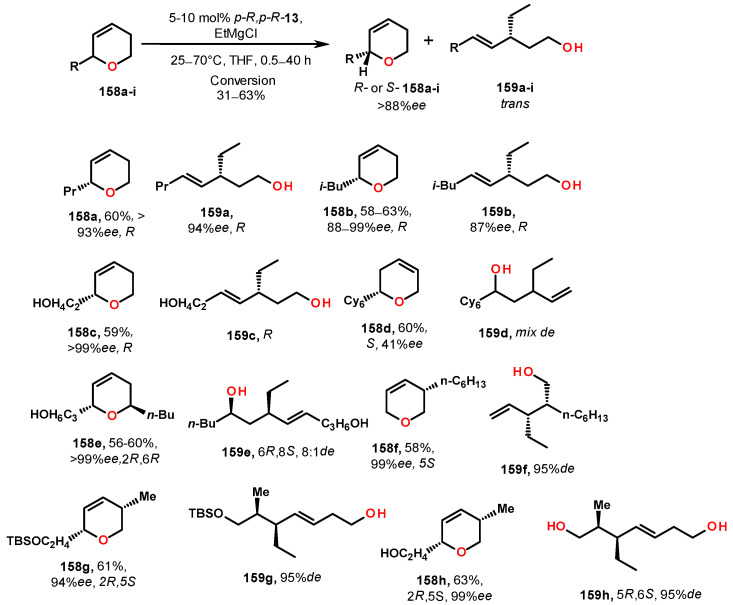
Carbomagnesation of *rac*-5,6-dihydropyrans, catalyzed with *p*-*R*,*p*-*R*-**13**.

Utilizing *rac*-5,6-dihydropyrans **158a**–**i** as substrates, carbomagnesation products **159a**–**i** were obtained with high enantiomeric purity. Moreover, enantiomerically enriched **158a**–**i** were isolated as a result of the kinetic resolution of the racemic mixture of the starting compounds, due to the participation of only the *S*-enantiomer in the reaction (Scheme 38) [115,116]. Thus, asymmetric catalytic carbomagnesation proves to be an effective method for the kinetic resolution of enantiomers of unsaturated pyrans, which are important synthons for medicinal chemistry [116].

**Scheme 39:**
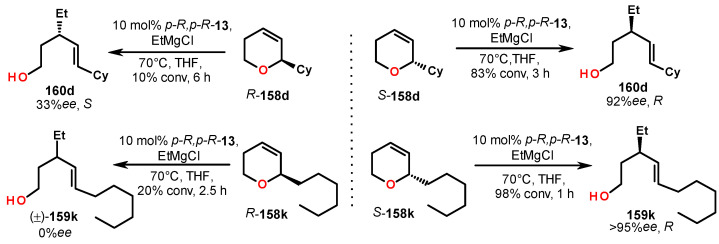
Kinetic effect of *p*-*R*,*p*-*R*-**13** in the ethylmagnesiation of pyrans.

To investigate the kinetic effect of *p*-*R*,*p*-*R*-**13** in the ethylmagnesiation reaction, enantiomerically pure substituted pyrans were employed. It was found that the reaction was most favorable for *S*-enantiomers of olefins (*S*-**158d**,**k**), which subsequently provided *R*-enantiomers of **160d**, **159k** with good yields and enantioselectivity (Scheme 39) [115].

**Scheme 40:**
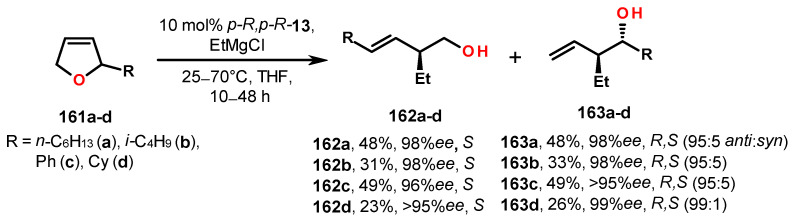
Kinetic resolution of dihydrofurans in the presence of *p*-*R*,*p*-*R*-**13**.

Substituted dihydrofurans **162a**–**d** also undergo kinetic resolution in the carbomagnesation reaction [117]. Unlike pyrans, both enantiomers of substituted furans fully participate in the reaction with the Grignard reagent (Scheme 40). In this case, the reaction proceeded with high regio-, enantio- (>98%*ee*), and diastereoselectivity (95%*de*) concerning both enantiomers of the substrates, resulting in the formation of different products: the *S*-isomer yielded only primary alcohol *S*-**162a**–**d**, while the *R*-enantiomer produced the secondary alcohol *R*,*S*-**163a**–**d**.

**Scheme 41:**
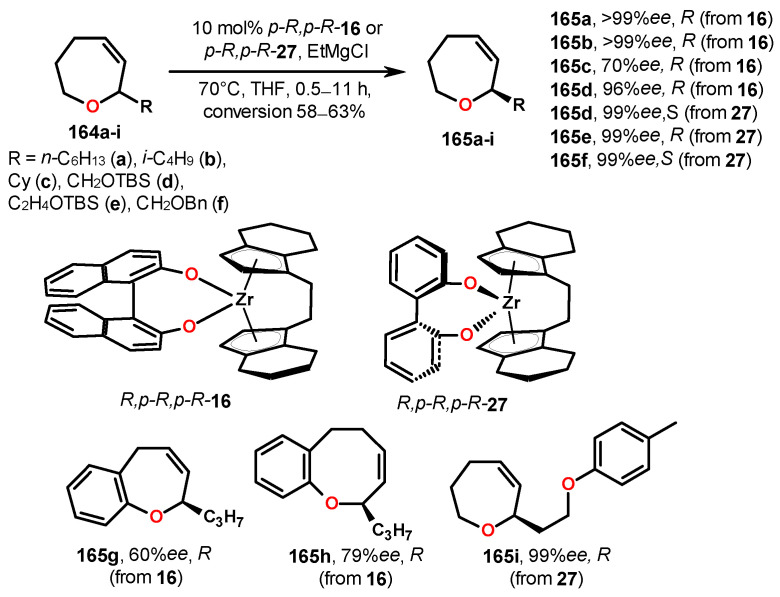
Ethylmagnesiation of seven-membered cycloolefins catalyzed by *p*-*R*,*p*-*R*-**16** or *R*,*p*-*R*,*p*-*R*-**27**.

The application of the binaphtholate complex *p*-*R*,*p*-*R*-**16** provided a high degree of enantiomer separation for seven-membered cycloolefins **164a**–**i**, predominantly resulting in the isolation of *R*-enantiomers **165** (Scheme 41) [115,118]. The use of the biphenol complex *p*-*R*,*p*-*R*-**27** yielded nearly identical results. A decrease in enantioselectivity to 70%*ee* was observed for the cyclohexyl-substituted substrate **165c**.

**Scheme 42:**
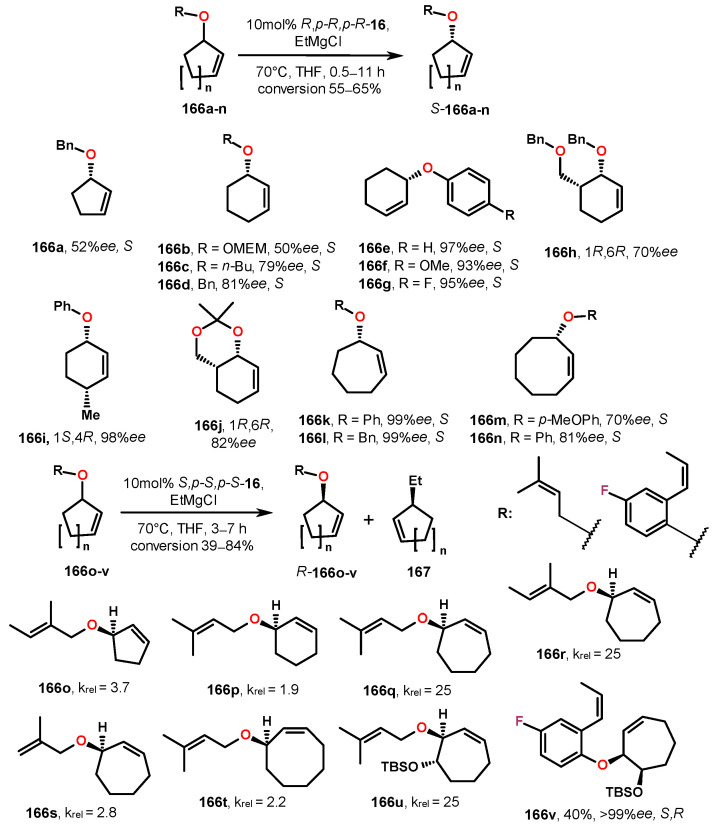
Carbomagnesiation of cyclic alkoxy-substituted olefins **166a**–**v** catalyzed by *R*,*p*-*R*,*p*-*R*-**16** or *S*,*p*-*S*,*p*-*S*-**16**.

During the carbomagnesiation reaction catalyzed by *R*,*p*-*R*,*p*-*R*-**16** or *S*,*p*-*S*,*p*-*S*-**16**, cyclic alkoxy-substituted olefins **166a**–**v** were also subjected to kinetic resolution, leading to the enrichment of the mixture with *S*-enantiomers exhibiting purities ranging from 50% to 99%*ee* (Scheme 42) [119,120,121]. In this case, the highest degree of separation was achieved in reactions involving seven-membered cyclic substrates **166k**,**l** and **166q**,**r**,**u**,**v**.

Thus, cyclic olefins in the presence of enantiomerically pure *ansa*-tetrahydroindenyl Zr complexes and Grignard reagents undergo kinetic resolution, during which one of the enantiomers can participate in an ethylmagnesiation reaction with ring opening. The structure of the starting olefins plays a significant role in these processes. For example, the enantioselectivity of the reaction is influenced by the size of the olefinic cycle (5-, 6-, 7-, or 8-membered) and the structure of the alkyl group in the R-O moiety. It is noted that increased steric hindrance of the substrate, both from the larger cycle and the substituent, leads to a loss of enantioselectivity in the process.

**Scheme 43:**
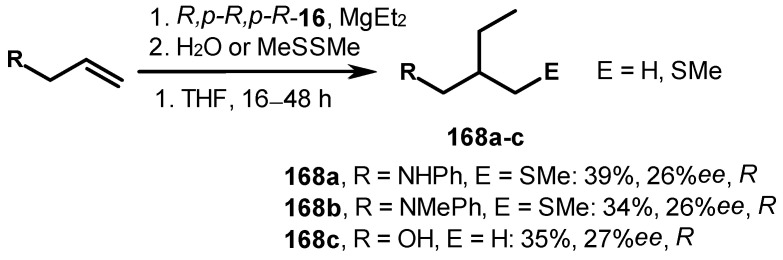
Carbomagnesiation of inactivated alkenes by MgEt_2_ in the presence of *R*,*p*-*R*,*p*-*R*-**16**.

The ethylmagnesiation of inactivated alkenes by MgEt_2_ in the presence of the rigid *ansa*-complex *R*,*p*-*R*,*p*-*R*-**16** proceeded with the formation of products **168a**–**c** with low yield and enantioselectivity (Scheme 43) [122,123].

**Scheme 44:**
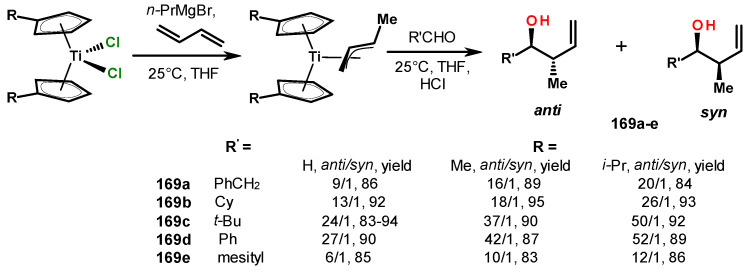
Synthesis of homoallylic alcohols by the reaction of aldehydes with crotyl titanocenes.

Optically active titanocene complexes *p*-*S*,*p*-*S*-**4b** [44] and *p*-*S*,*p*-*S*-**11** [48] were evaluated as catalysts for enantioselective reactions aimed at producing homoallylic alcohols [124,125]. The utilization of various metal complexes in the alkylation of ketones and aldehydes allows for the modulation of stereoselectivity in this process. For example, the reaction of crotyl titanocenes with different substituents in the cyclopentadienyl ring (R = H, Me, *i*-Pr) with primary, secondary, and tertiary aldehydes was described (Scheme 44) [125]. The products of the reaction, homoallylic alcohols **169**, were formed with good to excellent selectivity, consistently favoring the *anti*-diastereomer. The degree of diastereoselectivity correlates with the size of the substituent in the cyclopentadienyl ring of the Ti complex and in the starting aldehyde. Specifically, for aliphatic and aromatic aldehydes, an increase in diastereoselectivity was observed with greater steric hindrance at the metal center.

**Scheme 45:**
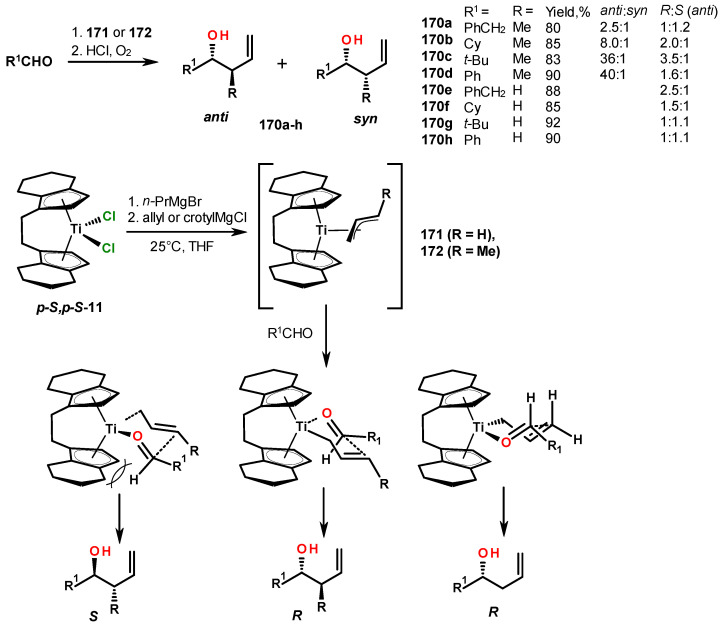
Reaction of allyl- (**171**) and crotyltitanocenes (**172**) with aldehydes.

The reaction of allyl-(**171**) and crotyltitanocenes (**172**), derived from *ansa*-titanocene *p*-*S*,*p*-*S*-11, with aldehydes afforded homoallylic alcohols **170a**–**h** with excellent yields exceeding 80% and a diastereoselectivity of *anti*/*syn* = (2.5–40)/1 (Scheme 45). It was suggested that allyltitanocene additions proceed via six-membered chair- or boat-like transition states, which regulate the reaction stereoselectivity [48,59,125].

**Scheme 46:**
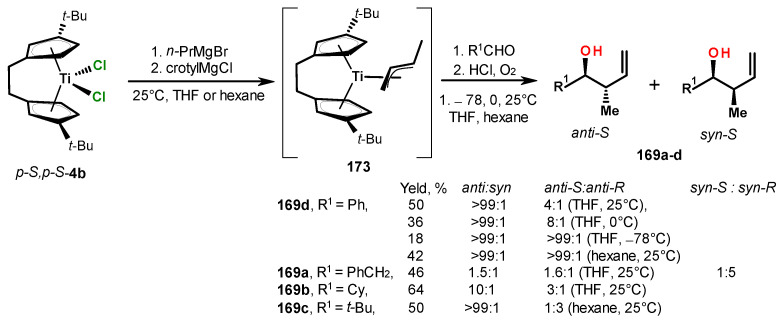
Carbometalation of aldehydes catalyzed by complex *p*-*S*,*p*-*S*-**4b**.

The substitution of the tetrahydroindenyl catalyst *p*-*S*,*p*-*S*-**11** with *ansa*-ethylene-*bis*-(*tert*-butyl-cyclopentadienyl)titanium dichloride *p*-*S*,*p*-*S*-**4b** resulted in a change in absolute configuration from *R*- to *S*- in the reaction products—*anti*-homoallylic alcohols (Scheme 46) [44]. The diastereoselectivity of the process increased with the size of the substituent in the aldehyde (*anti*:*syn* = >99:1). In the reaction with pivaldehyde, the predominance of the *anti-R* enantiomer **169d** was observed. Furthermore, the *syn*-*R*-stereoisomer of homoallylic alcohol **169a** was obtained with greater enantioselectivity than the *anti*-isomer.

**Scheme 47:**
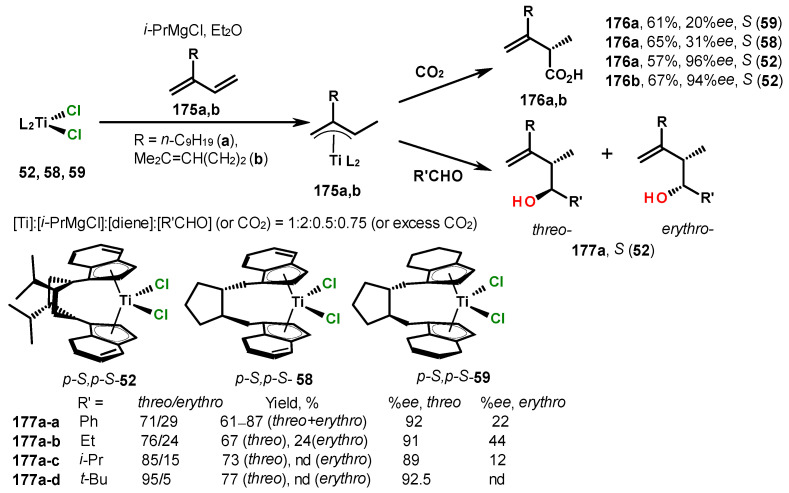
Dia- and enantioselectivity of titanocenes **52**, **58**, and **59** in the reactions of 2-alkyl-1,3-butadienes with CO_2_ or R’CHO.

Titanocenes **52**, **58**, and **59** reacted with 2-alkyl-1,3-butadienes **174a**,**b** and *i*-PrMgCl, yielding allylic complexes **175**, which were then subjected to interaction with excess CO_2_ or R’CHO, resulting in the formation of *S*-enantiomers of carboxylic acids **176a**,**b** or *S*-*threo*/*erythro*-homoallylic alcohols **177**, respectively (Scheme 47) [126]. The highest enantiomeric excess of >91%*ee* was observed in the reaction, catalyzed by complex **52**, with a predominance of *S*-*threo* isomers of the alcohols. In this case, the yield and enantiomeric excess of *erythro-* isomers of the alcohols were low—12–44%*ee*.

**Scheme 48:**
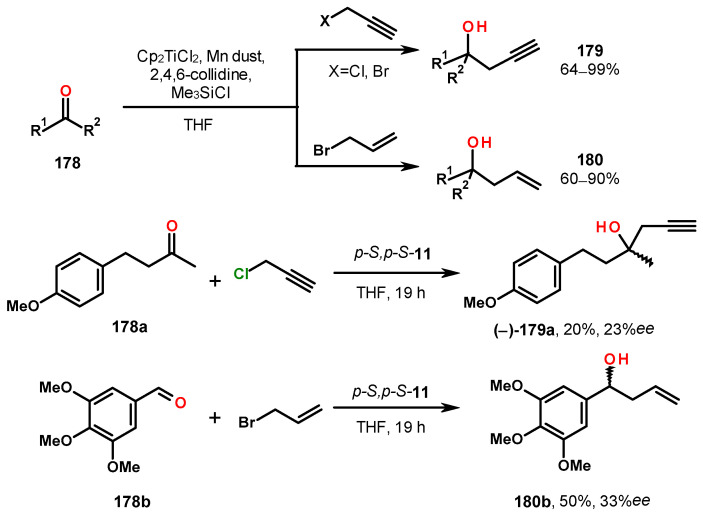
Barbier-type carbonyl compound propargylation or allylation catalyzed with *p*-*S*,*p*-*S*-**11**.

Enantiomerically pure titanocene *p*-*S*,*p*-*S*-**11**, when employed as a reagent in the Barbier-type carbonyl compound propargylation or allylation, yielded homo-propargylic (**179**) or allylic (**180**) alcohols, respectively, demonstrating the fundamental possibility of conducting this reaction in an asymmetric manner (Scheme 48) [127,128,129].

### 3.2. Carbo- and Cycloalumination of Unsaturated Compounds

The asymmetric Zr-catalyzed enantioselective carboalumination of alkenes was first demonstrated by Negishi in 1995 [130,131,132], exemplified by the asymmetric carboalumination of alkenes using AlR_3_ (R=Me, Et) in the presence of the conformationally labile complex *p*-*S*,*p*-*S*-*bis*-neomenthylindenylzirconium dichloride (*p*-*S*,*p*-*S*-**186**), achieving enantioselectivities of up to 85%*ee* for AlMe_3_ and 92%*ee* for AlEt_3_, respectively.

**Scheme 49:**
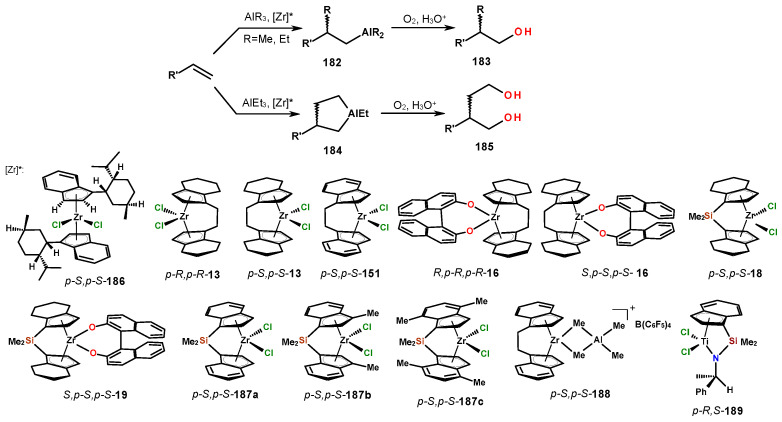
Asymmetric Zr and Ti-catalyzed enantioselective carboalumination and cycloalumination of alkenes.

Studies on the enantioselectivity of *ansa*-complexes *p*-*S*,*p*-*S*-**13**, *p*-*S*,*p*-*S*-**16**, *p*-*S*,*p*-*S*-**18**, *p*-*S*,*p*-*S*-**19** [51], *p*-*R*,*p*-*R*-**13**, *p*-*R*,*p*-*R*-**16** [130], *p*-*S*,*p*-*S*-**151**, *p*-*S*,*p*-*S*-**187a**–**c**, *p*-*S*,*p*-*S*-**188** [132], and *p*-*R*,*p*-*S*-**189** [133] indicated the dependence of the stereodifferentiating effect on the structure of the substrate and the catalyst (Table 2, Scheme 49).

It has been demonstrated [132] that the carboalumination of styrene and allylbenzene by AlMe_3_ in the presence of 10–13 mol% of *p*-*S*,*p*-*S*-**151** or *p*-*S*,*p*-*S*-**187a**–**c** proceeded with conversions of the starting alkenes in the range of 72–89% over 3–6 h. The highest enantioselectivity of 80%*ee* was achieved in the reaction with styrene, then the content of the *p*-*S*,*p*-*S*-**188** complex, activated by [Ph_3_C][B(C_6_F_5_)_4_], was increased to 26 mol%. The methylalumination of allylbenzene, followed by oxidation of the reaction mixture, afforded 2-methyl-3-phenyl-1-propanol with an enantiomeric excess of 25–33%*ee*. The transition from an ethylene bridge to a dimethylsilylene bridge in the *p*-*S*,*p*-*S*-**151** and *p*-*S*,*p*-*S*-**187a** complexes, along with the introduction of methyl groups at the 4,7- or 3- positions of the indene ligands in the *p*-*S*,*p*-*S*-**187b** and *p*-*S*,*p*-*S*-**187c** complexes, did not lead to significant changes in the enantioselectivity of the reaction.

**Scheme 50:**
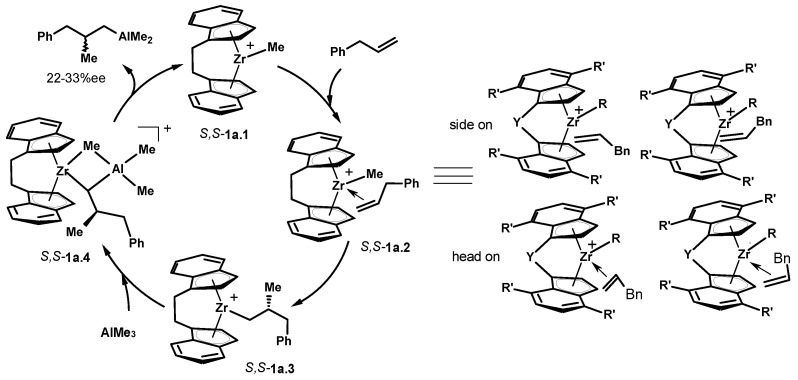
Mechanism of alkene methylalumination in the presence of enantiomerically pure *ansa*-zirconocene complexes.

To elucidate this phenomenon, the mechanism of alkene methylalumination in the presence of enantiomerically pure *ansa*-zirconocene complexes was proposed (Scheme 50) [132]. According to Scheme 50, the starting *ansa*-complex in the presence of MAO provides an activated state—the cationic complex *S*,*S*-**1a.1**. Subsequent coordination and insertion of the olefin yield a new alkylzirconium cation *S*,*S*-**1a.3**, whose association with an AlMe_3_ molecule leads to the heterobimetallic complex *S*,*S*-**1a.4**. The target OAC is formed as a result of alkyl exchange in complex *S*,*S*-**1a.4** and its dissociation. It is suggested that the higher enantioselectivity of the reaction is due to the coordination stage of the olefin to the activated complex in a “side-on” manner, while lower selectivity is associated with a “head-on” approach. It is noted that, apparently, in complexes *p*-*S*,*p*-*S*-**187b** and *p*-*S*,*p*-*S*-**187c**, the methyl substituents do not possess sufficient volume to obstruct the “head-on” direction. The increase in enantioselectivity of the methylalumination when utilizing styrene is attributed to the enhanced interaction between the ligand framework of the complex and the phenyl fragment of styrene.

**Scheme 51:**
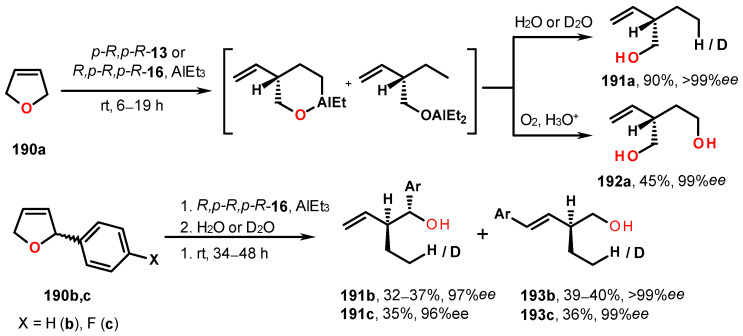
Reaction of 2,5-dihydrofurans with AlEt_3_ catalyzed by complexes *p*-*R*,*p*-*R*-**13** or *R*,*p*-*R*,*p*-*R*-**16**.

The reaction of allylbenzene or styrene with AlEt_3_ catalyzed with (+)-[*η*^5^:*η*^1^-indene dimethylsilyl(α-methylbenzyl)amido]titanium dichloride (*p*-*R*,*S*-**189**) yielded the cycloalumination product **184** with moderate yields of 40–60% and an enantiomeric excess ranging from 19 to 28%*ee* [133]. Remarkably high enantioselectivities of 96–99%*ee* were observed in the reaction of 2,5-dihydrofurans with AlEt_3_, catalyzed by the complexes *p*-*R*,*p*-*R*-**13** or *R*,*p*-*R*,*p*-*R*-**16** (Scheme 51) [137].

The methylalumination of terminal alkenes with AlMe_3_ catalyzed by the binaphtholate complex bearing Si-bridged ligands *p*-*S*,*p*-*S*-**18**, activated by MAO, afforded the corresponding products with a yield of 66% and an enantiomeric excess of 65%*ee* (*R*) [51]. The dichloride complexes *p*-*S*,*p*-*S*-**13** and *p*-*S*,*p*-*S*-**18** exhibited the highest enantioselectivities in carboalumination, achieving 50–51%*ee* (*R*). The enantiomeric purity of cyclic organoaluminum compounds OACs (**185**) formed in reactions catalyzed by complexes *S*,*p*-*S*-**13**, *p*-*S*,*p*-*S*-**18**, *S*,*p*-*S*,*p*-*S*-**16**, and *S*,*p*-*S*,*p*-*S*-**19** ranged between 12 and 26%*ee* (*S*). It is noteworthy that the fixation of the geometry of the zirconocene complex provided methyl- and ethylalumination products with identical configurations at the *β*-stereogenic center but with diminished stereoselectivity compared to the conformationally flexible complex *p*-*S*,*p*-*S*-*bis*-neomenthylindenylzirconium dichloride (*p*-*S*,*p*-*S*-**186**) [135,136].

### 3.3. Asymmetric Oligo- and Polymerization of Alkenes and Dienes

**Scheme 52:**
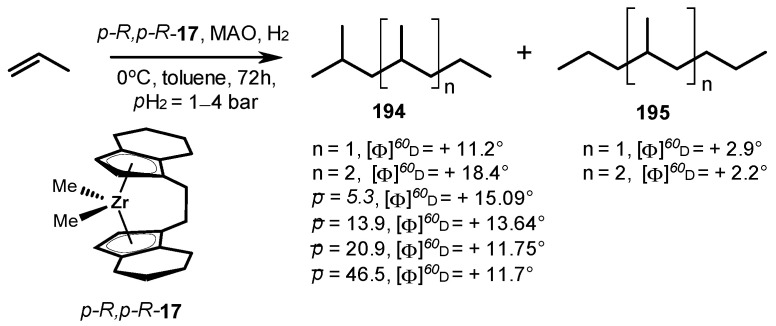
Propene polymerization in the presence of a catalytic system based on *p*-*R*,*p*-*R*-**17** and MAO.

The potential of chiral metallocenes of group IVB metals as catalysts for the stereoselective synthesis of oligomers and polymers from terminal alkenes has been convincingly demonstrated [99,100]. For example, the polymerization of propylene utilizing a catalytic system based on *p*-*R*,*p*-*R*-**17** and methylaluminoxane (MAO) afforded a mixture comprising hydrogenated isotactic polypropylenes **194** and liquid hydrogenated oligomers **195** (Scheme 52) [138,139].

Infrared and ^13^C NMR spectroscopic analyses of the oligomer fractions revealed the presence of terminal *n*-butyl, *n*-propyl, and isobutyl groups, with a predominance of *n*-propyl moieties. Notably, the polymers were devoid of double bonds, thus characterizing the process as “hydrooligomerization.” The hydrooligomers exhibited isotactic configurations, with the first asymmetric carbon atom in the growing oligomer chain possessing an *S*-configuration. Comparison of mass-spectrometry data, as well as ^1^H and ^13^C NMR spectra of the obtained low-molecular-weight hydrooligomers (isolated by distillation of 4,6-dimethyldecane and 2,4,6,8-tetramethylnonane), with the spectra of *rac*-4,6-dimethyldecane and *meso*- and *rac*-2,4,6,8-tetramethylnonane, led to the conclusion that absolute configurations of the stereogenic centers at positions 4 and 6 differ. As a result, *4S*,*6R*-4,6-dimethyldecane and *4R*,*6S*-2,4,6-trimethylnonane were identified.

**Scheme 53:**
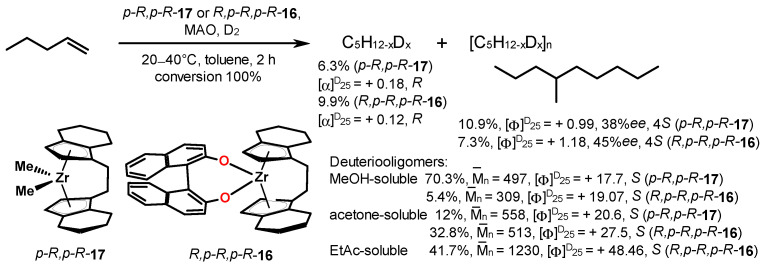
Polymerization of 1-pentene catalyzed by *R*,*p*-*R*,*p*-*R*-**16** or *p*-*R*,*p*-*R*-**17** and MAO, followed by treatment with D_2_.

The polymerization of 1-pentene in the presence of *R*,*p*-*R*,*p*-*R*-**16** or *p*-*R*,*p*-*R*-**17** and MAO, followed by treatment with D_2_, yielded optically active deuterated pentanes of general formula C_5_H_12−x_D_x_ and deuterated oligomers (Scheme 53) [140]. Fractional distillation and successive solvent extractions (methanol, acetone, ethyl acetate) facilitated the isolation of these oligomers. It was proposed that the elongation of the oligomer chain enhances the stereoselectivity of the process, as evidenced by the superior optical purity of the dimers relative to the deuteropentanes. This observation aligns with the hypothesis that stereoselectivity in α-olefin polymerization is governed both by the chirality of the catalytic center and the spatial orientation of the last monomeric unit of the growing chain towards the metal atom [138].

**Scheme 54:**
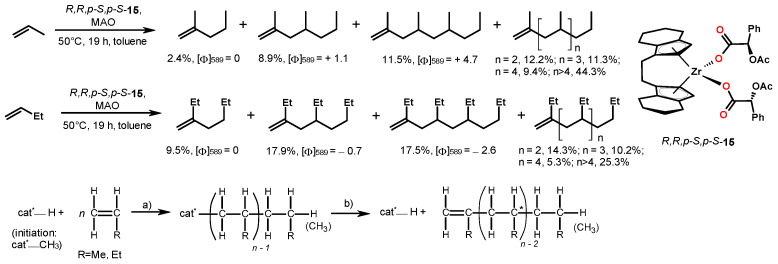
Oligomerization of propene and 1-butene catalyzed by *R*,*R*,*p*-*S*,*p*-*S*-**15** and MAO.

The enantiomerically pure acetylmandelate complex *R*,*R*,*p*-*S*,*p*-*S*-**15**, combined with MAO, was evaluated for propene and 1-butene oligomerization (Scheme 54) [141]. Under increased catalyst concentration and elevated monomer content, isotactic oligomers bearing terminal double bonds were obtained, indicative of an accelerated chain termination rate relative to polymer chain propagation.

Initially, methylaluminoxane mediates the substitution of the mandelate ligand with a methyl group in the complex, generating a Zr–Me bond that facilitates substrate incorporation (Scheme 54). Subsequent olefin coordination promotes chain elongation. *β*-Hydride transfer to the metal center terminates the chain, yielding a complex bearing a free Zr–H bond capable of coordinating another alkene to regenerate the metal alkyl species. Gas chromatographic analysis revealed the presence of oligomers with degrees of polymerization (n) ranging from 2 to 7. Under analogous conditions, propylene predominantly formed pentamers, whereas 1-butene yielded trimers and tetramers. The higher oligomers exhibited isomeric mixtures primarily composed of configurational isomers. For example, the tetramer fraction contained a diastereomeric mixture of (*R*,*R*)/(*S*,*S*)- and (*R*,*S*)/(*S*,*R*)-2,4,6-trimethyldecene in a 5:4 ratio, while the pentamer fraction comprised four pairs of enantiomers of 2,4,6,8-tetraethyl-1-dodecene. Gas chromatographic separation of the enantiomers of the 1-butene trimer mixture was achieved via modified cyclodextrin chromatography [142,143], with an enantiomeric purity of 27%*ee*. Consistent with the hydrooligomerization of propylene, the reaction favored the formation of *R*-enantiomers. The stereoselectivity of the second alkene insertion step was notably enhanced, as the stereogenic centers in the growing oligomer chain enhanced the stereodifferentiating effect (growing chain control).

The catalytic system *p*-*S*,*p*-*S*-**13**-MAO exhibited a high degree of isotacticity in the polymerization of propylene, 1-butene, 1-pentene, and 1-hexene [144]. The elongation of the alkyl substituent exerted minimal influence on isotacticity, which remained between 95 and 99%*mmmm* at temperatures below 20 °C. The optical rotation of higher oligomers diminished with increasing reaction temperature, whereas the enantiomeric excess of the oligomers increased from 10% to 90% as the temperature decreased from 60 °C to 0 °C.

Comparative studies of propene polymerization stereoselectivity in the presence of *R*,*R*,*p*-*S*,*p*-*S*-**15** and a mixture of diastereomers *R*/*S*-**15** revealed that the exchange of polymer chains between metal centers of opposing chirality significantly undermines stereochemical control [145]. In most experiments, the precursor was activated with an optimal quantity of AlBu^i^_3_ and stoichiometric amounts of [Ph_3_C][B(C_6_F_5_)_4_]. Both racemic and enantiopure catalysts displayed comparable activities; however, the racemic catalyst exhibited diminished stereo- and regioselectivity. The stereoselectivity of *R*/*S*-**15** improved markedly upon lowering its concentration, reducing the reaction temperature, or immobilization on silica supports. It was concluded that the racemate forms two catalytically active centers with different configurations capable of exchanging polymer alkyl chains, thereby reducing stereoselectivity and increasing regioerrors.

Furthermore, the catalytic activity and stereoselectivity of chiral homogeneous and heterogeneous catalytic systems were compared [146]. The optically active heterogeneous catalyst, prepared by depositing TiCl_3_ on MgCl_2_ in the presence of di[(*S*)-2-methylbutyl]phthalate as an electron donor, exhibited moderate activity in racemic 4-methyl-1-hexene (4MH) polymerization (2.6·10^3^ g polymer/(mol Ti·mol 4MH·h)). The (*4S*)-4-methylhexene enantiomer was incorporated more favorably than the (*R*)-enantiomer, with a polymerization rate ratio *R*(*S*)/*R*(*R*) of 1.02 and a polymer rotation angle [α]_D_^25^ of +4.8°. Consequently, the residual monomer was enriched in the *R*-enantiomer. In contrast, the homogeneous catalytic system *R*,*R*,*p*-*S*,*p*-*S*-**15**-MAO exhibited a higher rate ratio of 1.4 and a polymer rotation angle of +17 to +19°, with an activity of 112·10^3^ g polymer/(mol Zr·mol 4MH·h). The stereoregularity index (SI) was lower in heterogeneous catalysis (maximum 85%) than in the homogeneous system (maximum 97%). Fractionation of polymer in various solvents (acetone, ethyl acetate, diethyl ether, and cyclohexane) yielded fractions with distinct rotation angles. For instance, fractions obtained in the system *R*,*R*,*p*-*S*,*p*-*S*-**15**-MAO displayed [α]_D_^25^ values of –7.8° (acetone), +22° (ethyl acetate), and +31° (cyclohexane), whereas the TiCl_3_-MgCl_2_-Et_3_Al-di[(*S*)-2-methylbutyl]phthalate system yielded positive rotations of +5.4°, +10°, +15°, and +9.9° in the respective solvents. This disparity, including the inversion of rotation sign, was attributed to stereoerrors, i.e., the incorporation of both *S*- and *R*-enantiomers under specific conformations of the catalytically active centers in homogeneous systems—a phenomenon more prevalent at elevated temperatures due to increased conformational flexibility. Conversely, heterogeneous catalysis features fixed geometries of active centers, albeit with greater diversity in Lewis acidity and steric environments.

**Scheme 55:**
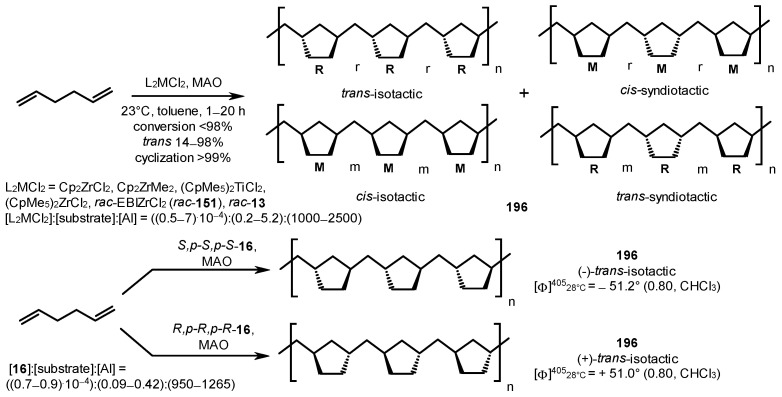
Cyclopolymerization of 1,5-hexadiene catalyzed by achiral precatalysts Cp_2_MX_2_ (M = Ti, Zr; X = Cl, Me) and MAO.

Cyclopolymerization of 1,5-hexadiene catalyzed by achiral precatalysts Cp_2_MX_2_ (M = Ti, Zr; X = Cl, Me) and MAO at ambient temperature afforded poly(methylene-1,3-cyclopentane) (PMCP) **196**, which can exhibit four possible microstructural variants (Scheme 55) [147]. Among these, only the *trans*-isotactic microstructure is chiral. According to ^13^C NMR data, approximately 80% of PMCP rings adopted the *trans*-configuration. Lowering the polymerization temperature enhanced *trans*-selectivity, reaching 90% *trans* rings at –78 °C. In contrast, sterically hindered complexes (CpMe_5_)_2_MX_2_ (Cp* = pentamethylcyclopentadienyl) predominantly yielded *cis*-PMCP, with 86% *cis* content at –25 °C. Cyclooligomerization of 1,5-hexadiene catalyzed by chiral catalysts *rac*-**13** and *rac*-**151** afforded PMCP with 70% trans content (Scheme 55). Here, the *cis*/*trans* stereochemistry was less sensitive to polymerization temperature compared to achiral metallocenes. Enantiomerically pure precatalysts *R*,*p*-*R*,*p*-*R*-**16** and *S*,*p*-*S*,*p*-*S*-**16** produced optically active *trans*-isotactic PMCP **196** with molar optical rotations [Φ]_405_^28^ = +51.0° and [Φ]_405_^28^ = −51.2°, respectively.

**Scheme 56:**
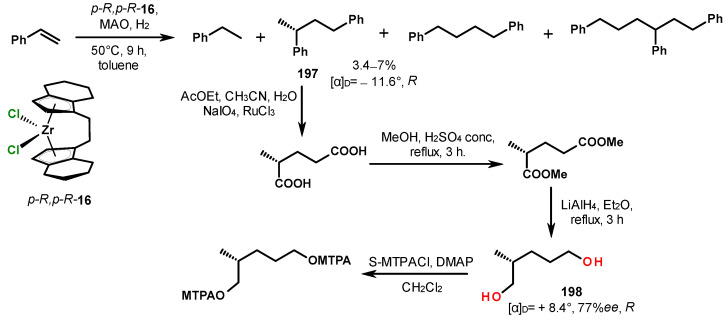
Synthesis of enantiomerically enriched styrene hydrooligomers catalyzed by complex *p*-*R*,*p*-*R*-**16**.

Enantiomerically enriched styrene hydrooligomers were synthesized via catalysis by complex *p*-*R*,*p*-*R*-**16**, activated with methylaluminoxane under a hydrogen atmosphere [148]. The oligomers included *R*-1,3-diphenylbutane (**197**) with an enantiomeric excess of 77%*ee* (Scheme 56). Subsequent functionalization without loss of chirality underscores the potential of this catalytic system for preparing optically active synthons for organic synthesis.

**Scheme 57:**
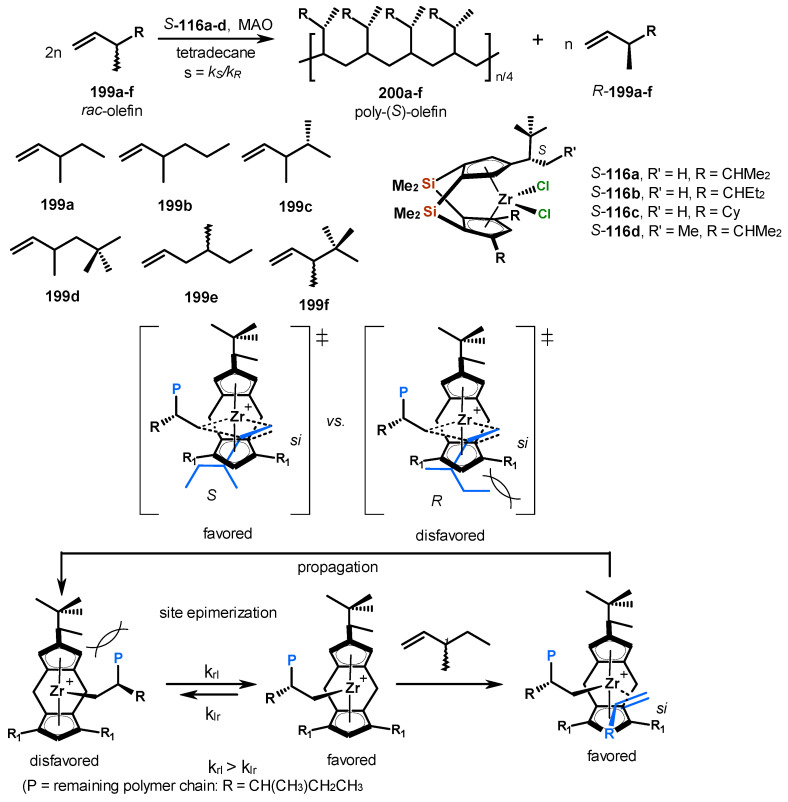
Kinetic resolution of racemic α-olefins bearing bulky substituents during polymerization catalyzed by enantiomerically pure *S*-**116a**–**d**.

Polymerization of racemic α-olefins bearing bulky substituents **199a**–**f** catalyzed by enantiomerically pure *S*-**116a**–**d** in the presence of MAO achieved kinetic resolution, yielding the *R*-enantiomers of **199a–f** with enantiomeric excesses of 7–59%*ee* and chiral, highly isotactic polymers **200a**–**f** enriched in the *S*-enantiomer (Scheme 57, Table 3) [81,86,149,150,151]. The selectivity factor s (rate of the fast-reacting enantiomer)/(rate of the slow-reacting enantiomer) for most olefins ranged from approximately 1.1 to 3.2, indicating partial kinetic resolution; however, polymerization of 3,4-dimethyl-1-pentene (**199c**) exhibited pronounced resolution (s > 15) [150,151]. To rationalize isotactic polymer formation, the authors proposed epimerization of the catalytically active center, whereby the polymer chain alternates sides relative to the metallocene cation after each monomer insertion. A high rate of site epimerization relative to olefin coordination ensures that the olefin encounters a consistent chiral pocket, accounting for the high stereoselectivity. The stereogenic centers in the growing polymer chain may further influence the stereoselectivity of the process.

**Scheme 58:**
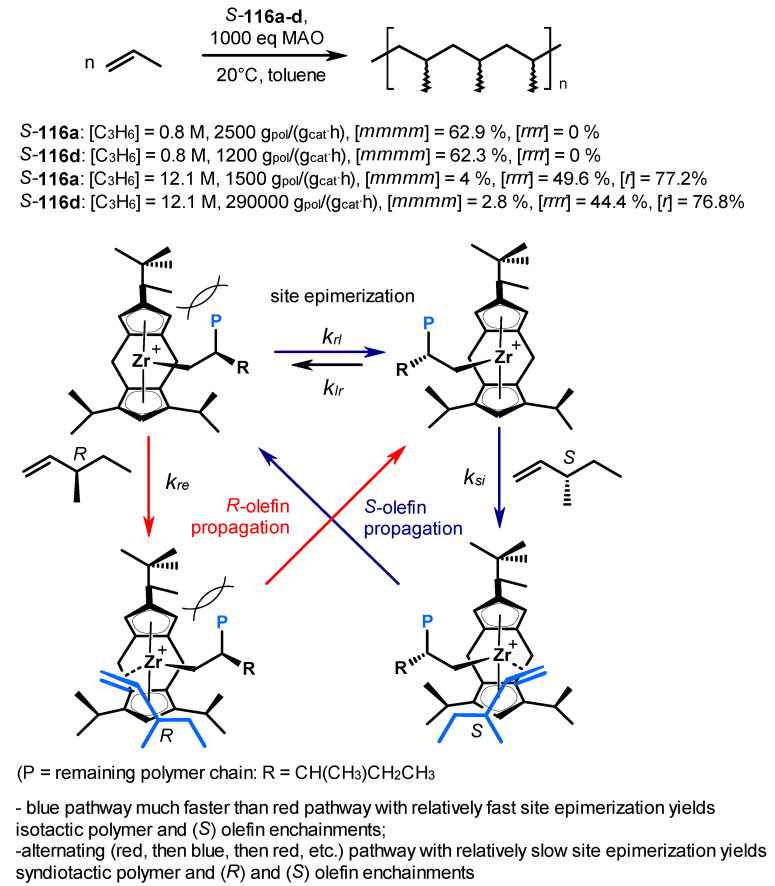
Stereoselectivity control model of propene polymerization catalyzed by enantiomerically pure *S*-**116a**–**d**.

In the presence of catalysts **116a** and **116d**, the stereoselectivity of propene polymerization was markedly influenced by substrate concentration ([*mmmm*] = 2.8–62.9%, [*rrrr*] = 6.8–49.6%) (Scheme 58) [150]. Elevated isotacticity was attained at diminished monomer concentrations ([*mmmm*] = 62%), whereas conducting the reaction in neat propylene yielded a syndiotactic polymer ([*rrrr*] = 44–49%). At intermediate concentrations, catalyst **116d** favored a higher proportion of isotactic polymer relative to **116a**. Moreover, **116d** exhibited enhanced selectivity during the kinetic resolution of 3-substituted racemic α-olefins.

To rationalize these observations, it is noted that the polymerization of racemic α-olefins likely proceeds via the involvement of a pair of diastereomeric pairs at each catalytic site (even more if chain end control is taken into account), instead of involving just a pair of diastereomers as observed in simple α-olefin polymerizations [150]. Consequently, the selectivity in racemic polymerization is governed by matching and mismatching pairs in a double diastereoselective manner. The proposed enantiomorphic site control model (Scheme 58) incorporates a double diastereoselective transition state—for instance, the (*S*)-metallocene/*si*-olefin face/(*S*)-olefin diastereomer is favored over the (*S*)/*si*/(*R*) diastereomer. Nonetheless, the scenario is more complex, especially when considering potential insertions from both faces of the zirconocene, chain end control, and/or poor enantiofacial selectivity. Additionally, the rate of anion reorganization during racemic α-olefin polymerization may also contribute significantly.

**Scheme 59:**
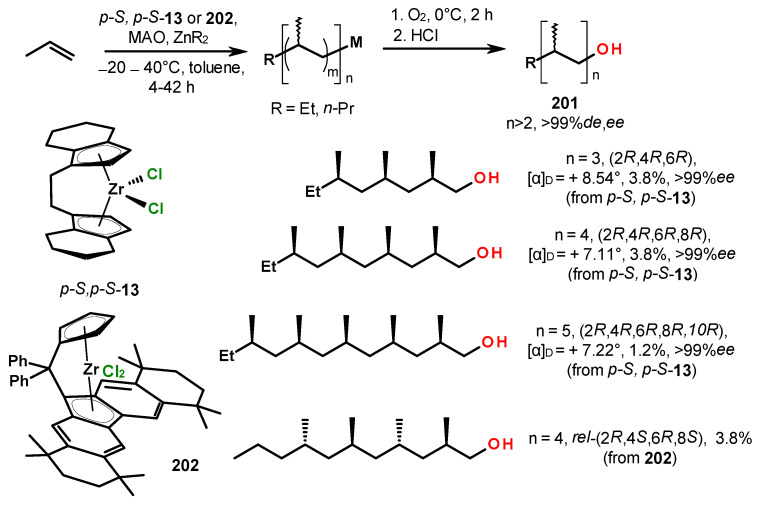
Stereocontrolled oligomerization of propylene via reaction with ZnEt_2_ in the presence of *p*-*S*,*p*-*S*-**13** and MAO.

Subsequently, a one-pot method for the synthesis of the deoxypropionate scaffold was developed based on the stereocontrolled oligomerization of propylene (Scheme 59) [152]. It was demonstrated that the reaction of propylene with ZnEt_2_ in the presence of optically active zirconocene *p*-*S*,*p*-*S*-**13** and MAO afforded oligomers. Under identical conditions, the employment of AlMe_3_ failed to yield the target products. Following oxidation and hydrolysis, branched primary alcohols **201** were obtained with high enantiomeric purity (99%*ee*), albeit with modest yields due to the formation of polymerization side products. The C_S_-symmetric zirconocene **202** catalyzed syndiospecific oligomerization under analogous conditions, delivering *rel*-(*2R*,*4S*,*6R*,*8S*)-2,4,6,8-tetramethylundecanol-1 [153], a constituent of *Antitrogus parvulus* (Scheme 59).

**Scheme 60:**
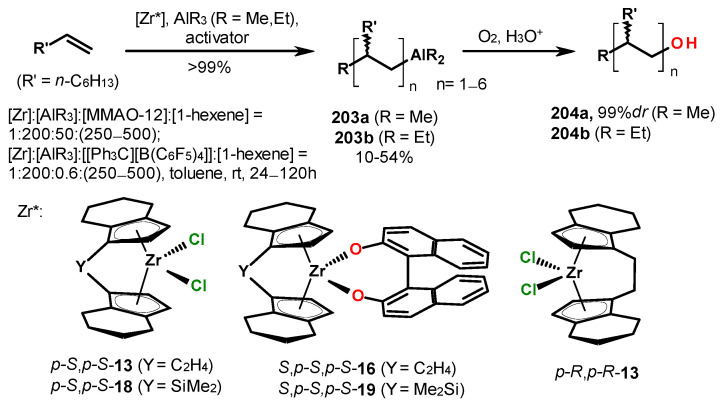
Oligomerization of 1-hexene catalyzed by enantiomerically pure Zr *ansa*-complexes.

Oligomerization of 1-hexene catalyzed by enantiomerically pure Zr *ansa*-complexes *p*-*S*,*p*-*S*-**13,** *p*-*S*,*p*-*S*-**18,** *S*,*p*-*S*,*p*-*S*-**16**, and *S*,*p*-*S*,*p*-*S*-**19** in the presence of AlR_3_ (R = Me, Et) and MAO yielded optically active, functionally substituted oligomers **204a**,**b** with degrees of oligomerization up to six and yields ranging from 10 to 54% (Scheme 60) [51].

In developing this methodology, stereoselective oligomerization of 1-hexene was achieved by employing *p*-*R*,*p*-*R*-**13**, AlMe_3_, and activators such as MMAO-12 or [Ph_3_C][B(C_6_F_5_)_4_]. This approach yielded organometallic intermediates **203a** bearing an initial methyl group, which upon oxidation and hydrolysis furnished alcohols **204a** (Scheme 60) [154]. In the presence of MMAO-12, the oligomer fraction (**204a**, n = 2–6) constituted 63–81%, whereas the use of [Ph_3_C][B(C_6_F_5_)_4_] as an activator enhanced oligomer yields to 94–98%. The functionally substituted oligomers **204a** exhibited enantiomeric enrichment and diastereomeric purity (99%*dr*). An increase in oligomer chain length correlated with augmented enantioselectivity, irrespective of activator type. Nevertheless, the nature of the activator significantly influenced enantioselectivity. Specifically, reactions with AlMe_3_ and MMAO-12 proceeded with moderate enantioselectivity—60–64%*ee* for oligomers containing two monomeric units, and 78–84%*ee* for oligomers with n = 3,4—reaching 93%*ee* for n = 5, 6. Substitution of MMAO-12 with [Ph_3_C][B(C_6_F_5_)_4_] diminished enantioselectivity to an average of 10–20%*ee*. Therefore, stereoselectivity in these catalytic systems is governed by both the metal center and the growing polymer chain, which is consistent with the Corradini model [155]. The experimental data further underscore the pivotal role of counterion effects during alkene insertion into catalytically active centers of the type [L_2_Zr^+^Alk…SLA] (SLA—strong Lewis acid).

Despite substantial progress in stereoselective olefin polymerization, enantioselective oligomerization and polymerization of alkenes remain comparatively underexplored. Significant advancements have been realized employing enantiomerically pure *ansa*-zirconocenes. Mechanistic descriptions frequently invoke multiple conformations of catalytically active centers during both alkene coordination and chain propagation, implying that reaction stereoselectivity is largely dictated by the ligand environment surrounding the transition metal and the growing polymer chain. Recently, an increasing imperative has emerged to scrutinize the influence of activator structure on the organization of catalytically active centers, and consequently, on the stereochemical outcome of these transformations.

### 3.4. The Diels–Alder Cycloaddition

**Scheme 61:**
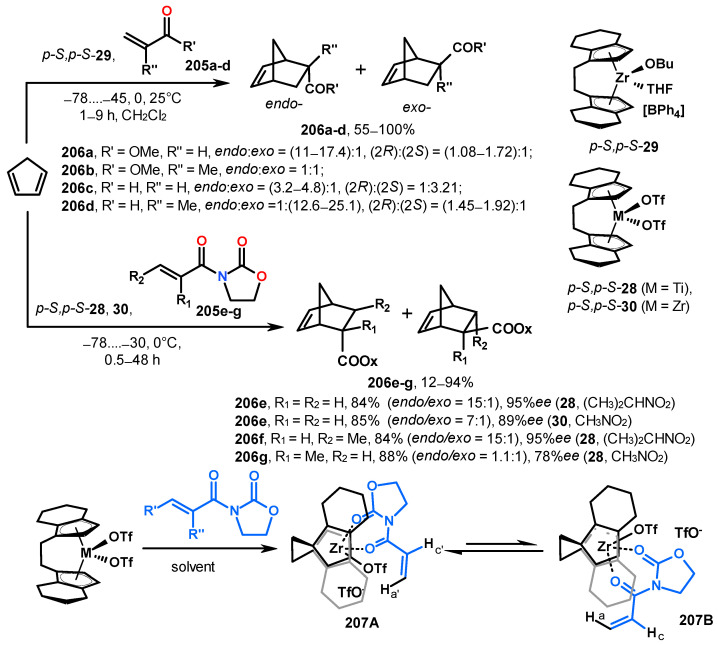
Cycloaddition of enones with cyclopentadiene catalyzed by chiral Ti and Zr complexes.

Enantiomerically pure zirconium *bis*-alkoxy or triflate *ansa*-complexes serve as catalysts for Diels–Alder cycloadditions between cyclopentadiene and various dienophiles, exemplified by **205a**–**g** (Scheme 61) [58]. Reactions involving dienophiles bearing ethenyl substituents (**205a**,**c**) preferentially yield the *endo*-product. The *endo*/*exo* product ratio depends on the substituent’s nature at the double bond. In all cases, enantioselectivity did not exceed 50%*ee*. The authors attribute the low enantioselectivity of the reactions to both the steric factors of the substrate (*endo*- and *exo*selectivity) and the steric environment of the transition metal center.

The cycloaddition of enones **205e**–**g** with cyclopentadiene catalyzed by chiral triflate complexes Ti (*p*-*S*,*p*-*S*-**28**) and Zr (*p*-*S*,*p*-*S*-**30**) predominantly afforded *R*-*endo* isomers **206e**–**g**, with enantiomeric excess heavily influenced by solvent polarity (Scheme 61) [60]. In dichloromethane, enantioselectivity was below 69%*ee* (*R*), whereas in nitromethane or 2-nitropropane, stereoselectivity reached 90–95%*ee* (*R*, *endo*/*exo* = (6–15)/1) [59]. The degree of asymmetric induction in this reaction was ascribed to the conformations of the intermediates—coordination products of the *ansa*-complex and the starting oxazolidinone substrate **205e**–**g** (Scheme 61) [60]. Spectroscopic (EXSY, NOESY) and kinetic analyses revealed the formation of two isomeric complexes upon substrate–catalyst binding. The relative reactivities of these intermediates determine the diastereo- and enantioselectivity of the reaction. Thus, solvent-dependent stereoselectivity likely arises from the differential stability of complexes **207A** and **207B**. It is plausible that the minor isomer **207B** reacts with cyclopentadiene more rapidly and selectively, favoring the formation of *endo* enantiomers **206e**–**g**.

### 3.5. Reactions of Imine ansa-Zirconocenes—Zirconaaziridines

**Scheme 62:**
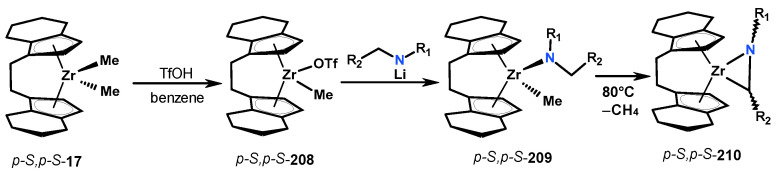
Synthesis of zirconaaziridines from dimethyl complex *p*-*S*,*p*-*S*-**17**.

Metal–carbon bonds exhibit pronounced reactivity toward organic substrates and serve as pivotal intermediates in many catalytic processes. Since 1991, asymmetric catalysis employing imine complexes of *ansa*-zirconocenes—termed zirconaaziridines—has been reported. The synthesis of zirconaaziridines is typically conducted in situ (Scheme 62). For example, the reaction of the dimethyl complex *p*-*S*,*p*-*S*-**17** with trifluoromethanesulfonic acid and lithium amide provided zirconocene methyl amide **209**. Subsequent methane elimination at 80 °C yielded zirconaaziridine **210** [50,156].

**Scheme 63:**
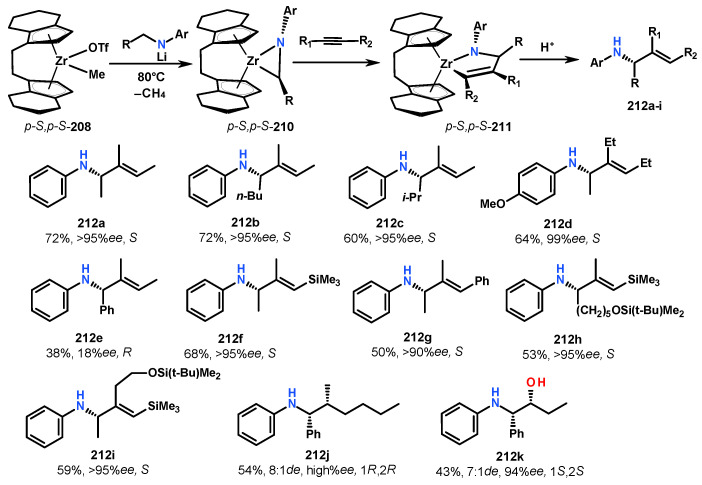
Asymmetric induction in the reaction of amines with alkynes, alkenes, or aldehydes catalyzed by zirconaaziridines **210**.

A diverse array of heteroatom-containing compounds has been synthesized via reactions of zirconaaziridines with unsaturated substrates. Notably, asymmetric induction in the reaction of amines with alkynes, alkenes, or aldehydes in the presence of zirconaaziridines **210** has been demonstrated, yielding allylic amines **212a**–**i** and phenylalkyl amines **212j**,**k** with respectable yields (3872%) and high enantioselectivity (Scheme 63) [50].

**Scheme 64:**
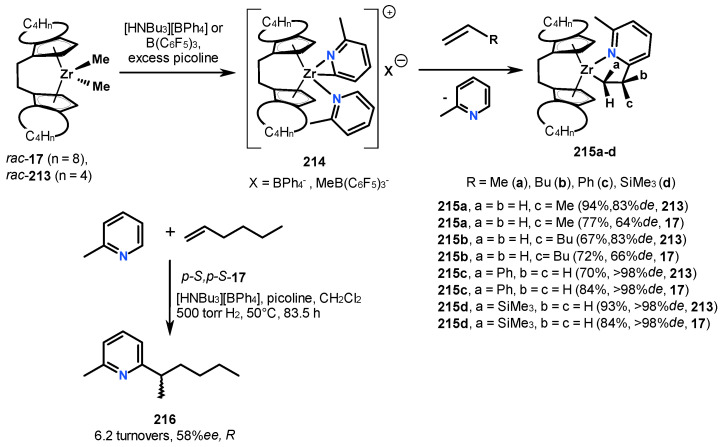
Reactions of complexes **214** with various alkenes yielding metallocycles **215a**–**d** and synthesis of substituted pyridine **216** catalyzed by *p*-*S*,*p*-*S*-**17**.

Reactions of complexes **214** with various alkenes furnished metallocycles **215a**–**d** in yields ranging from 67% to 94% (Scheme 64) [157]. Diastereoselectivity in reactions with aliphatic alkenes was enhanced using the *bis*-indenyl catalyst *rac*-**213**, achieving up to 83%*de*, whereas the tetrahydroindenyl analog *rac*-**17** exhibited diminished stereoselectivity (66%*de*). Employing styrene or vinyltrimethylsilane as substrates altered regioselectivity and augmented diastereoselectivity to 98%*de*, independent of catalyst structure. Reaction of 1-alkenes with excess picoline in the presence of optically active dimethylzirconocene *p*-*S*,*p*-*S*-**17** and [HNBu_3_][BPh_4_] afforded substituted pyridine **216** with 58%*ee* (*R*) (Scheme 64).

**Scheme 65:**
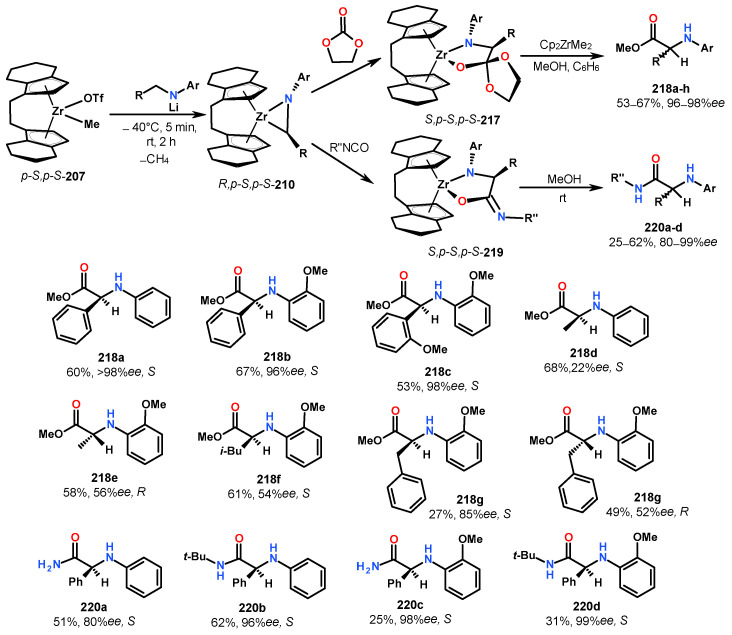
Reactions of alkyl- or aryl-substituted zirconaziridine with ethylene carbonate or isocyanates yielding amino acid esters.

Reactions of alkyl- or aryl-substituted zirconaziridine **210** with ethylene carbonate or isocyanates produced amino acid esters **218a**–**h** with enantiomeric purities up to 98%*ee*, and phenylglycinamides **220a**–**d** with 80-99%*ee*, respectively (Scheme 65) [156]. In these transformations, the *ansa*-complex functions both as catalyst and substrate, necessitating stoichiometric amounts of the initial complex.

**Scheme 66:**
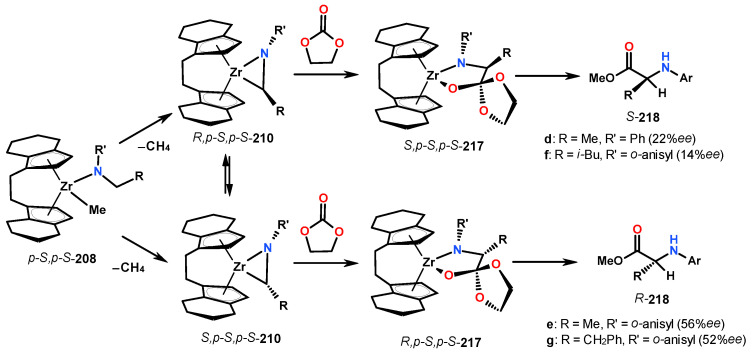
Proposed mechanism of stereoinduction in the reaction of substituted zirconaziridine with ethylene carbonate.

In discussing the plausible mechanism of stereoinduction, a hypothesis was proposed correlating enantioselectivity with the equilibrium between epimers of zirconaziridine **210**. This ratio can be modulated by varying reagent quantities (ethylene carbonate or isocyanate), reaction parameters (temperature, solvent), or substrate substituents (Scheme 66).

**Scheme 67:**
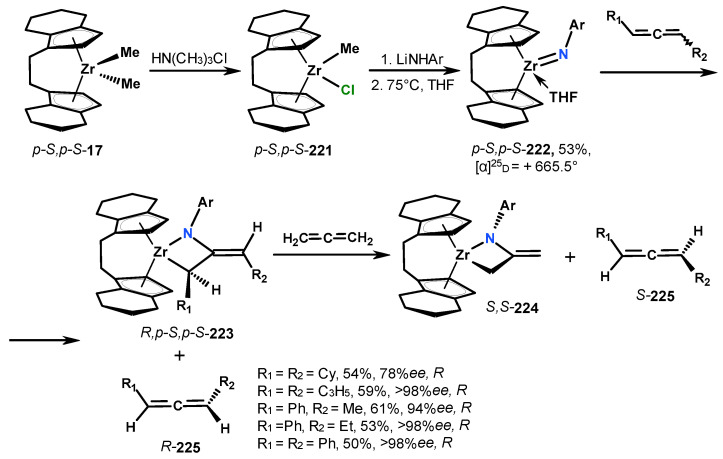
Reaction of the enantiomerically enriched imido-complex *p*-*S*,*p*-*S*-**222** with allenes.

Furthermore, it was demonstrated that the reaction of enantiomerically enriched imido-complex *p*-*S*,*p*-*S*-**222** with allenes proceeds via kinetic resolution, yielding a single *R*-enantiomer of allene **225** with 50–61% yield and 7898%*ee* (Scheme 67) [158]. The more reactive allene enantiomer was subsequently regenerated from the metallacycle by treatment with the parent allene, 1,2-propadiene. Thus, the kinetically favored *S*-allene enantiomer was recovered from metallacycle *R*,*p*-*S*,*p*-*S*-**223** via reaction with 1,2-propadiene.

### 3.6. Cyclization Reactions

**Scheme 68:**
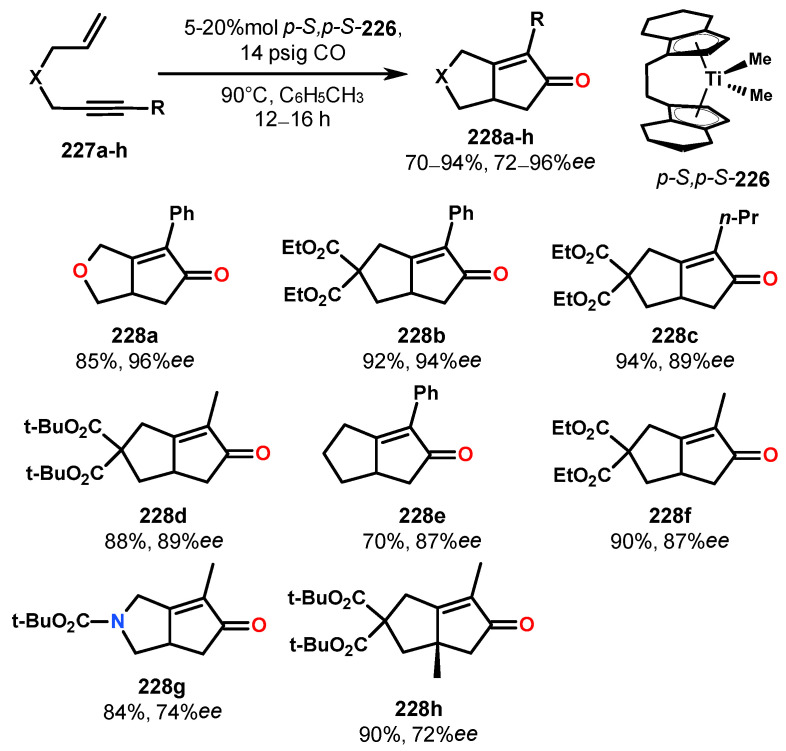
Pauson–Khand cyclo-condensation of enynes with carbon monoxide catalyzed with *p*-*S*,*p*-*S*-**226**.

Complexes of Group IVB metals, similar to cobalt complexes, facilitate the Pauson–Khand cyclo-condensation of enynes with carbon monoxide, yielding cyclopentenones. The first examples of this reaction being carried out in an asymmetric manner are presented in Refs. [159,160] (Scheme 68). It was found that *p*-*S*,*p*-*S*-**226** serves as a selective catalyst for transforming various 1,6-enynes **227a**–**h** in the presence of CO into the corresponding bicyclic cyclopentenones **228a**–**h**, achieving yields of 70–94% and enantioselectivity of 72–96%*ee*.

**Scheme 69:**
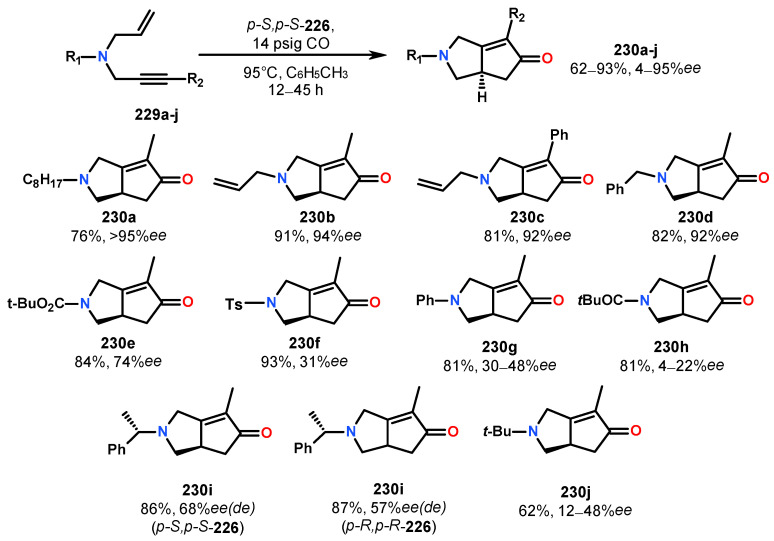
Asymmetric cyclocarbonylation of nitrogen-containing enynes catalyzed with *p*-*S*,*p*-*S*-**226**.

Subsequently, a method for the asymmetric cyclocarbonylation of nitrogen-containing enynes was developed (Scheme 69) [161]. Studies of the effects of substituents at the nitrogen atom and catalyst concentration on the enantioselectivity of this type of cyclization revealed that enynes **229a**–**j** undergo cyclization in the presence of CO and catalytic amounts of *p*-*S*,*p*-*S*-**226**, producing azabicyclo[3.3.0]oct-5-en-7-ones **230a**–**j** with yields exceeding 62% and enantioselectivity up to 95%*ee*. It was demonstrated that reactions involving enynes with N-octyl, N-allyl, or N-benzyl groups exhibited high enantioselectivity. In contrast, the presence of an anilino, amide, or sulfonamide moiety in the starting molecules resulted in products with lower ee values. When nitrogen was part of a carbamate group, moderate enantioselectivity was observed. It is suggested that substrates with a relatively electron-rich nitrogen center cyclize to enones with higher enantioselectivity compared to those with an electron-deficient nitrogen atom.

**Scheme 70:**
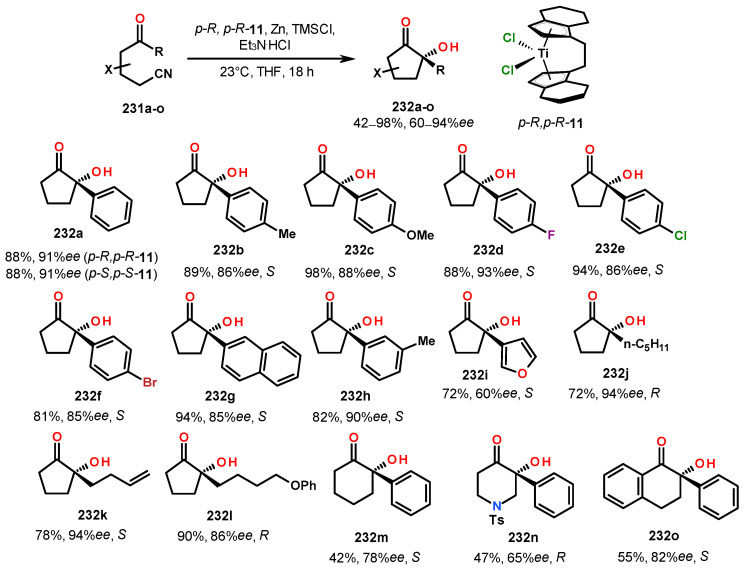
Intramolecular asymmetric reductive coupling of ketones with nitriles catalyzed by *p*-*R*,*p*-*R*-**11**.

The intramolecular asymmetric reductive coupling of ketones with nitriles, catalyzed by the optically active titanocene *p*-*R*,*p*-*R*-**11**, was developed. This method yields substituted α-hydroxyketones (acyloins)—structural fragments of numerous natural compounds. Cyclic hydroxy-substituted cyclopentanones **232a**–**o** were synthesized with commendable yields ranging from 47% to 94%, with enantioselectivity exceeding 65%*ee* (Scheme 70) [162].

### 3.7. Asymmetric Hydrosilylation of Ketones

**Scheme 71:**
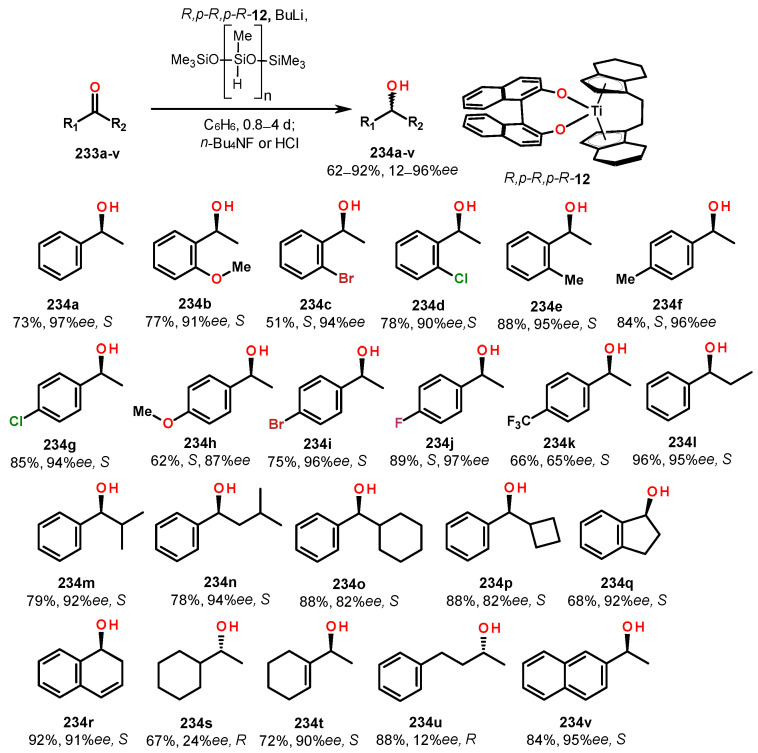
Asymmetric hydrosilylation of ketones by polymeric methylhydrosiloxane Me_3_SiO[MeSi(H)O]_n_SiMe_3_ in the presence of *R*,*p*-*R*,*p*-*R*-**12**.

The asymmetric hydrosilylation [163] of ketones **233a**–**v** using polymeric methylhydrosiloxane Me_3_SiO[MeSi(H)O]_n_SiMe_3_ in the presence of chiral titanocene binaphtholates *R*,*p*-*R*,*p*-*R*-**12** proceeded with high enantioselectivity (82–96%*ee*) (Scheme 71) [164]. Among these, ketones lacking a double bond at the α-position (**234s**,**u**) exhibited poor enantioselectivity. Furthermore, it was demonstrated that employing substrates with bulky alkyl groups in the alkylaryl ketone structure enhanced enantiomeric excess. Generally, conducting the reaction with branched substrates necessitated an additional catalyst amount (up to 10 mol%) and extended reaction times.

**Scheme 72:**
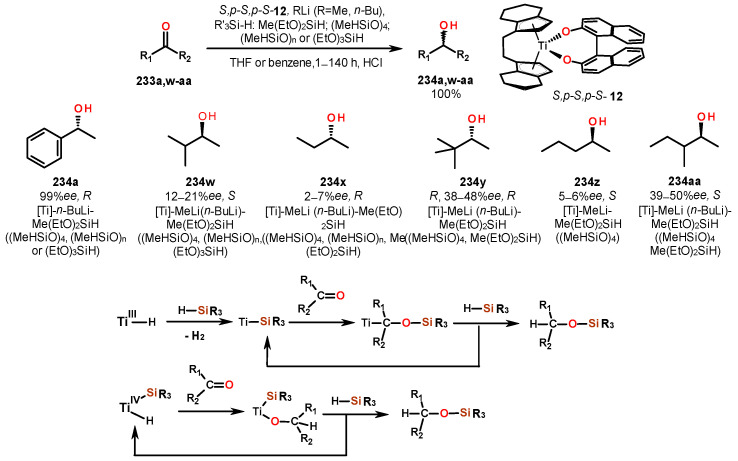
Hydrosilylation of ketones by silanes catalyzed with alkylated *S*,*p*-*S*,*p*-*S*-**12**.

Hydrosilylation of ketones was performed using silanes (EtO)_3_SiH, Me(EtO)_2_SiH, [MeSi(H)O]_4_, Me_3_SiO[MeSi(H)O]_n_SiMe_3_, and MeSiH_3_, alongside catalysts prepared by alkylation of *S*,*p*-*S*,*p*-*S*-**12** with MeLi or *n*-BuLi (Scheme 72) [165]. The enantiomeric purity of the acetophenone hydrosilylation product **234a**, obtained in the presence of the *S*,*p*-*S*,*p*-*S*-**12**-*n*-BuLi system, was significantly higher (approximately 99%*ee*) than that achieved with the MeLi-alkylated catalyst (approximately 40–50%*ee*). The hydride complex [(EBTHI)TiH]_2_, synthesized in the reaction of *S*,*p*-*S*,*p*-*S*-**12** with MeLi and PhMeSiH_2_, exhibited essentially the same enantioselectivity as the MeLi-based catalyst. Since the titanocene hydride demonstrated low enantioselectivity in the acetophenone hydrosilylation, the authors proposed two potential reaction mechanisms involving TiIII and TiIV species (Scheme 72). In these mechanisms, key intermediates were suggested to be species with Ti-Si bonds, whose coordination with the substrates determines both the reaction rate and stereoselectivity. The observed differences in enantiomeric excess with MeLi and *n*-BuLi were attributed to the rapid decomposition of L_2_TiBu_2_ alongside Ti reduction, in contrast to the L_2_TiMe_2_ complexes, as well as the direct involvement of Ti alkyl complexes as intermediates.

**Scheme 73:**
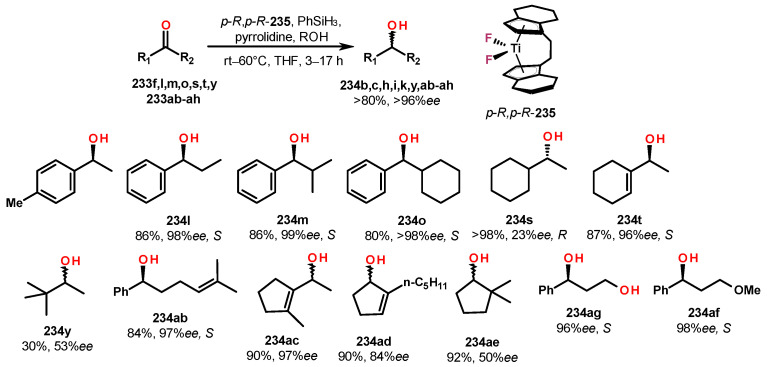
Titanocene *p*-*R*,*p*-*R*-**235** as an effective catalyst for the hydrosilylation of ketones by PhSiH_3_.

Optically active titanocene difluoride *p*-*R*,*p*-*R*-**235** was found to be an effective catalyst for the hydrosilylation of ketones by PhSiH_3_ in the presence of alcohol additives (Scheme 73) [166,167]. It has been shown that primary alcohols were more effective as additives than secondary alcohols or primary amines. For example, the gradual addition of methanol to the reaction mixture resulted in an increased conversion of the starting ketone (99%) and the enantiomeric purity of the synthesized secondary alcohols (99%*ee*) [166].

**Scheme 74:**
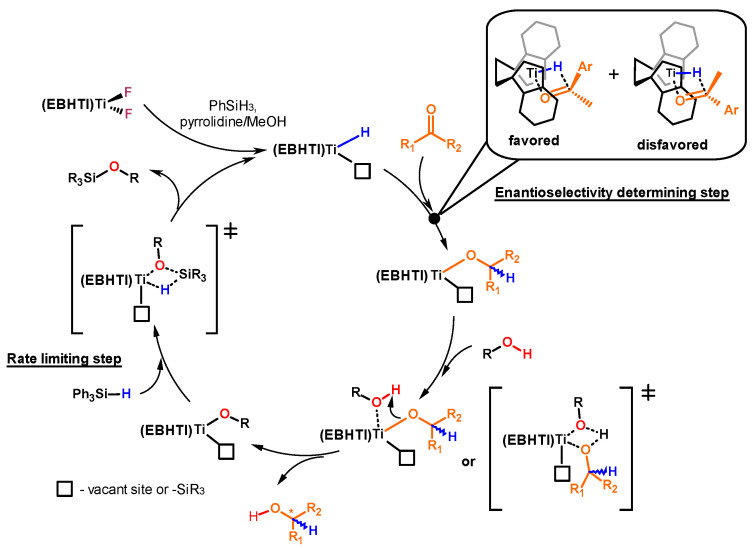
Mechanism of titanocene-catalyzed hydrosilylation of ketones by PhSiH_3_.

In the proposed mechanism illustrated in Scheme 74, the stereodifferentiating and rate-limiting steps of the reaction are distinct. The smaller the size of the alkyl group in the alcohol, the more rapid the σ-bond metathesis occurs in the rate-limiting step. Consequently, the added alcohol does not participate in the stereodifferentiating step; rather, it accelerates the reaction, thereby diminishing the probability of non-selective pathways during the hydrosilylation of ketones.

**Scheme 75:**
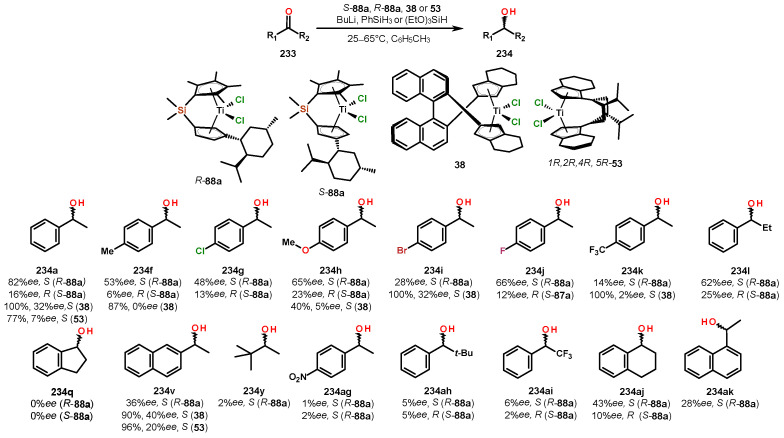
Reduction of aryl-substituted ketones by silanes catalyzed by Ti complexes *S*-**88a**, *R*-**88a**, *p*-*S*,*p*-*S*-**38**, and *1R*,*2R*,*4R*,*5R*-**53**.

The catalytic activity of *η*^5^-titanium complexes *S*-**88a**, *R*-**88a**, *p*-*S*,*p*-*S*-**38**, and *1R*,*2R*,*4R*,*5R*-**53** has been studied in the reduction reactions of aryl-substituted ketones using phenylsilane (Scheme 75) [74,168,169]. These catalysts exhibited varying activity (0–100% substrate conversion) and relatively low enantioselectivity (with a maximum of 82%*ee* in the case of acetophenone). Consequently, their practical significance is considerably lower compared to, for example, complexes with ethylene bridges [164,166]. In this case, the steric bulk of the complexes increases the enantioselectivity of the process; however, it significantly reduces their catalytic activity.

### 3.8. Asymmetric Hydrogenation of N-Substituted Compounds

**Scheme 76:**
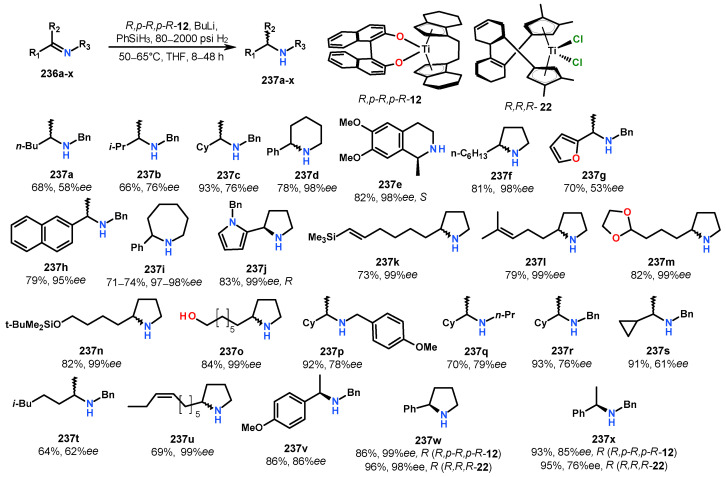
Asymmetric hydrogenation of imines catalyzed by *R*,*p*-*R*,*p*-*R*-**12** and *R*,*R*,*R*-**22**.

With the growing practical significance of enantiomerically pure nitrogen-containing compounds in the pharmaceutical and agrochemical industries, there is a pressing need to develop synthetic methods that exhibit high chemo-, regio-, and stereoselectivity. Asymmetric catalytic reduction of imines stands out as an effective method for the synthesis of chiral amines. Enantiomerically pure titanocene complexes *R*,*p*-*R*,*p*-*R*-**12** and *R*,*R*,*R*-**22** showed high activity and enantioselectivity in imine hydrogenation (Scheme 76) [53,170,171,172,173]. The catalytically active center in this reaction is presumably a titanocene (III) hydride, generated in situ through the interaction of titanocene complexes with an excess of triphenylsilane. Subsequently, phenylsilane was replaced with gaseous hydrogen, which proved to be a more effective stoichiometric reductant. The hydrogenation of imines **236a**–**v**, catalyzed by *R*,*p*-*R*,*p*-*R*-**12**, was carried out at hydrogen pressures of 80–2000 psi for durations ranging from 8 to 48 h [170,171,172,173] (Scheme 76). As a result, both cyclic and acyclic amines **237a**–**v** were formed with varying enantiomeric purity. High enantioselectivity (97–99%*ee*) was achieved with cyclic substrates, particularly ketimines, whereas acyclic compounds were reduced with moderate enantioselectivity (59–93%*ee*). The observed decrease in enantiomeric purity of the products is likely attributable to the fact that acyclic imines exist as mixtures of anti- and syn-isomers, which can interconvert during the reaction. Utilizing the same approach, 1-phenylpyrroline (**236w**) and N-(*R*-methylbenzylidene)benzylamine (**236x**) were reduced in the presence of the titanium complex *R*,*R*,*R*-**22** [53]. Under conditions of 150 bar H_2_ and 80 °C for 12 h, the corresponding secondary amines **237w** and **237x** were obtained with yields of 96% and enantioselectivities of 76–98%*ee*.

**Scheme 77:**
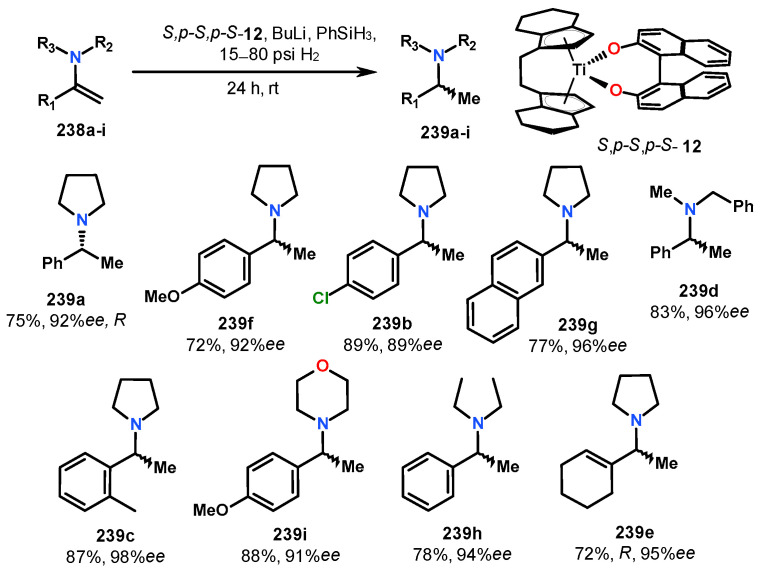
Hydrogenation of 1,1-disubstituted enamines catalyzed with *R*,*p*-*R*,*p*-*R*-**12**.

The binaphtholate Ti complex *S*,*p*-*S*,*p*-*S*-**12** can be effectively employed as a catalyst for the hydrogenation of 1,1-disubstituted enamines [174]. This reaction yielded enantiomerically enriched tertiary amines **239a**–**i** (>91%*ee*) with good yields (73–92%) (Scheme 77). Sterically hindered substrates reacted under elevated pressures (80 psi) and temperatures. Bulkier or bromine-containing substrates did not participate in the reaction.

**Scheme 78:**
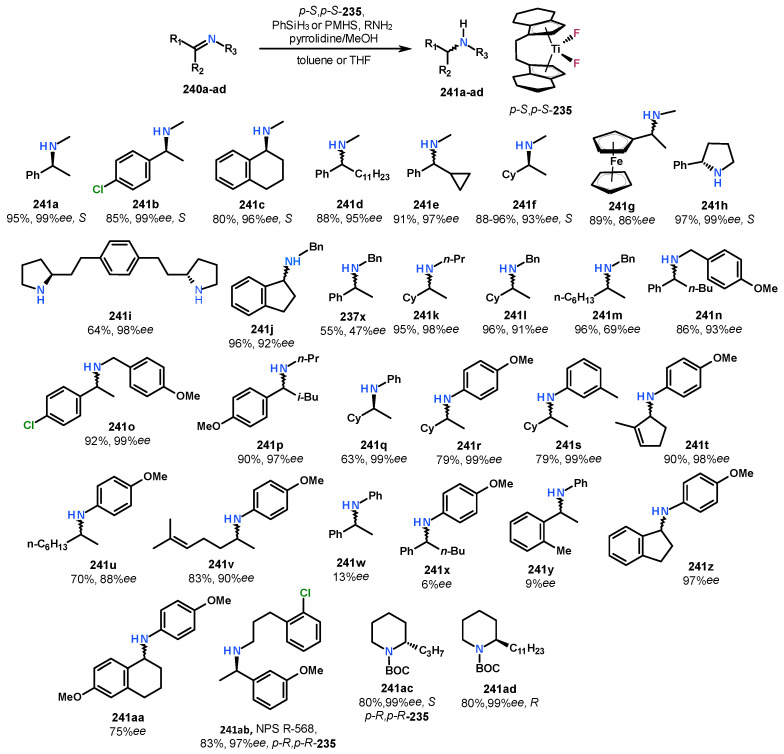
Asymmetric hydrosilylation of N-aryl-substituted imines using PhSiH_3_ or PMHS in the presence of Ti complex *p*-*S*,*p*-*S*-**235**.

The catalytic system based on titanocene difluoride *p*-*S*,*p*-*S*-**235** demonstrated high stereoselectivity in the hydrosilylation of imines, owing to the rapid conversion of the Ti-F bond to Ti-H under the influence of PhSiH_3_ or polymethylhydrosiloxane (PMHS) in a MeOH/pyrrolidine medium (Scheme 78) [175,176,177,178,179]. Consequently, the asymmetric hydrosilylation of N-aryl-substituted imines **240a**–**ad** using PhSiH_3_ or PMHS was developed, providing chiral amines **241a**–**ad** with yields of 55–97% and substantial enantiomeric excesses.

**Scheme 79:**
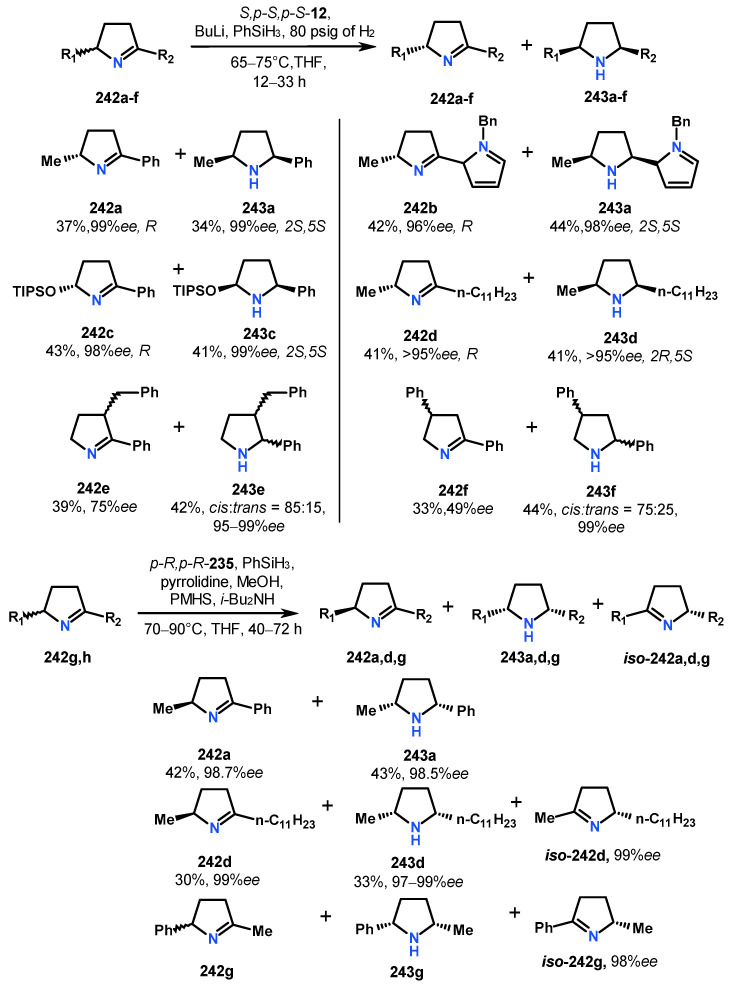
Hydrosilylation of *rac*-2,5-disubstituted pyrroles in the presence of the binaphtholate complex *S*,*p*-*S*,*p*-*S*-**12**.

Hydrosilylation of *rac*-2,5-disubstituted pyrroles **242a**–**f** in the presence of the binaphtholate complex *S*,*p*-*S*,*p*-*S*-**12** was accompanied by the kinetic resolution of the substrates, resulting in *cis*-pyrrolidines **243a**–**h** with yields of 34–44% and enantiomeric purity exceeding 95%*ee* (Scheme 79) [180]. The unreacted pyrrole was isolated with yields of 33–43%, also exhibiting a high enantiomeric excess of >95%*ee*. The reaction catalyzed by the difluoride complex *p*-*R*,*p*-*R*-**235** [181] resulted in a highly enantioselective (>94%*ee*) kinetic resolution of the starting substrates **242a**,**d**,**g**, yielding pyrrolidines **243a**,**d**,**g**, along with isomers of the starting pyrrole *iso*-**242a**,**d**,**g**.

**Scheme 80:**
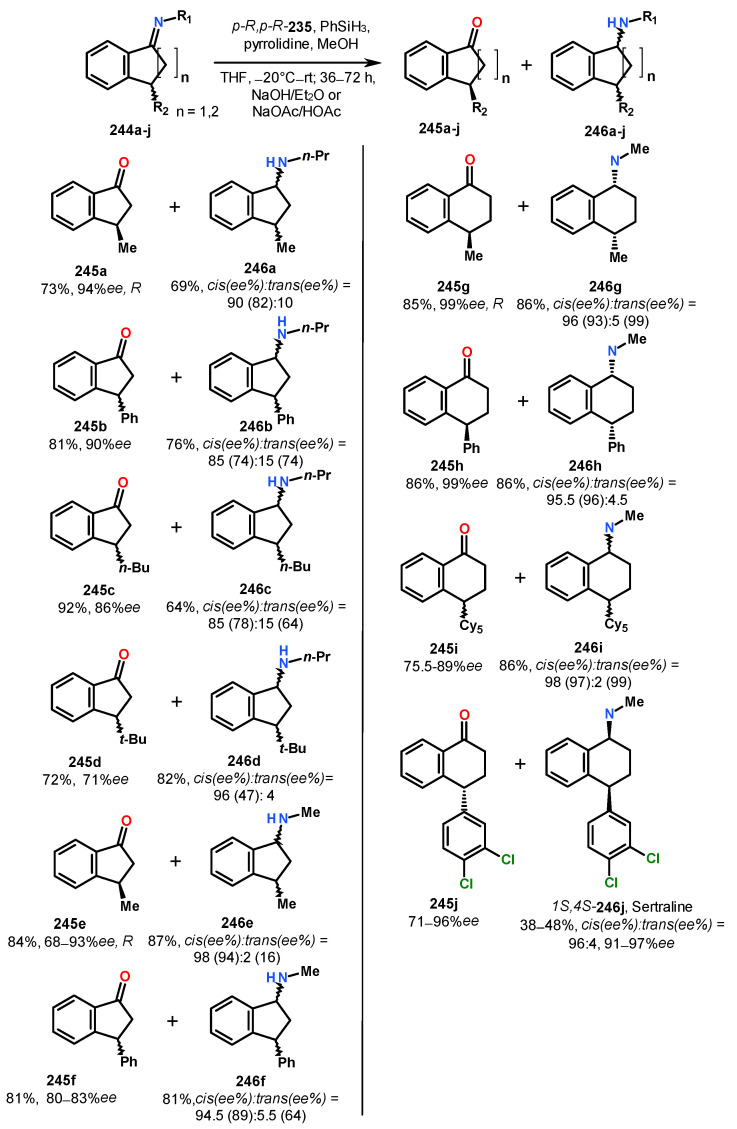
Kinetic resolution of N-alkyl imines derived from 3-substituted indanones and 4-substituted tetralones.

Optically active indanones or tetralones are also of significant interest as synthons for the synthesis of biologically active compounds. The kinetic resolution of N-alkyl (R = Me, *i*-Pr, *n*-Pr) imines derived from 3-substituted indanones **244a**–**f** and 4-substituted tetralones **244g**–**j** can be achieved through hydrosilylation in the presence of catalytic amounts of titanocene difluoride *p*-*R*,*p*-*R*-**235** or *p*-*S*,*p*-*S*-**235** (Scheme 80) [182]. Following the hydrolysis of the reaction mixture, ketones **245a**–**j** were obtained with enantiomeric purity of 68–99%*ee*, as well as amines **246a**–**j** exhibiting high diastereomeric (*cis* > 82%) and enantiomeric purity (47–97%*ee*). This method has been successfully applied in the diastereo- and enantioselective synthesis of the antidepressant sertraline *1S*,*4S*-**246j**.

**Scheme 81:**
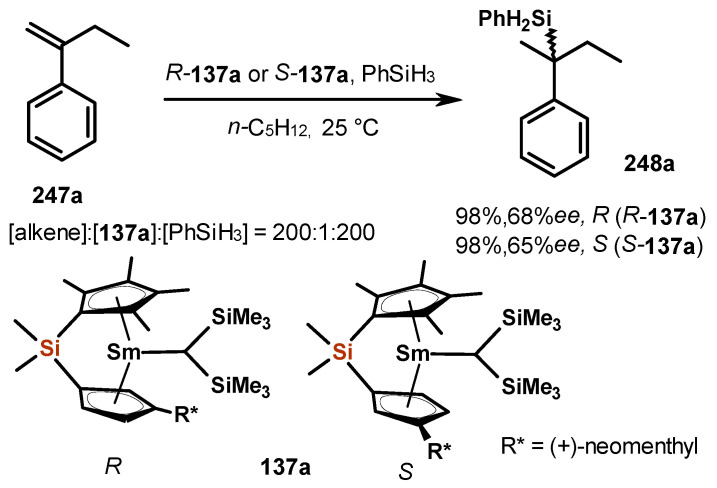
Hydrosilylation of phenylbutene by PhSiH_3_ in the presence of samarium complexes *R-* or *S*-**137a**.

An example of hydrosilylation of alkenes was demonstrated using phenylbutene **247a**, which, in the reaction with PhSiH_3_ in the presence of samarium complexes *R*- or *S*-**137a**, provided silane **248** with yields up to 98% and enantioselectivity of 65–68%*ee* (Scheme 81) [183].

### 3.9. Enantioselective Hydrogenation of Olefins

**Scheme 82:**
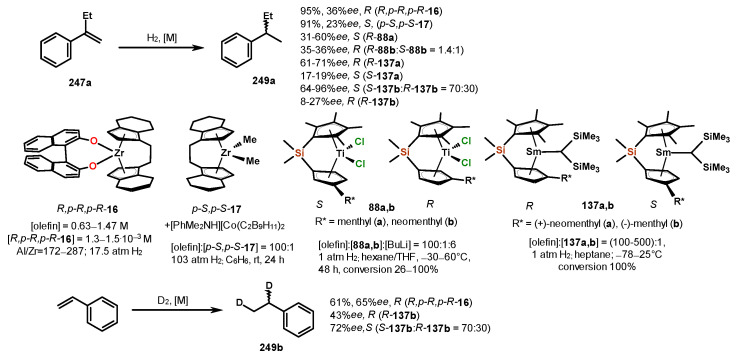
Hydrogenation of 2-phenylbut-1-ene and styrene deuteration catalyzed with Zr, Ti, or Sm complexes.

Asymmetric hydrogenation of olefins has been the subject of extensive research [184,185,186,187]. Enantiomerically pure *ansa*-complexes have also been evaluated as catalysts for this process. Pino et al. [188] (Scheme 82) demonstrated that the hydrogenation of 2-phenylbut-1-ene (**247a**), catalyzed by zirconocene binaphtholate *R*,*p*-*R*,*p*-*R*-**16** and MAO, yielded 2-phenylbutane **249a** with a good yield (over 89%) but low enantioselectivity (36%*ee*, *R*), while *rac*-**13** exhibited no catalytic activity. The hydrogenation of **247a** in the presence of enantiomerically pure zirconocene *p*-*S*,*p*-*S*-**17** and [PhMe_2_NH][Co(C_2_B_9_H_11_)_2_] produced phenylbutane with an enantioselectivity of 23%*ee*, *S* (Scheme 82) [189]. It is suggested that the low enantioselectivity in these systems arises from the minimal energy differences between states with different enantiofaces of the olefin at the catalytic site.

Upon activation with *n*-BuLi, diastereomers of complexes **88a**,**b** catalyzed the hydrogenation of 2-phenylbut-1-ene, with a moderate enantioselectivity of 35–60%*ee* [168].

Enantiomerically pure C_1_-symmetric Sm complexes *S*- and *R-***137a**,**b** exhibited higher enantioselectivity (up to 96%*ee*) in this reaction; the enantioselectivity was regulated by varying the reaction conditions and the ratio of enantiomers of the catalyst (Scheme 82) [83,183].

The polymerization of pentene-1 in the presence of D_2_ and *R*,*p*-*R*,*p*-*R*-**16** or *p*-*R*,*p*-*R*-**17** and MAO resulted in the formation of deuterated products with the general formula C_5_H_12−x_D_x_ (Scheme 53) [140,188]. Through fractional distillation, 1,2-dideuteriopentane (**249b**) was isolated with an enantiomeric excess of 23–35%*ee*, and the *R*-configuration of the product was determined by optical rotation. As a result of styrene deuteration, the *R*-enantiomer of **249b** was obtained with enantiomeric excesses of 65% and 43%*ee* when using *R*,*p*-*R*,*p*-*R*-**16** [188] and *R*-**137b** [83], respectively (Scheme 82). The mixture of *S*-**137b**/*R*-**137b** (70/30) allowed for the attainment of a relatively high enantiomeric excess of the *S*-product at the level of 72%*ee*.

**Scheme 83:**
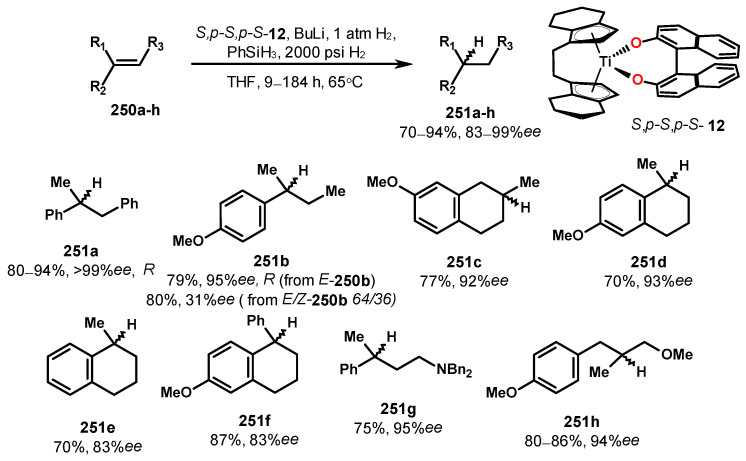
Hydrogenation of trisubstituted olefins in the presence of Ti complex *S*,*p*-*S*,*p*-*S*-**12**.

Trisubstituted olefins **250a**–**h** were hydrogenated under the 2000 psi of H_2_ in the presence of Ti complex *S*,*p*-*S*,*p*-*S*-**12**, activated with *n*-BuLi at 1 atm of H_2_ and stabilized by phenylsilane. The reaction afforded products **251a**–**h** with high yields of 70–94% and enantioselectivity up to 99%*ee* (Scheme 83) [190].

**Scheme 84:**
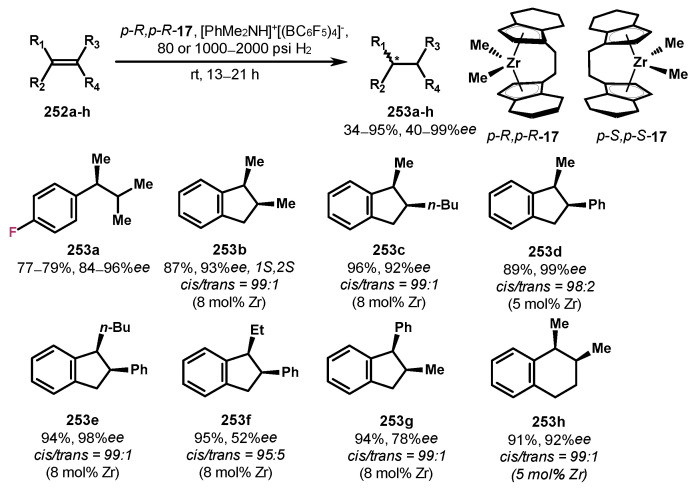
Hydrogenation of tetrasubstituted olefins in the presence of *p*-*R*,*p*-*R*- or *p*-*S*,*p*-*S*-**17** and [PhMe_2_NH]^+^[B(C_6_F_5_)_4_]^−^.

The hydrogenations of tetrasubstituted olefins were conducted at ambient temperature in aromatic hydrocarbons utilizing either *p*-*R*,*p*-*R*- or *p*-*S*,*p*-*S*- dimethylzirconocene complex **17** and activator [PhMe_2_NH]^+^[B(C_6_F_5_)_4_]^−^, applying hydrogen pressures of 80 or 1000–2000 psig (Scheme 84) [191]. Products **253a**–**h** exhibiting high diastereomeric (>95%, *cis*) and enantiomeric purity exceeding 90% were achievable at elevated pressures and catalyst concentrations. The reaction mechanism suggests the formation of highly reactive cationic species bearing a Zr-H bond, which engage in reactions with *re* face of alkene.

### 3.10. Hydroamination and Cyclization of Amino- and Phosphinoalkenes

**Scheme 85:**
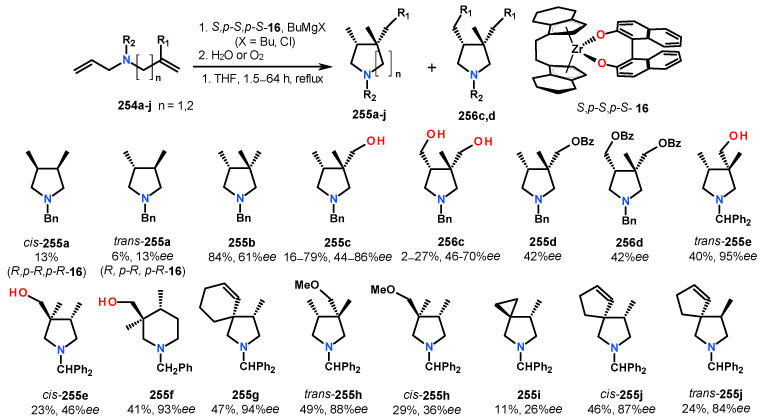
Synthesis of enantiomerically enriched N-heterocycles.

The binaphtholate complex *S*,*p*-*S*,*p*-*S*-**16** was employed in the synthesis of enantiomerically enriched N-heterocycles **255** and **256** (Scheme 85) [192]. In this reaction, the substituent at the double bond of the alkenyl group of the amine significantly influenced the course of the reaction. For example, the reaction of Bu_2_Mg with diallylbenzylamine in the presence of *R*,*p*-*R*,*p*-*R*-**16** produced heterocycle **255a** with low yield and enantiomeric excess (up to 13%). The reaction of substituted alkenes **254b**–**j** with BuMgCl, catalyzed by *S*,*p*-*S*,*p*-*S*-**16**, afforded five- and six-membered cyclic products **255a**–**j** and **256c**,**d** with yields of up to 84% (combined *cis-* and *trans*-) and enantioselectivity of up to 95%*ee*.

**Scheme 86:**
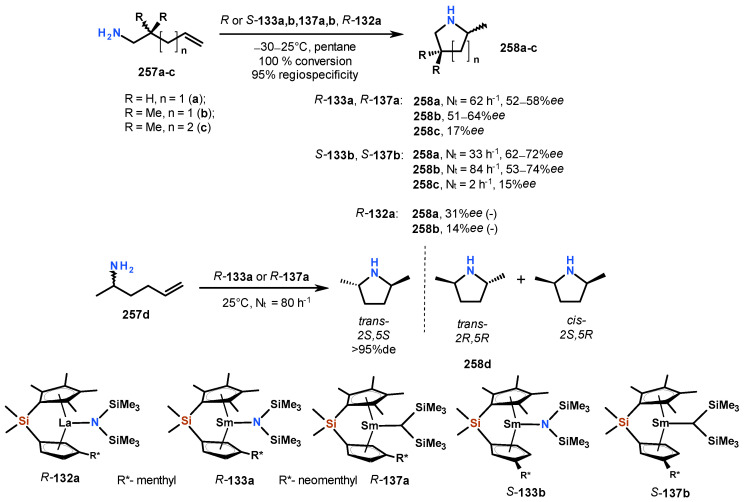
Asymmetric hydroamination/cyclization of aminoalkenes catalyzed with lanthanide *η*^5^-complexes.

The successful application of enantiomerically enriched *η*^5^-complexes of lanthanide metals has been demonstrated in the reaction of asymmetric hydroamination/cyclization of amino- and phosphinoalkenes **257a**–**e** [85,193,194,195,196] (Scheme 86). With a substrate-to-catalyst ratio of (40–200)/1, nearly 100% conversion of N- or P-substituted alkenes was achieved with over 95% regioselectivity. Thus, C_1_-symmetric complexes of Sm and La with menthyl- or neomenthyl-substituted *ansa*-cyclopentadienyl ligands catalyzed the hydroamination/cyclization of aminoalkenes **257a**–**c**, providing the corresponding pyrrolidines and piperidines **258a**–**c** with substrate conversion of up to 100% and enantiomeric purity < 74%*ee* [193,194,195]. The high diastereoselectivity of the reaction was demonstrated using 2-aminohexene **257d**, which was transformed into *trans*-2,5-dimethylpyrrolidine **258d** with >95%*de* in the presence of complexes **133a** and **137a**.

**Scheme 87:**
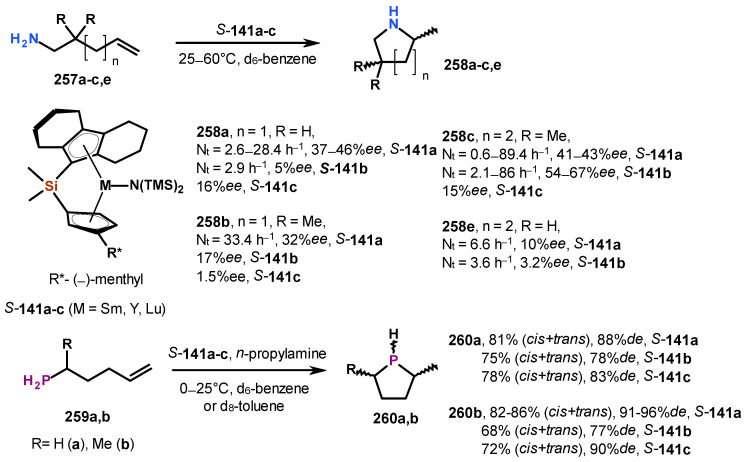
Asymmetric reduction/cyclization of amino- and phosphinoalkenes in the presence of complexes with octahydrofluorenyl ligand *S*-**141a**–**c**.

Furthermore, complexes with octahydrofluorenyl ligand *S*-**141a**–**c** (Sm, Y, Lu) exhibited activity and stereoselectivity in the hydroamination/cyclization of aminoalkenes **257a**–**c**,**e** (Scheme 87) [85]. However, these complexes proved to be less active and stereoselective compared to cyclopentadienyl complexes **133** and **137**. Enantioselectivity at the level of 67%*ee* was achieved only in the case of the sterically hindered substrate **257c** (Scheme 87). It was noted that the decrease in turnover frequency (N_t_) and selectivity for aminoalkenes **257a**,**b** correlated with the decrease in atomic radii of the lanthanide centers Sm^3+^—Y^3+^—Lu^3+^. The hydrophosphination/cyclization of phosphinoalkenes **259a**,**b** catalyzed by *S*-**141a**–**c** under the same conditions produced phospholanes **260a**,**b** with high diastereoselectivity of up to 96%*de* (Scheme 87).

**Scheme 88:**
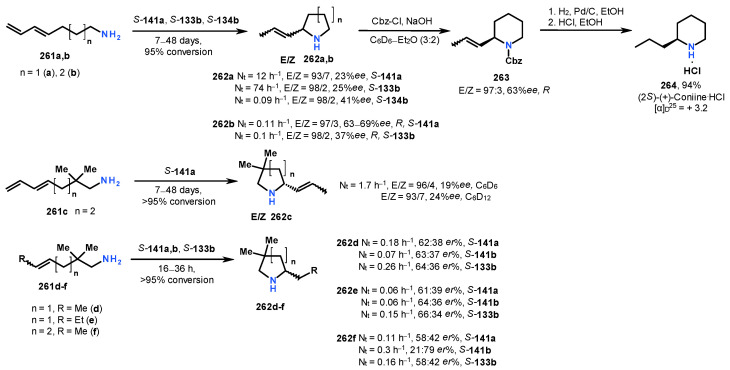
Cyclization of aminodienes and aminoalkenes in the presence of complexes *S*-**141a**, *S*-**133b** or *S*-**134b**.

The cyclization of aminodienes **261a**–**c** in the presence of complexes *S*-**141a**, *S*-**133b**, or *S*-**134b** yielded enantiomerically enriched substituted pyrrolidines and piperidines **262a**–**c** (Scheme 88) [196,197,198,199]. The natural alkaloid (+)-coniine·HCl (**264**) was obtained from the prochiral aminodiene **261b** via a two-step sequence in high isolated yield (*S*-**141a** was used as a catalyst). Catalytic hydroamination/cyclization of aminoalkenes **261d**–**f** with an internal double bond was also investigated (Scheme 88) [197,198]. The highest enantioselectivity was observed in the reaction catalyzed by Y complex *S*-**141b**.

### 3.11. Other Applications of Enantiomerically Enriched ansa-Complexes in Asymmetric Catalysis

**Scheme 89:**
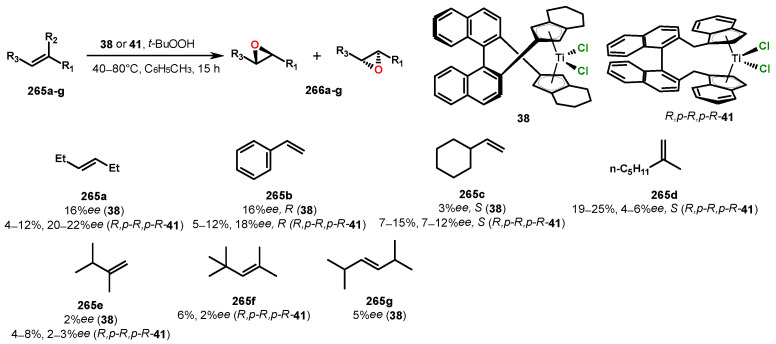
Asymmetric catalytic epoxidation of alkyl- and aryl-substituted alkenes.

Epoxides are valuable starting materials in organic synthesis due to their easy availability and high reactivity of the three-membered ring. Asymmetric catalytic epoxidation of alkyl- and aryl-substituted alkenes **265a**–**g** using *t*-BuOOH in toluene at 40–80 °C was conducted in the presence of ansa complexes **38** and *R*,*p*-*R*,*p*-*R*-**41** (Scheme 89). The enantiomeric purity of epoxides **266a**–**g** did not exceed 22%*ee*, with substrate conversion of 6–25% [62,200].

**Scheme 90:**
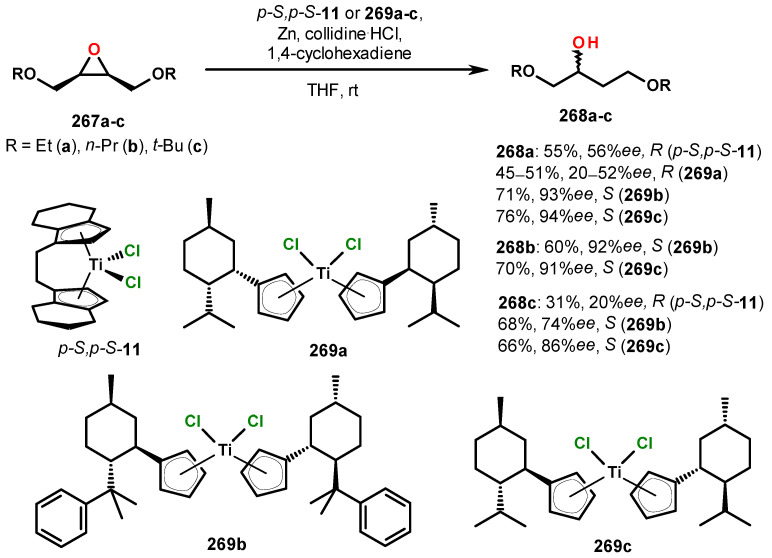
Ring-opening of epoxides in the presence of pure *ansa*-titanocene *p*-*S*,*p*-*S*-**11**.

Enantiomerically pure *ansa*-titanocene dichloride *p*-*S*,*p*-*S*-**11** catalyzed the ring-opening of epoxides **267a**–**c,** producing the corresponding alcohols **268a**–**c** with moderate enantioselectivity (56%*ee*), compared to conformationally labile neomenthylcyclopentadienyl titanium complexes **269a**–**c** (more than 90%*ee*) (Scheme 90) [201,202,203].

**Scheme 91:**
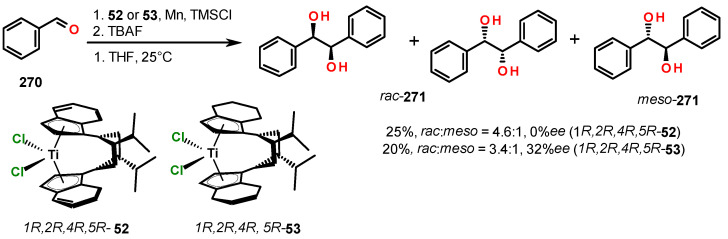
Homocoupling of benzaldehyde catalyzed with titanocene complexes **52** and **53**.

Chiral *bis*-(indenyl)-(**52**) and *bis*-(tetrahydroindenyl)-(**53**) titanocenes were tested as catalysts in the homocoupling of benzaldehyde **270**. As a result, pinacol-type compounds **271** were obtained (Scheme 91) [66]. It was found that the steric hindrance in tetrahydroindenyl complex **53** provided a lower yield but a higher enantiomeric excess of *rac*-1,2-diphenylethane-1,2-diol (32%*ee*).

**Scheme 92:**
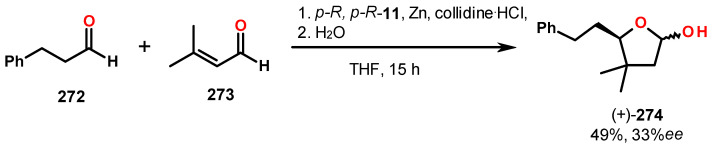
Ti-catalyzed synthesis of γ-lactol (+)-**285**.

The catalytic properties of the Ti complex *p*-*R*,*p*-*R*-**11** were demonstrated in the synthesis of γ-lactol (+)-**274**. The product was isolated with a yield of 49% and enantiomeric purity of 33%*ee* (Scheme 92) [204].

**Scheme 93:**
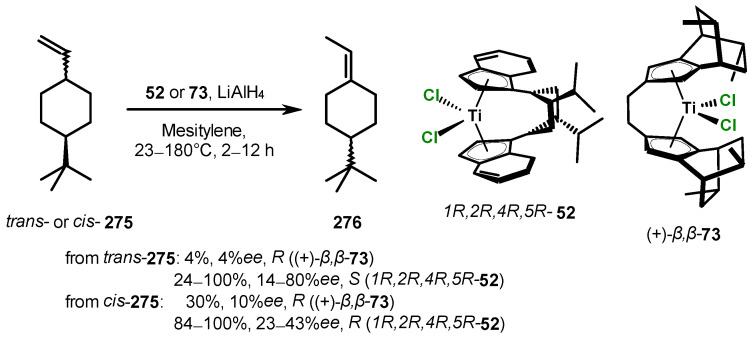
Enantioselective isomerization of *trans*- or *cis*-4-*tert*-butyl-1-vinylcyclohexanes in the presence of chiral titanium *ansa*-complexes **52** or **73**.

The potential application of chiral titanium *ansa*-complexes **52** or **73** in enantioselective isomerization of *trans*- or *cis*-4-*tert*-butyl-1-vinylcyclohexanes **275** to 4-*tert*-butyl-1-(ethylidene)cyclohexanes **276** was studied (Scheme 93) [71,205]. Complex **52** showed higher activity and enantioselectivity compared to the catalyst **73**. The reaction rate and the enantiomeric purity of the product were highly influenced by the temperature of the reaction. The decrease in enantiomeric purity observed at higher temperatures (180 °C) is believed to be caused by racemization through product equilibration, a phenomenon that is likely to be overcome with longer reaction times (12–120 h).

## 4. Conclusions

Chiral metal complexes of transition metals have occupied significant positions in organometallic chemistry and catalysis. Moreover, the potential applications for enantiomerically pure complexes include their use as sensors, antitumor agents, and other medicinal drugs, as well as templates for constructing 2D and 3D supramolecular structures. The introduction of *ansa*-*η*^5^-ligands significantly alters the electronic and spatial structure of the complexes, as well as their dynamics. Enantiomerically pure *ansa*-*η*^5^-complexes can be obtained through several approaches: synthesis from optically active precursors of *η*^5^-ligands, separation of diastereomers of the complexes utilizing enantiomerically pure agents, and the reaction of chiral σ-complexes with prochiral anions of *η*^5^-ligands. In organometallic synthesis, kinetic resolution of racemic complexes through interaction with derivatizing enantiomerically pure reagents is most commonly employed. However, the approach involving the fixation of ligands with a relatively rigid chiral bridge provides excellent results, allowing for the immediate production of enantiomers of metallocenes, including those that are less stable, such as indenyl complexes, which typically do not withstand separation using derivatizing reagents. The yield of the reaction and stereoselectivity in the synthesis of metal complexes is predetermined by a combination of numerous factors: the nature of the transition metal, the structure of the ligand, and the conditions of the synthesis (solvent, temperature, reagent ratio, etc.). As literature analysis shows, there is currently no universal solution to the problem of selecting these factors in the synthesis of desired enantiomerically pure metallocene structures.

The ansa-complexes of IVB and IIIB group metals demonstrate exceptional activity and stereoselectivity in the formation of metal–carbon, carbon–carbon, carbon–hydrogen, and carbon–heteroatom bonds. These complexes have proven to be effective catalysts for enantioselective alkene carbometalation, oligomerization, and polymerization. Notably, the stability of the spatial structure of *ansa*-complexes enhances stereoselectivity as the oligomeric chain grows (chain control). In contrast, chiral conformationally labile complexes can achieve high stereoselectivity at the initial stages of alkene insertion into catalytically active sites (site control); however, they struggle to maintain adequate control over stereoinduction as the chain lengthens. This area of research holds significant promise, particularly in advancing diastereo- and enantioselective oligomerization and polymerization of alkenes using conformationally stable complexes, facilitating the stereoselective synthesis of biologically active compounds and optically active polymers, which are in demand for the development of new materials.

Examples of asymmetric cycloaddition, cyclization, hydrosilylation, hydroamination, hydrogenation, etc., catalyzed by enantiomerically pure *ansa*-complexes of Group IIIB and IVB metals demonstrate the potential of these complexes as effective catalysts for practically important processes. Further advancement in this area necessitates an understanding of the stereoinduction and chiral recognition mechanisms that enable comprehension of the structure and dynamics of catalytically active centers, as well as the processes of substrate coordination and transformations. These studies are highly relevant, as their realization will facilitate a more optimal approach to the development of catalytic systems and processes based on them.

## Figures and Tables

**Table 1:** The synthesis of C_1_-symmetric enantiomerically pure *ansa*-complexes of lanthanides [84].

Entry	Complex	R*	Solvent for Synthesis (Crystallization)	Yield, %	Ratio of Epimers *R*:*S* (Solvent)
1	**127a** (La)	(+)-neomenthyl	THF (Et_2_O)	47	75:25 (THF)
2	**128a** (Nd)	(+)-neomenthyl	THF (Et_2_O)	65	74:26 (THF)
3	**129a** (Sm)	(+)-neomenthyl	THF (Et_2_O)	47	74:26 (THF)
4	**129a** (Sm)	(+)-neomenthyl	THF (Et_2_O)	58	34:66 (Et_2_O)
5	**130a** (Y)	(+)-neomenthyl	THF (Et_2_O)	63	74:26 (THF)
					46:54 (Et_2_O)
6	**131a** (Lu)	(+)-neomenthyl	THF (Et_2_O)	57	74:26 (THF)
					51:49 (Et_2_O)
7	**129b** (Sm)	(−)-menthyl	THF (Et_2_O)	60	>95:5 (Et_2_O)
					20:80 (THF)
9	**130b** (Y)	(−)-menthyl	THF (Et_2_O)	64	20:80 (THF)
					90:10 (Et_2_O)
10	**131b** (Lu)	(−)-menthyl	THF (Et_2_O)	70	20:80 (THF)
					85:15 (Et_2_O)
11	**130c** (Y)	(−)-phenylmenthyl	THF (Et_2_O)	48	6:94 (THF)
					83:17 (Et_2_O)
12	**129b**, *S* (Sm)	(−)-menthyl	THF, DME (Et_2_O)	88	
13	**131b**, *S* (Lu)	(−)-menthyl	THF, DME (Et_2_O)	89	
14	**129a**, *R* (Sm)	(+)-neomenthyl	THF, DME (Et_2_O)	54	

**Table 2:** Carbo-(**182**) and cycloalumination (**184**) of alkenes, catalyzed with enantiomerically pure metallocenes.

Catalyst	AlR_3_	Product	R′	*ee*%	Ref.
*p*-*S*,*p*-*S*-**187**	AlMe_3_	**182**	*n*-C_6_H_13_, *i*-Bu, Cy, Bn, Ph, C_4_H_8_OH, C_3_H_6_NEt_2_	65–85, *R*	[130]
		**182**	*n*-C_4_H_9_, *n*-C_6_H_13_	48–73, *R*	[134]
	AlEt_3_	**182**	R′ = *n*-C_6_H_13_	92, *R*	[135]
		**182**	*n*-C_4_H_9_, *n*-C_5_H_11_, *n*-C_6_H_13_, *n*-C_7_H_15_, *n*-C_8_H_17_, *i*-Bu, Cy, Cy_8_, Ph, Bn	33–68, *S*	[136]
		**184**	*n*-C_6_H_13_	33	[135]
		**184**	*n*-C_4_H_9_, Bn	5–11, *S*	[136]
			Cy, Cy_8_	45–57, *S*	
			Ph	31–40, *R*	
*p*-*R*,*p*-*R*-**13**	AlMe_3_	**182**	*n*-C_8_H_17_	6, *R*	[130]
*R*,*p*-*R*,*p*-*R*-**16**	AlMe_3_	**182**	*n*-C_6_H_13_	8, *R*	[130]
*p*-*S*,*p*-*S*-**13**	AlEt_3_	**182**	*n*-C_6_H_13_	51, *R*	[51]
		**184**	*n*-C_6_H_13_	11, *S*	
*S*,*p*-*S*,*p*-*S*-**16**	AlEt_3_	**182**	*n*-C_6_H_13_	15, *R*	[51]
		**184**	*n*-C_6_H_13_	26, *S*	
*p*-*S*,*p*-*S*-**18**	AlMe_3_	**182**	*n*-C_6_H_13_	58, *R*	[51]
	AlEt_3_	**182**	*n*-C_6_H_13_	50, *R*	
		**184**	*n*-C_6_H_13_	12, *S*	
*S*,*p*-*S*,*p*-*S*-**19**	AlEt_3_	**182**	*n*-C_6_H_13_	15, *R*	[51]
		**184**	*n*-C_6_H_13_	20, *S*	
*p*-*S*,*p*-*S*-**149**	AlMe_3_	**182**	Bn	29	[132]
*p*-*S*,*p*-*S*-**188a**	AlMe_3_	**182**	Bn	25	[132]
*p*-*S*,*p*-*S*-**188b**	AlMe_3_	**182**	Bn	29	[132]
*p*-*S*,*p*-*S*-**188c**	AlMe_3_	**182**	Bn	33	[132]
*p*-*S*,*p*-*S*-**189**	AlMe_3_	**182**	Ph	80	[132]
*p*-*R*,*S*-**190**	AlMe_3_	**182**	Ph,	19	[133]
			Bn	28	

**Table 3:** The polymerization of racemic α-olefins with bulky substituents **199a**–**f**, catalyzed with enantiomerically pure *S*-**116a**–**d** in the presence of MAO.

Entry	Alkene-1	Complex	Time, h	TOF, h^−1^	Conversion, %	s	%*ee*
1	**199a**	*S*-**116a**	18	72	24	2.8	13.3
2		*S*-**116a**	47	47	38.3	2.4	20.3
3		*S*-**116a**		60		2.6	
4		*S*-**116b**		266		2.3	
5		*S*-**116c**		374		2.5	
6		*S*-**116d**		280		3.2	
7	**199b**	*S*-**116a**	13.5	551	75	1.8	40
8		*S*-**116b**		299		1.8	
9		*S*-**116c**		706		1.9	
10	**199c**	*S*-**116a**	40.5	45.6	32.5	17.6	40.6
11		*S*-**116a**	69	34	42.4	15.9	58.6
12		*S*-**116a**		40		16.8	
13		*S*-**116b**		109		12.5	
14		*S*-**116c**		450		15	
15	**199e**	*S*-**116a**	16.5	77.6	64.4	1.1	7.6
16		*S*-**116a**	16.5	73.7	58.7	1.1	4.6
17		*S*-**116a**		75		1.1	
18		*S*-**116b**		111		1.2	
19		*S*-**116c**		633		1.2	
20	**199f**	*S*-**116a**		18		7.6	
21		*S*-**116d**		16		3.2	
22	**199d**	*S*-**116a**	22.7	55.8	37.8	2.0	16.2
23		*S*-**116a**	43	37	56.1	2.1	30.3
24		*S*-**116a**		37		2.1	
25		*S*-**116b**		49		6.4	
26		*S*-**116c**		111		1.6	
27		*S*-**116d**		988		8.5	

## Data Availability

Not applicable.
